# Osteology of the Basal Hadrosauroid *Eolambia caroljonesa* (Dinosauria: Ornithopoda) from the Cedar Mountain Formation of Utah

**DOI:** 10.1371/journal.pone.0045712

**Published:** 2012-10-15

**Authors:** Andrew T. McDonald, John Bird, James I. Kirkland, Peter Dodson

**Affiliations:** 1 Department of Earth and Environmental Science, University of Pennsylvania, Philadelphia, Pennsylvania, United States of America; 2 College of Eastern Utah Prehistoric Museum, Price, Utah, United States of America; 3 Utah Geological Survey, Salt Lake City, Utah, United States of America; 4 School of Veterinary Medicine, University of Pennsylvania, Philadelphia, Pennsylvania, United States of America; Royal Ontario Museum, Canada

## Abstract

**Background:**

*Eolambia caroljonesa* is known from copious remains from the lower Cenomanian Mussentuchit Member of the Cedar Mountain Formation in eastern Utah; however, the taxon has been only briefly described. Thus, we present herein a complete osteological description of *Eolambia*.

**Methodology/Principal Findings:**

The description of *Eolambia* presented here is based upon the holotype partial skeleton (CEUM 9758), paratype partial skull (CEUM 5212), and abundant disarticulated elements from two bonebeds that contain juvenile individuals. These remains allow the skeletal anatomy of *Eolambia* to be documented almost fully and a revised diagnosis to be proposed.

**Conclusions/Significance:**

The description provided here facilitates comparisons between *Eolambia* and other iguanodontians and allows *Eolambia* to be coded for additional characters in phylogenetic analyses. The close affinity between *Eolambia* and *Probactrosaurus gobiensis* from the Early Cretaceous of China supports previous hypotheses of faunal interchange between Asia and North America in the early Late Cretaceous.

## Introduction

The fossil record of non-hadrosaurid members of Iguanodontia, i.e. basal iguanodonts, from North America has grown dramatically in recent years and now rivals those of Europe and eastern Asia. Most of the recently-named taxa, such as *Dakotadon* [1], [2], *Theiophytalia* [3], *Planicoxa* [4], [5], *Cedrorestes* [6], [7], *Iguanacolossus* [7], and *Hippodraco* [7], are of Early Cretaceous age. Only three basal iguanodonts are known from the early Late Cretaceous of North America: *Eolambia* from the lower Cenomanian Mussentuchit Member of the Cedar Mountain Formation [8], [9], *Protohadros* from the middle Cenomanian Woodbine Formation [10], and *Jeyawati* from the middle Turonian Moreno Hill Formation [11].

*Eolambia caroljonesa* was named by Kirkland in 1998 [8] based upon the holotype partial skeleton, paratype skull, and several other specimens. Although Kirkland [8] originally suggested that *Eolambia* was a basal lambeosaurine hadrosaurid, Head [12] further described specimens in the OMNH collection and found *Eolambia* to be a basal hadrosauroid outside of Hadrosauridae, a position supported by more recent and more extensive phylogenetic analyses of basal iguanodont relationships [7], [13], [14]. All known material of *Eolambia* comes from the Mussentuchit Member (lower Cenomanian) of the Cedar Mountain Formation in eastern Utah [8], [9], [15], [16].

The description of *Eolambia* herein is based upon the adult holotype and paratype specimens and juvenile material from the *Eolambia* #2 and Willow Springs 8 bonebeds in the CEUM collection. Based upon a count of right dentaries, the minimum number of individuals (MNI) preserved in the *Eolambia* #2 bonebed is twelve, while the MNI of the Willow Springs 8 bonebed is four, based upon the number of right humeri. The stratigraphy, taphonomy, and age of the *Eolambia* #2 bonebed have been thoroughly explicated by Garrison et al. [9]. An age of 96.7±0.5 Ma (lower Cenomanian) [16] was obtained from a bentonite layer that extends through the quarry interval [9].

Institutional Abbreviations: AMNH, American Museum of Natural History, New York, NY, USA; CEUM, College of Eastern Utah Prehistoric Museum, Price, UT, USA; CM, Carnegie Museum of Natural History, Pittsburgh, PA, USA; DMNH, Denver Museum of Nature and Science, Denver, CO, USA; IRSNB, Institut royal des Sciences naturelles de Belgique, Brussels, Belgium; MIWG, Museum of Isle of Wight Geology (Dinosaur Isle Museum), Sandown, UK; MNHN, Muséum national d'Histoire naturelle, Paris, France; NHMUK, The Natural History Museum, London, UK; OMNH, Sam Noble Oklahoma Museum of Natural History, Norman, OK, USA; OXFUM, Oxford University Museum of Natural History, Oxford, UK; SDSM, South Dakota School of Mines and Technology, Rapid City, SD, USA; SMU, Southern Methodist University Shuler Museum of Paleontology, Dallas, TX, USA; UMNH, Natural History Museum of Utah, Salt Lake City, UT, USA; USNM, National Museum of Natural History, Washington, DC, USA; YPM, Yale Peabody Museum of Natural History, New Haven, CT, USA.

## Results

### Systematic Paleontology

Dinosauria Owen, 1842 [17]

Ornithischia Seeley, 1887 [18]

Ornithopoda Marsh, 1881 [19]

Iguanodontia Dollo, 1888 [20] *sensu* Sereno, 2005 [21]

Ankylopollexia Sereno, 1986 [22] *sensu* Sereno, 2005 [21]

Styracosterna Sereno, 1986 [22] *sensu* Sereno, 2005 [21]

Hadrosauriformes Sereno, 1997 [23] *sensu* Sereno, 1998 [24]

Hadrosauroidea Cope, 1870 [25] *sensu* Sereno, 2005 [21]

*Eolambia caroljonesa* Kirkland, 1998 [8]

#### Holotype (after Kirkland, 1998 [8])

CEUM 9758, partial adult skull and associated postcranium, from CEUM Locality 42em366v (Carol's Site, discovered by Carol and Ramal Jones), east of Castle Dale, Utah. The elements of the holotype are also numbered individually, but the collective number CEUM 9758 is used herein for clarity.

#### Paratypes (after Kirkland, 1998 [8] and Head, 2001 [12])

CEUM 5212, partial adult skull, from CEUM Locality 42em369v (the elements of this paratype specimen are also numbered individually, but the collective number CEUM 5212 is used exclusively herein); two partial juvenile skeletons from OMNH Locality v237; partial juvenile skeleton from OMNH Locality v824; OMNH 27749, sacrum and ischium, from OMNH Locality v696; OMNH 24389, isolated left ischium, from OMNH Locality v214; OMNH 32812, scapula and two caudal vertebrae, from OMNH Locality v866.

#### Referred Material

CEUM 8786, isolated left femur from the same locality as CEUM 9758 (42em366v), approximately 100 meters southwest of the holotype quarry at the same stratigraphic level. Disarticulated juvenile cranial and postcranial material (CEUM collection, MNI = 12) from the *Eolambia* #2 quarry (CEUM locality 42em432v) in Mussentuchit Wash, south of Emery, Utah. Disarticulated juvenile cranial and postcranial material (CEUM collection, MNI = 4) from the Willow Springs 8 quarry (CEUM locality 42Em576v) in Mussentuchit Wash, south of Emery, Utah.

#### Specific diagnosis (as for genus by monotypy)

Characters based solely upon juvenile specimens are marked with an asterisk. Basal hadrosauroid diagnosed by the following unique combination of characters: **rostral ramus of dentary deepens in lateral view** (also in *Ouranosaurus* [26], *Protohadros* [10], and *Bactrosaurus* (AMNH 6553)); dorsal end of coronoid process expanded along only rostral margin* (also in *Fukuisaurus* [27], *Kukufeldia* [28], *Iguanodon bernissartensis* (MIWG 1997.55) [29], *Mantellisaurus* (NHMUK R5764) [30], *Penelopognathus* [31], *Protohadros* [10], and *Jeyawati* [11], but different from *Probactrosaurus gobiensis*, in which it is expanded along both the rostral and caudal margins [13]); **ventral margin of maxillary tooth row concave in lateral view** (also in *Iguanacolossus* [7], *Dakotadon* (SDSM 8656) [1], *Fukuisaurus* [27], *Iguanodon bernissartensis* [29], *Mantellisaurus* (NHMUK R5764) [30], *Ouranosaurus* [26], *Altirhinus* [32], *Equijubus* [33], *Probactrosaurus gobiensis* [13], and *Shuangmiaosaurus* [34], but different from *Xuwulong* [35], *Protohadros* [10], and *Jeyawati* [11], in which it is straight); **dentary teeth with a primary ridge and single mesial accessory ridge** (present in holotype CEUM 9758; also in *Protohadros* [10], *Levnesovia* [36] *Tethyshadros* [37], and some specimens of *Bactrosaurus* [38]); straight shaft of ischium* (also in *Uteodon aphanoecetes* [5], [39], *Mantellisaurus* (IRSNB 1551), NHMUK R3741 (the so-called “Mantel-piece”), *Altirhinus* [32], *Bactrosaurus* [38], and *Gilmoreosaurus* [40]); **straight distal half of femoral shaft** (also in *Hypselospinus* (NHMUK R1629 [41]), *Iguanodon bernissartensis* [29], *Ouranosaurus* [26], “*Probactrosaurus*” *mazongshanensis* [42], *Nanyangosaurus* [43], *Bactrosaurus* [38], *Gilmoreosaurus* [40], *Tanius* [44], and *Tethyshadros* [37]).

#### Distribution and horizon

All specimens of *Eolambia caroljonesa* have been found in Emery County, Utah, in the Mussentuchit Member, Cedar Mountain Formation (lower Cenomanian) [8], [9], [15], [16]. More precise locality data are on file at CEUM and OMNH.

### Description

The following descriptions and figures are based upon holotype CEUM 9758, paratype CEUM 5212, and many examples from the *Eolambia* #2 (Eo2) and the Willow Springs 8 (WS8) bonebeds in the CEUM collection. Representative specimens were chosen based upon their capacity to contribute information to an osteological description of *Eolambia*, i.e. the most complete and best preserved examples of each known skeletal element were used. It is noted throughout the figure captions whether an illustrated element is from holotype CEUM 9758, paratype CEUM 5212, the Eo2 bonebed, or the WS8 bonebed. Measurements of select elements of *Eolambia* are given in the online supplementary information (Table S1).

**Figure 1:**
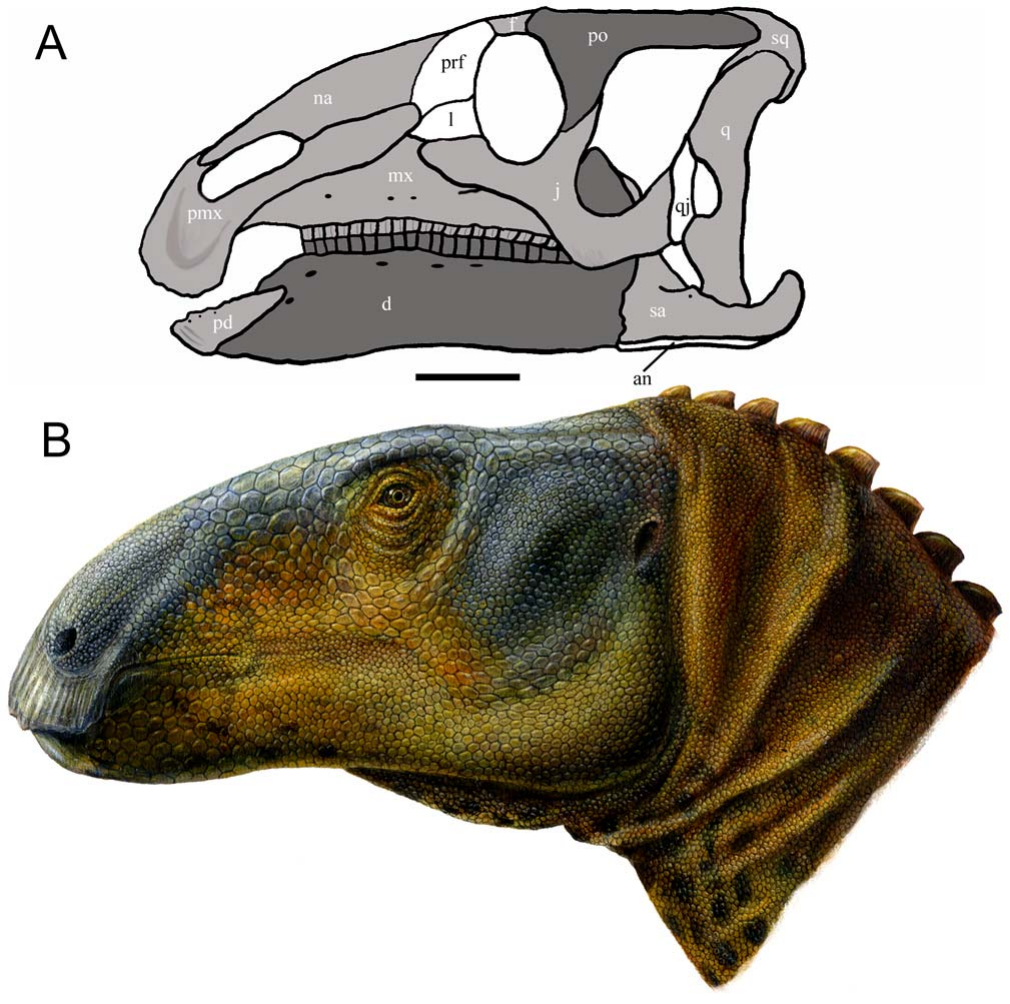
Reconstruction and restoration of the skull of *Eolambia*. (A) Skull reconstruction of *Eolambia* in left lateral view by the lead author. Bones in white are unknown, bones in dark grey are based primarily upon the adult holotype (CEUM 9758) or paratype (CEUM 5212), and bones in light grey are based primarily upon juvenile elements from the Eo2 and WS8 bonebeds. Sutures and points of contact between bones are marked in black. Scale bar equals 10 cm; scale is calibrated with the dentary of CEUM 9758. (B) Life restoration of the head of *Eolambia* by Lukas Panzarin. *Abbreviations*: *an*, angular; *d*, dentary; *f*, frontal; *j*, jugal; *l*, lacrimal; *mx*, maxilla; *na*, nasal; *pd*, predentary; *pmx*, premaxilla; *po*, postorbital; *prf*, prefrontal; *q*, quadrate; *qj*, quadratojugal; *sa*, surangular; *sq*, squamosal.

The abundant and well preserved cranial elements of *Eolambia caroljonesa* facilitated a new reconstruction of the skull and life restoration of the head (Fig. 1). The skull reconstruction (Fig. 1A) differs considerably from that of Kirkland (Fig. 11 in [8]), but closely resembles skull reconstructions of the Early Cretaceous Asian hadrosauroids *Equijubus normani* (Fig. 1C in [33]) and *Probactrosaurus gobiensis* (Fig. 3 in [13]). As it is based upon a combination of adult and modified rescaled juvenile elements, the new reconstruction of *Eolambia* should be considered tentative pending the discovery of more complete adult cranial remains and should not be used to code *Eolambia* in phylogenetic analyses. The skull reconstruction is simply an idealized schematic representation.

### Predentary

**Figure 2:**
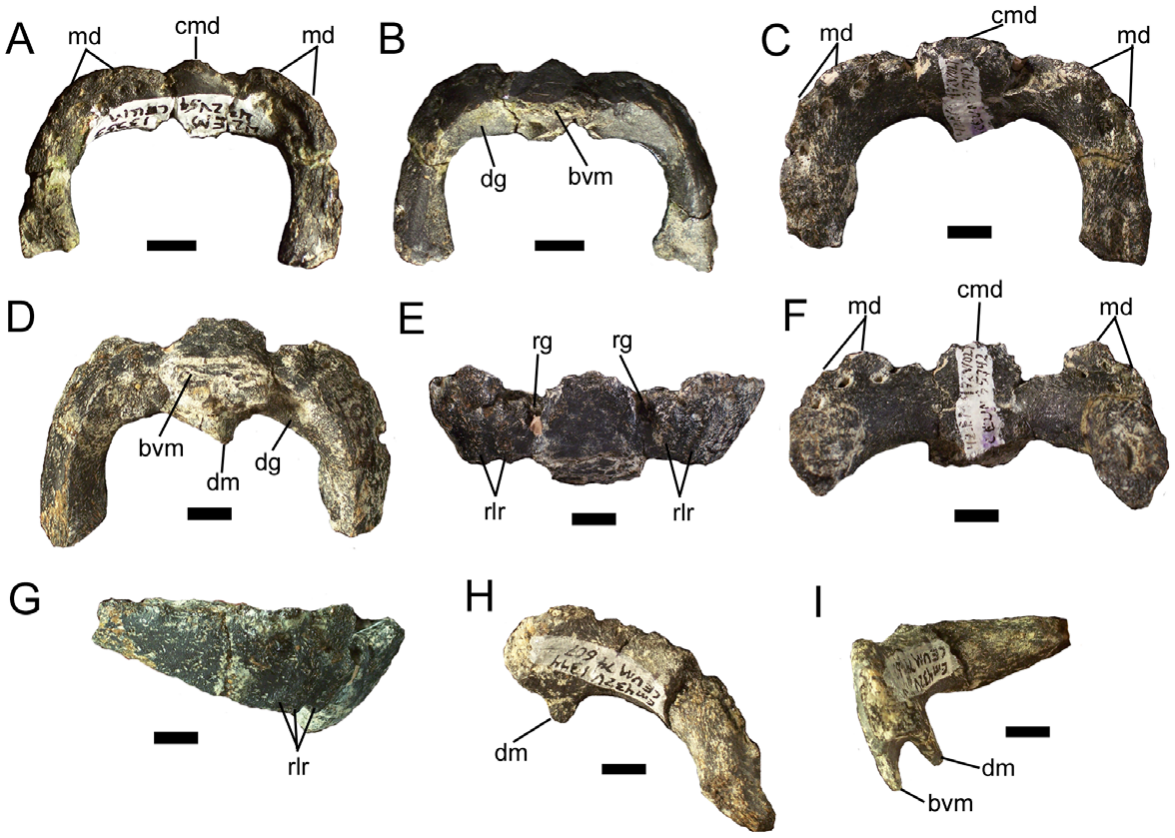
Predentaries of *Eolambia*. CEUM 13355 (Eo2) in (A) dorsal and (B) ventral views. CEUM 35742 (Eo2) in (C) dorsal, (D) ventral, (E) rostral, (F) caudal, and (G) right lateral views. CEUM 74607 (Eo2) in (H) dorsal and (I) left lateral views. *Abbreviations*: *bvm*, base of ventromedial process; *cmd*, central marginal denticle; *dg*, dentary groove; *dm*, dorsomedial process; *md*, marginal denticle; *rg*, rostral groove; *rlr*, rostrolateral ridge. Scale bars equal 1 cm.

The predentary is arcuate with rounded rostrolateral corners in dorsal and ventral views (Fig. 2A–D). The lateral processes extend caudally from the rostral margin; the right and left lateral processes are roughly parallel to each other, though both curve medially towards their caudal ends (Fig. 2A–D). The lateral processes are mediolaterally expanded at their caudal ends, with a slight indentation along the caudal margin of each process (Fig. 2A, B). A broad, shallow groove extends along the ventral surface of each lateral process, forming the surface along which the predentary articulates with and overlaps the lateral surface of the rostral ramus of the dentary (Fig. 2B, D). The ventromedial process of the predentary is broken at or near its base in all known predentaries of *Eolambia*, making it impossible to determine whether the process was bifurcated (Fig. 2B, D). The dorsomedial process of CEUM 74607 is intact and is a small triangular tab extending caudoventrally from the ventral margin of the predentary (Fig. 2H, I), as in *Iguanodon bernissartensis* [29], *Mantellisaurus* [30], *Ouranosaurus* [26], *Probactrosaurus gobiensis* [13], *Protohadros* [10], *Levnesovia* [36], *Bactrosaurus* [38], and *Gilmoreosaurus* [40].

The rostral margin of the predentary slopes rostrodorsally towards its dorsal margin (Fig. 2E, G). Two deep grooves originate on either side of the central marginal denticle and extend ventrolaterally on either side of the midline of the rostral margin of the predentary (Fig. 2E). Lateral to these grooves, the rostrolateral margins of the predentary are punctuated by a series of three low ridges that extend from the ventral margin of the predentary and terminate at the bases of the marginal denticles (Fig. 2E, G). The marginal denticles are rostrocaudally compressed tabs that increase in diameter and dorsoventral height towards the central denticle, which is the most prominent (Fig. 2A, C, F), as in *Probactrosaurus gobiensis* [13] and *Altirhinus* [32]. Where preserved, the dorsal margins of the denticles are rounded (Fig. 2F). A series of neurovascular foramina extends along the bases of the denticles, parallel to the denticle row (Fig. 2A, C, F); these foramina are visible on both the external and internal surfaces of the predentary.

### Dentary

**Figure 3:**
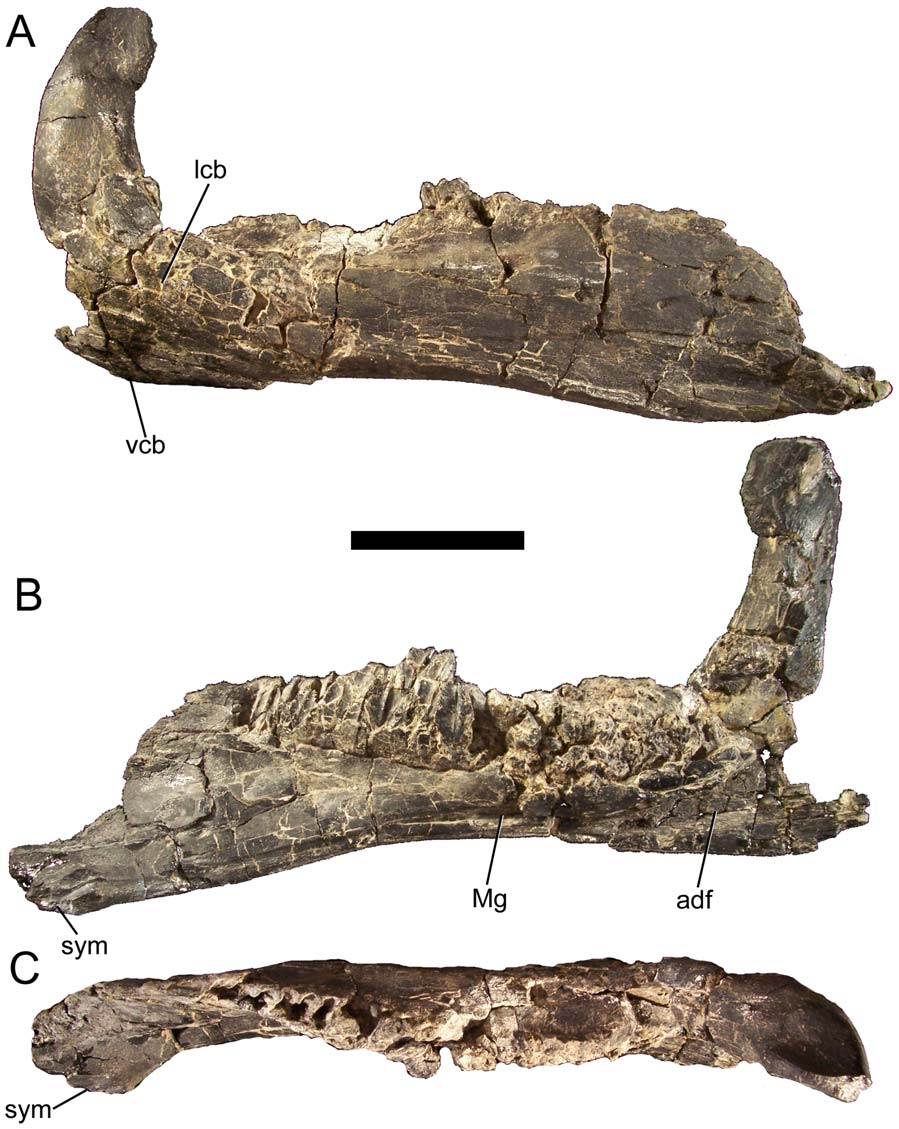
Dentary of *Eolambia*. Right dentary of CEUM 9758 (holotype) in (A) lateral, (B) medial, and (C) dorsal views. *Abbreviations*: *adf*, adductor fossa; *lcb*, lateral convex bulge; *Mg*, Meckelian groove; *sym*, symphysis; *vcb*, ventral convex bulge. Scale bar equals 10 cm.

**Figure 4:**
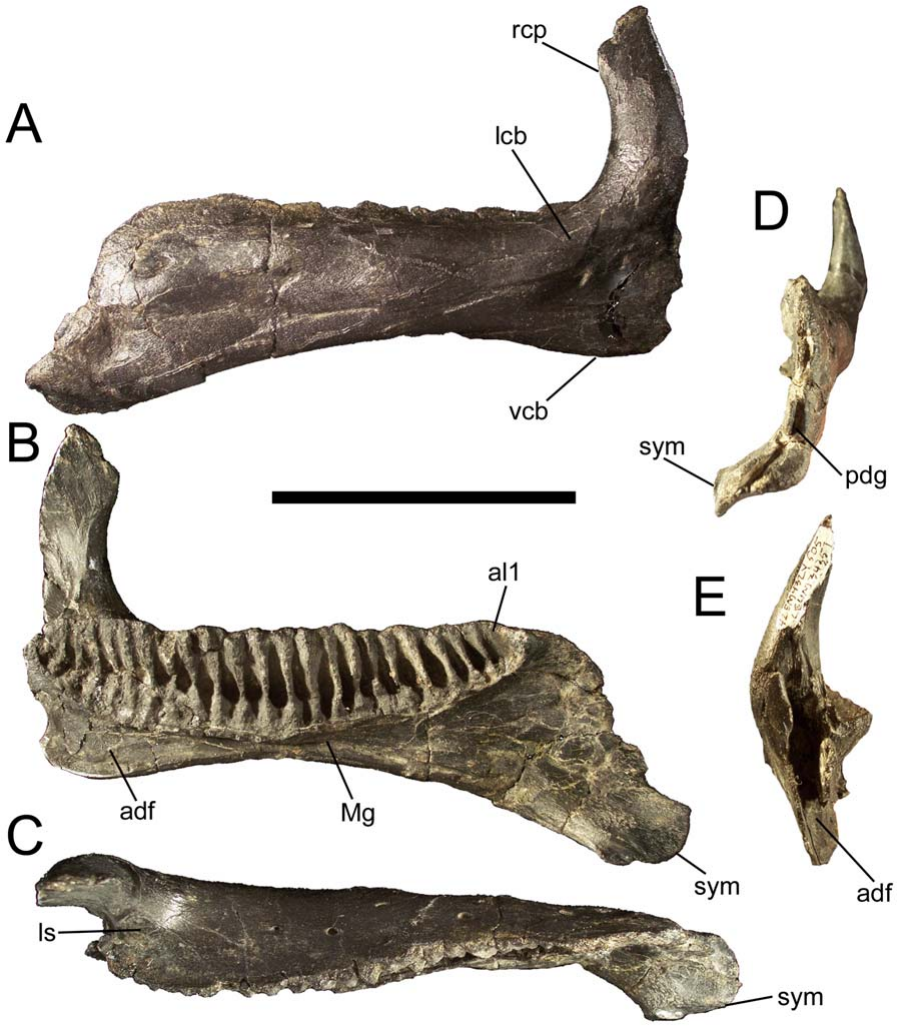
Dentary of *Eolambia*. Left dentary CEUM 34357 (Eo2) in (A) lateral, (B) medial, (C) dorsal, (D) rostral, and (E) caudal views. *Abbreviations*: *adf*, adductor fossa; *al1*, rostral-most alveolus; *lcb*, lateral convex bulge; *ls*, lateral shelf between the tooth row and base of the coronoid process; *Mg*, Meckelian groove; *pdg*, groove for contact with predentary; *rcp*, rostral expansion of the coronoid process; *sym*, symphysis; *vcb*, ventral convex bulge. Scale bar equals 10 cm.

The dorsal and ventral margins of the dentary are parallel for approximately half the length of the bone but diverge towards the rostral ramus, with the ventral margin strongly inflected ventrally (Figs. 3A, B; 4A, B). The rostral ramus of the dentary deepens in lateral and medial view, as in *Ouranosaurus* [26] and *Protohadros* [10]. The dentary symphysis is oriented rostrolaterally to caudomedially relative to the lateral surface of the dentary in dorsal view (Figs. 3C, 4C). Lateral to the symphysis, a narrow groove for articulation with the corresponding lateral process of the predentary extends caudodorsally along the rostrolateral surface of the rostral ramus (Fig. 4D). There is a short diastema between the caudal end of the predentary groove and the rostral-most alveolus, as in *Ouranosaurus* [26], *Altirhinus* [32], *Jinzhousaurus* [45], *Equijubus* [33], *Ratchasimasaurus* [46], *Xuwulong* [35], *Probactrosaurus gobiensis* [13], *Protohadros* [10], and *Jeyawati* [11].

The dentary tooth row is straight along its dorsal margin in lateral view (Fig. 4A). The tooth row is straight for most of its length in dorsal view but is bowed medially near its caudal end, with the most caudal alveoli curving laterally (Figs. 3C, 4C). A row of neurovascular foramina extends along the dorsolateral surface of the dentary roughly parallel to the tooth row (Fig. 4C). The dentary alveoli are narrow, parallel-sided grooves (Fig. 4B). There are approximately 25–30 alveoli in the right dentary of holotype CEUM 9758, while complete juvenile dentaries from the Eo2 bonebed have a minimum of 18 (e.g., CEUM 35482 and 35607) and a maximum of 22 (e.g., CEUM 34357 [Fig. 4B]) alveoli. The tooth row extends caudally past the long axis of the coronoid process but not past the caudal margin of the process (Fig. 4B). The caudal end of the tooth row is separated from the medial surface of the coronoid process by a shelf of bone (Fig. 4C). Ventral to the base of the coronoid process, the ventral margin of the dentary forms a convex bulge. The coronoid process itself arises from another bulge on the lateral surface of the dentary and projects vertically (Figs. 3A, 4A), as in *Altirhinus* [32], *Jinzhousaurus* [45], *Penelopognathus* [31], *Probactrosaurus gobiensis* [13], *Protohadros* [10], *Jeyawati* [11], and *Bactrosaurus* [38]. The dorsal end of the process is expanded along its rostral margin, with the rostrocaudally widest point in the coronoid process ventral to its apex (Fig. 4A). The Meckelian groove originates ventral to the base of the coronoid process and exhibits its greatest width and depth, forming the rostral portion of the adductor fossa (Figs. 3B; 4B, E). The Meckelian groove becomes progressively narrower towards the rostral end of the dentary and terminates caudolateral to the symphysis (Figs. 3B; 4B).

**Figure 5:**
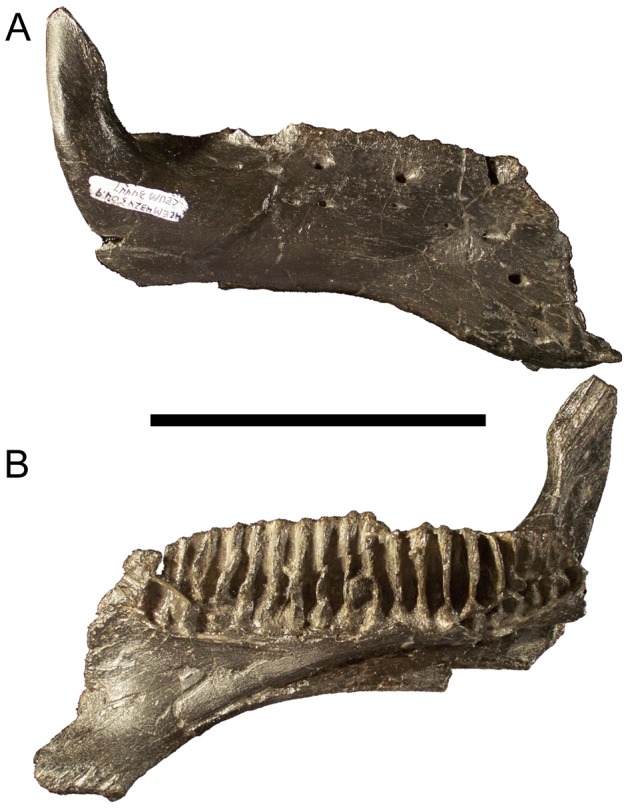
Dentary of indeterminate basal hadrosauroid. Right dentary CEUM 34447 (Eo2) in (A) lateral and (B) medial views. Scale bar equals 10 cm.

It is necessary to call attention to CEUM 34447, a juvenile right dentary from the Eo2 bonebed (Fig. 5). This dentary appears considerably deeper for its length than the dentary of the holotype (CEUM 9758), other juvenile dentaries from the Eo2 bonebed (e.g., CEUM 34357, 35482, 35714, 52995) and a juvenile dentary from the WS8 bonebed (CEUM 52139). These dentaries have midpoint depth/total dentary length ratios of approximately 0.19–0.24 (mean∼0.21), while that of CEUM 34447 is approximately 0.31. CEUM 34447 bears one of the two dentary features in the unique combination of characters that diagnoses *Eolambia* (dorsal end of coronoid process expanded along only its rostral margin), but lacks the other feature (rostral ramus of dentary deepens in lateral view; compare Fig. 5 with Figs. 3A, B and 4A, B); the dorsal and ventral margins of the dentary remain parallel in CEUM 34447. It is possible that CEUM 34447 represents a distinct taxon of basal hadrosauroid, one that differs from *Eolambia caroljonesa* in dentary morphology at least. However, the other cranial and postcranial bones from the Eo2 quarry do not suggest the presence of more than one taxon. Thus, it must also be admitted that CEUM 34447 could be an aberrant individual of *E. caroljonesa*. Caution demands that CEUM 34447 simply be regarded as an indeterminate basal hadrosauroid.

### Surangular

**Figure 6:**
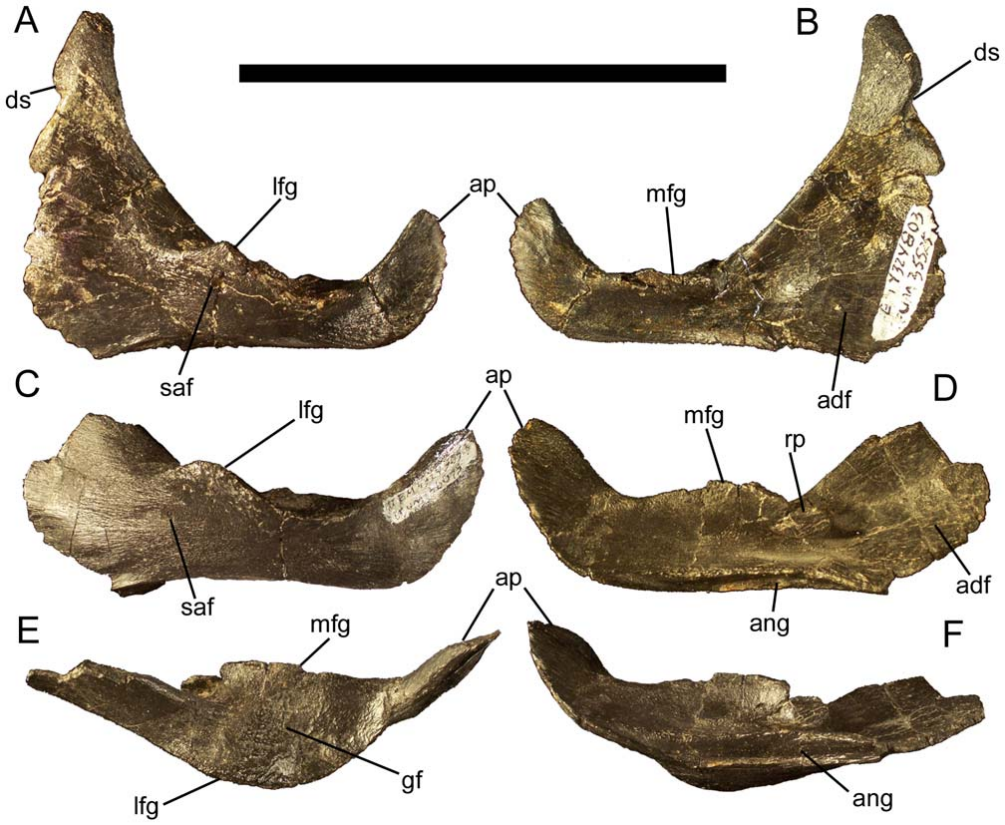
Surangulars of *Eolambia*. Left surangular CEUM 35525 (Eo2) in (A) lateral and (B) medial views. Left surangular CEUM 36073 (Eo2) in (C) lateral, (D) medial, (E) dorsal, and (F) ventral views. *Abbreviations*: *adf*, adductor fossa; *ang*, groove for contact with angular; *ap*, articular process; *ds*, dentary suture; *gf*, glenoid fossa; *lfg*, lateral flange of glenoid; *mfg*, medial flange of glenoid; *rp*, process on rostral edge of medial flange of glenoid; *saf*, surangular foramen. Scale bar equals 10 cm.

The surangular contacts the caudal end of the dentary along a nearly vertical suture (Fig. 6A, B). There is no indication of an external mandibular fenestra between the dentary and the surangular. From the apex of the dentary contact, the dorsal margin of the surangular slopes caudoventrally towards the glenoid fossa in which the ventral condyle of the quadrate sat. The glenoid consists of two flanges, one projecting laterally and the other medially, which together form a concave surface (Fig. 6A–E). A small surangular foramen is present on the lateral surface of the surangular rostroventral to the lateral flange of the glenoid (Fig. 6A, C), as in *Mantellisaurus* [30], *Ouranosaurus* [26], *Altirhinus* [32], *Jinzhousaurus* [45], *Equijubus* [33], *Probactrosaurus gobiensis* [13], and *Protohadros* [10], as well as in many non-hadrosauroid iguanodonts, including *Iguanodon bernissartensis* [29], *Fukuisaurus* [27], *Lanzhousaurus* [47], *Theiophytalia* [3], and *Hippodraco* [7]. Caudal to the glenoid fossa, the dorsal margin of the surangular sweeps caudodorsally to form the mediolaterally-compressed articular process (Fig. 6A–F).

The medial surface of the surangular is gently concave to form the caudal portion of the adductor fossa (Fig. 6B, D). The adductor fossa narrows caudally until it becomes only a narrow groove ventral to the medial flange of the glenoid; this groove then becomes broader and shallower on the medial surface of the articular process caudal to the medial flange. There is a small rounded process that projects rostrally from the rostral edge of the medial flange of the glenoid (Fig. 6D); this process likely served as a buttress for the ventral condyle of the quadrate [10]. A deep groove extends horizontally along the ventromedial margin of the surangular, forming the contact surface for the angular (Fig. 6D, F). The ventromedial rather than medial position of this groove indicates that the angular was probably visible in lateral view.

### Premaxilla

**Figure 7:**
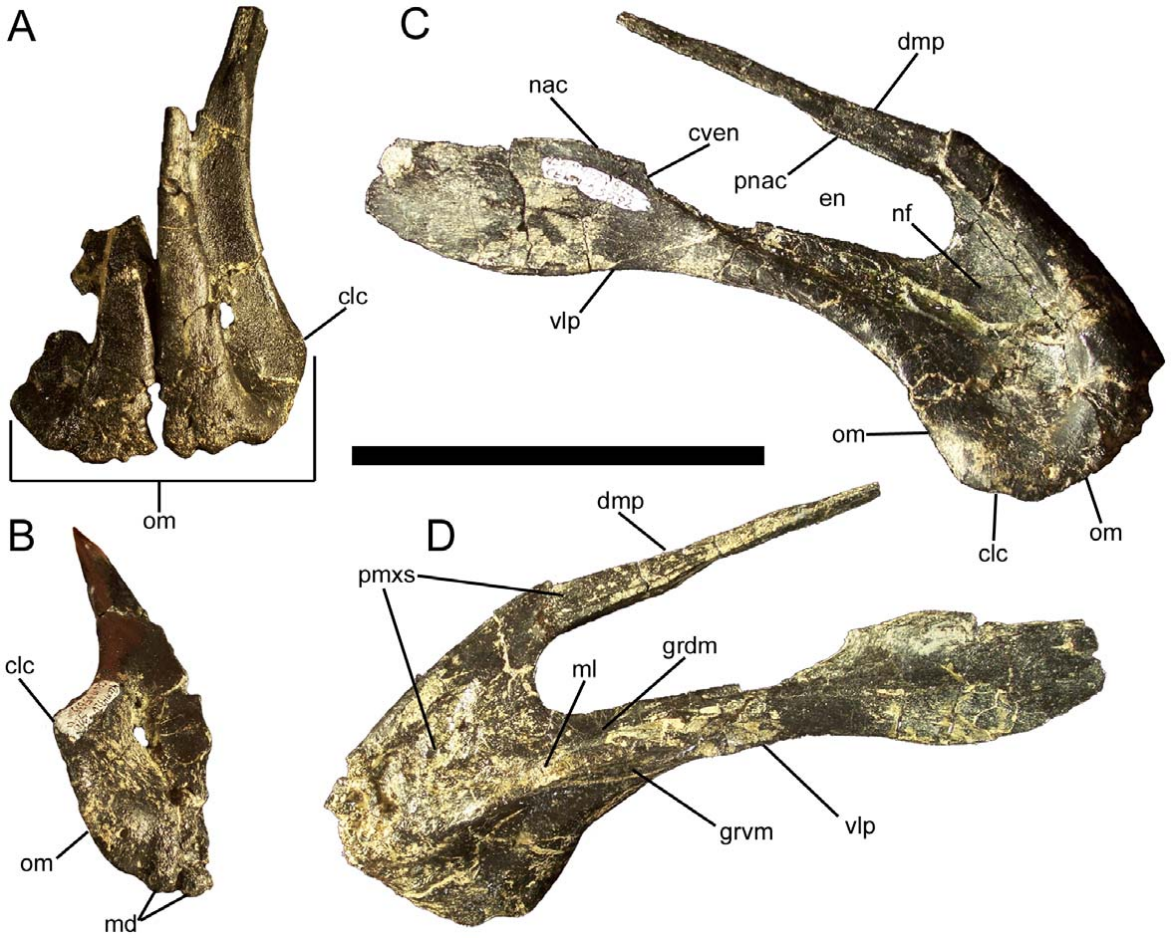
Premaxillae of *Eolambia*. (A) Right (CEUM 35627 [Eo2]) and left (CEUM 35635 [Eo2]) premaxillae in dorsal view. These two premaxillae are disarticulated, but are compatible in size and possibly belong to the same individual, or at least to individuals of similar size. (B) left premaxilla CEUM 35635 (Eo2) in ventral view. Right premaxilla CEUM 35592 (Eo2) in (C) lateral and (D) medial views. *Abbreviations*: *clc*, caudolateral corner of oral margin; *cven*, caudoventral margin of external naris; *dmp*, dorsomedial process; *en*, external naris; *grdm*, groove for rostrodorsal process of right maxilla; *grvm*, groove for rostroventral process of right maxilla; *md*, marginal denticles; *ml*, medial ledge; *nac*, nasal contact; *nf*, narial fossa; *om*, oral margin; *pmxs*, interpremaxillary sutural surface for left premaxilla; *pnac*, contact for premaxillary process of right nasal; *vlp*, ventrolateral process. Scale bar equals 10 cm.

The rostral portion of the premaxilla expands mediolaterally to form a broad edentulous oral margin (Fig. 7A, B). The rostrodorsal surface of the oral margin is highly rugose and pierced by several neurovascular foramina (Fig. 7A). The ventral surface of the oral margin also bears several large foramina, as well as two rostrocaudally-elongate denticles on each premaxilla (Fig. 7B), as in *Mantellisaurus* (NHMUK R5764), *Ouranosaurus* (cast of MNHN GDF 300), and *Protohadros* (SMU 74582), as well as in the non-hadrosauroid iguanodont *Dakotadon* (SDSM 8656). Lateral to the denticles, the oral margin curves caudoventrally and laterally until it forms a rounded corner in lateral view (Fig. 7C). Caudal to this corner, the oral margin sweeps caudodorsally and medially towards the ventrolateral process of the premaxilla (Fig. 7C). The oral margin of the premaxilla is thickened and rugose but lacks the everted lateral rim present in hadrosaurids [48]. Dorsal to the oral margin, the lateral surface of the premaxilla becomes thinner and gently concave to form a shallow depression, the rostral portion of the narial fossa (Fig. 7C). Caudal to the oral margin, the ventral surface of the premaxilla is also gently concave; this slight concavity narrows caudally to form a shallow groove on the ventral surface of the ventrolateral process (Fig. 7D). This groove is the contact surface for the rostroventral process of the maxilla (see below). Although all of the premaxillae and maxillae from the Eo2 bonebed are disarticulated, given the position of the groove on the ventrolateral process of the premaxilla and the morphology of the maxilla, it is clear that the oral margin of the premaxilla projected farther ventrally than the ventral margin of the maxilla. Dorsal to the groove from the rostroventral process of the maxilla, there is a sharp ledge with another shallow groove dorsal to it (Fig. 7D); this groove would have received the rostrodorsal process of the maxilla (see below), with the ledge fitting between the rostrodorsal and rostroventral processes of the maxilla.

Caudal to the transversely expanded oral margin, the premaxilla is divided into a ventrolateral and a dorsomedial process. Both processes project caudodorsally in lateral and medial views and are roughly parallel (Fig. 7C, D), forming the dorsal and ventral rims of the external naris. The ventrolateral process is of approximately uniform dorsoventral depth for much of its length, but abruptly expands dorsoventrally near its caudal end (Fig. 7C, D), as in *Ouranosaurus* [26], *Altirhinus* [32], *Jinzhousaurus* [45], *Probactrosaurus gobiensis* [13], *Protohadros* [10], and *Tethyshadros* [37]. This sudden dorsoventral expansion marks the caudoventral rim of the external naris; the dorsal margin of the dorsoventral expansion would have contacted the ventral margin of the nasal. Because the caudal extremity of the ventrolateral process is missing on the most complete available premaxilla (CEUM 35592) and the lacrimal and prefrontal of *Eolambia* are unknown, it cannot be determined whether the ventrolateral process of the premaxilla contacted the lacrimal and prefrontal.

The medial surface of the base of the dorsomedial process is highly rugose, forming the surface for the interpremaxillary suture (Fig. 7D). This sutural surface extends onto the medial surface of the dorsomedial process itself. There is a slight groove on the ventrolateral surface of the dorsomedial process to receive the premaxillary process of the nasal (Fig. 7C). The dorsomedial process gradually tapers to a point along its length. The external naris is elliptical in lateral and medial views (Fig. 7C, D), with the long axis oriented rostroventral to caudodorsal. Given the positions of the grooves on the premaxilla for the rostrodorsal and rostroventral processes of the maxilla, the external naris extends caudally dorsal to the maxillary tooth row.

### Nasal

**Figure 8:**
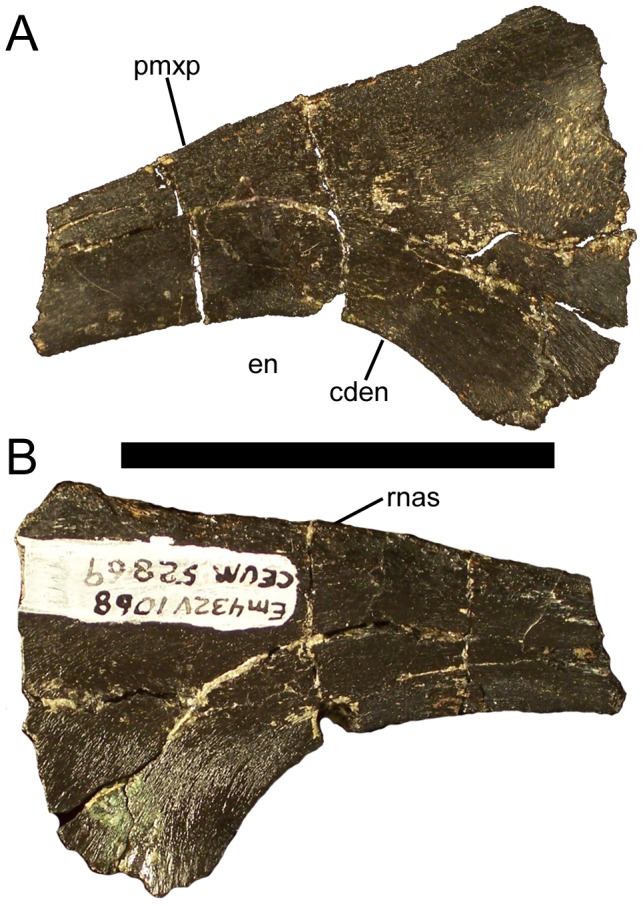
Nasal of *Eolambia*. Left nasal CEUM 52869 (Eo2) in (A) lateral and (B) medial views. *Abbreviations*: *cden*, caudodorsal margin of external naris; *en*, external naris; *pmxp*, premaxillary process; *rnas*, sutural surface for right nasal. Scale bar equals 5 cm.

Two partial nasals are known, a possible right from paratype CEUM 5212 and a left from the Eo2 bonebed (CEUM 52869). Both preserve only the region of the caudodorsal rim of the external naris; CEUM 52869 is the better preserved of the two. The premaxillary process of the nasal projects rostrally to form the caudodorsal rim of the external naris (Fig. 8A). The lateral surface of the nasal is gently convex, while the medial side is slightly concave. The dorsomedial edge of CEUM 52869 bears a sutural surface for the corresponding edge of the right nasal (Fig. 8B). The ventral contact for the maxilla is broken in both nasals.

### Maxilla

**Figure 9:**
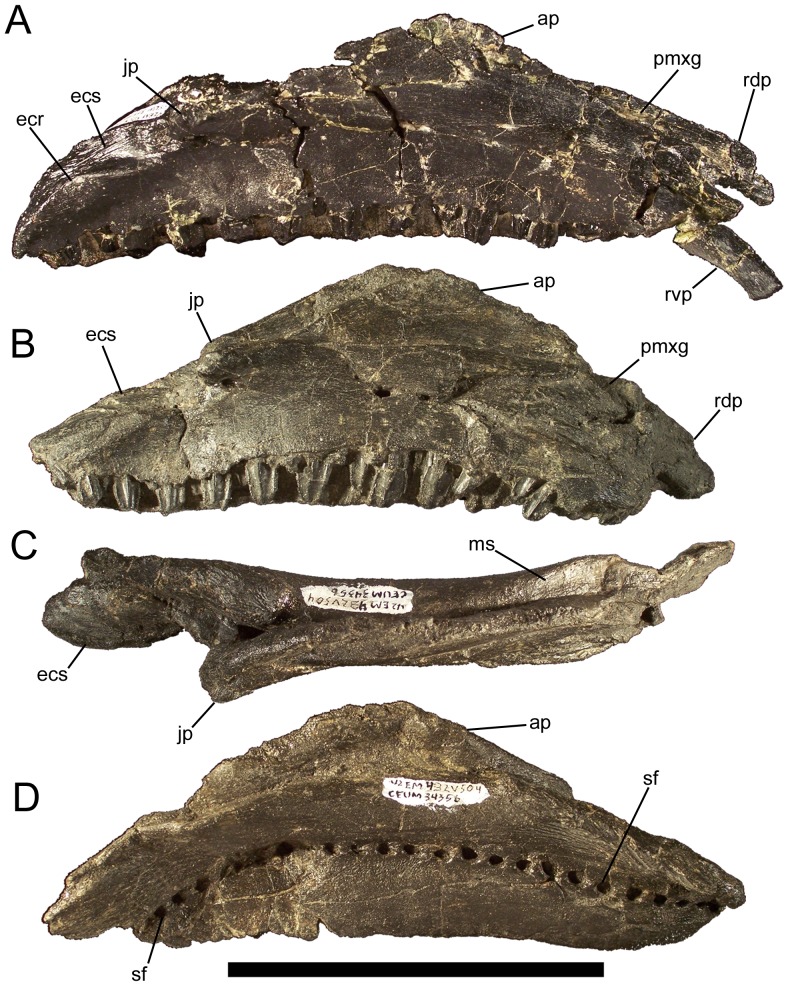
Maxillae of *Eolambia*. (A) Right maxilla CEUM 35492 (Eo2) in lateral view. Right maxilla CEUM 34356 (Eo2) in (B) lateral, (C) dorsal, and (D) medial views. *Abbreviations*: *ap*, ascending process; *ecr*, ectopterygoid ridge; *ecs*, ectopterygoid shelf; *jp*, jugal process; *ms*, medial shelf; *pmxg*, premaxillary groove; *rdp*, rostrodorsal process; *rvp*, rostroventral process; *sf*, special foramina. Scale bar equals 10 cm.

**Figure 10:**
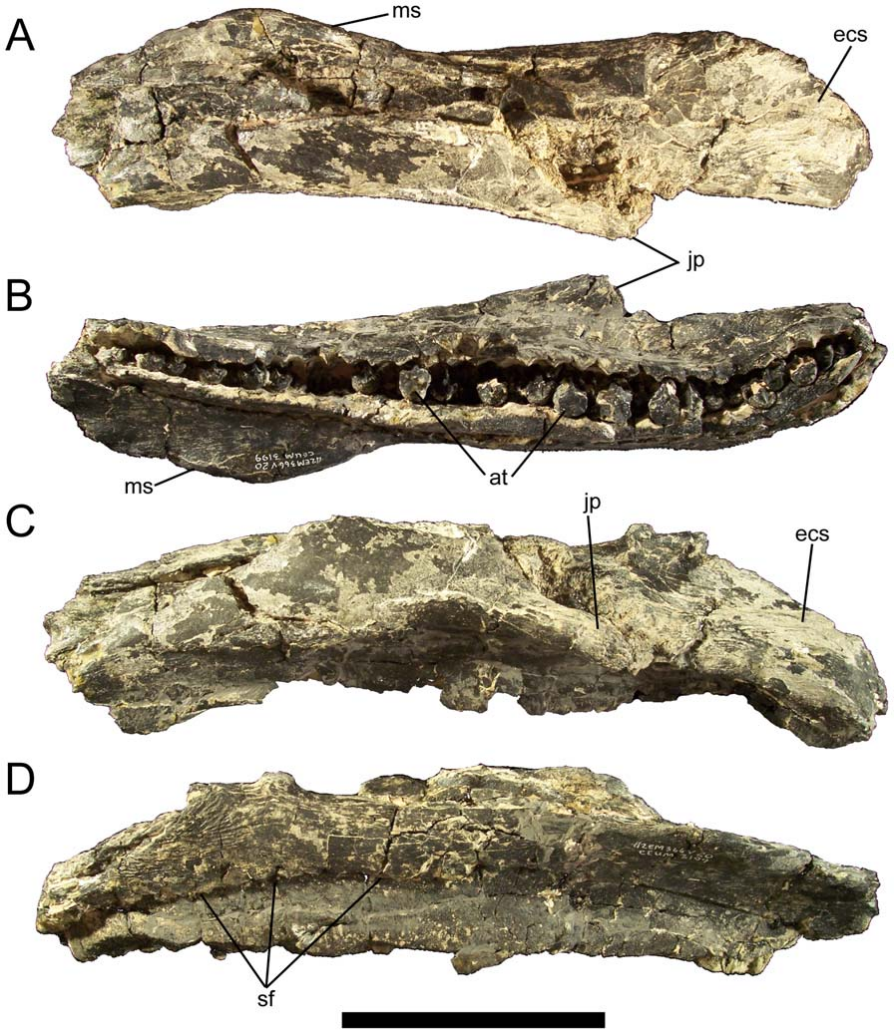
Maxilla of *Eolambia*. Left maxilla of CEUM 9758 (holotype) in (A) dorsal, (B) ventral, (C) lateral, and (D) medial views. *Abbreviations*: *at*, active tooth; *ecs*, ectopterygoid shelf; *jp*, jugal process; *ms*, medial shelf; *sf*, special foramina. Scale bar equals 10 cm.

The rostral end of the maxilla is divided into two finger-like processes, the rostroventral and rostrodorsal processes, as in *Mantellisaurus* [30], *Altirhinus* [32], *Protohadros* [10], *Shuangmiaosaurus* [34], *Bactrosaurus* [38], and *Gilmoreosaurus* [40]. The rostroventral process arises immediately rostral to the first alveolus and curves rostroventrally (Fig. 9A). The rostrodorsal process arises dorsal to the rostroventral process and also curves rostroventrally (Fig. 9A, B). Caudodorsal to the rostrodorsal process is a shallow groove to receive the ventrolateral process of the premaxilla (Fig. 9A, B); this groove narrows caudally and continues medial to the base of the ascending process of the maxilla (Fig. 9C). Medial to this groove is a rounded shelf that projects dorsomedially (Figs. 9C; 10A, B).

The ascending process arises from the lateral surface of the maxilla and projects dorsally (Fig. 9A, B). The ascending process is rostrocaudally elongate and triangular in lateral and medial views (Fig. 9A, B, D). There is no depression or embayment along the caudal margin of the ascending process that would indicate the presence of an external antorbital fossa or fenestra, as in *Altirhinus* [32], *Jinzhousaurus* [45], *Equijubus* [33], *Xuwulong* [35], *Protohadros* [10], *Jeyawati* [11], *Shuangmiaosaurus* [34], *Bactrosaurus* [38], *Gilmoreosaurus* [40], and *Levnesovia* [36]. The jugal process arises from the caudal end of the base of the ascending process and projects caudolaterally (Figs. 9A–C; 10A–C). The jugal process is a blunt, finger-like prong that fits into a recess on the medial surface of the maxillary process of the jugal (see below), as in *Iguanodon bernissartensis* [29], *Mantellisaurus* [30], *Ouranosaurus* [26], *Altirhinus* [32], *Jinzhousaurus* [45], *Probactrosaurus gobiensis* [13], *Protohadros* [10], and *Jeyawati* [11]. Medial to the jugal process is a deep trough that extends caudoventrally and then flattens out to form a broad shelf on which the ectopterygoid would sit (Figs. 9C; 10A). The ectopterygoid shelf is bounded laterally by a fine sinuous ectopterygoid ridge (Fig. 9A). The dorsal surface of the ectopterygoid shelf slopes dorsomedially from the ectopterygoid ridge (Figs. 9A, B; 10C).

In dorsal view, the maxilla is straight for almost its entire length (Figs. 9C; 10A). The maxilla is gently concave along its ventral margin in lateral view (Figs. 9A, B; 10C). In ventral view, the maxillary tooth row is bowed medially, with the rostral and caudal ends curving laterally (Fig. 10B). The alveoli are shallow, parallel-sided furrows (Fig. 10B). The left maxilla of holotype CEUM 9758 preservers 33 alveoli; the smaller, juvenile right maxilla CEUM 34356 from the Eo2 bonebed bears 23 alveoli. The lateral surface of the maxilla is pierced by 4–5 irregularly-spaced neurovascular foramina of different sizes (Fig. 9A, B). The medial surface of the maxilla bears numerous closely-spaced ‘special foramina’ arranged in an arc that extends from immediately caudal to the rostrodorsal process to the caudal end of the maxilla (Figs. 9D; 10D).

### Jugal

**Figure 11:**
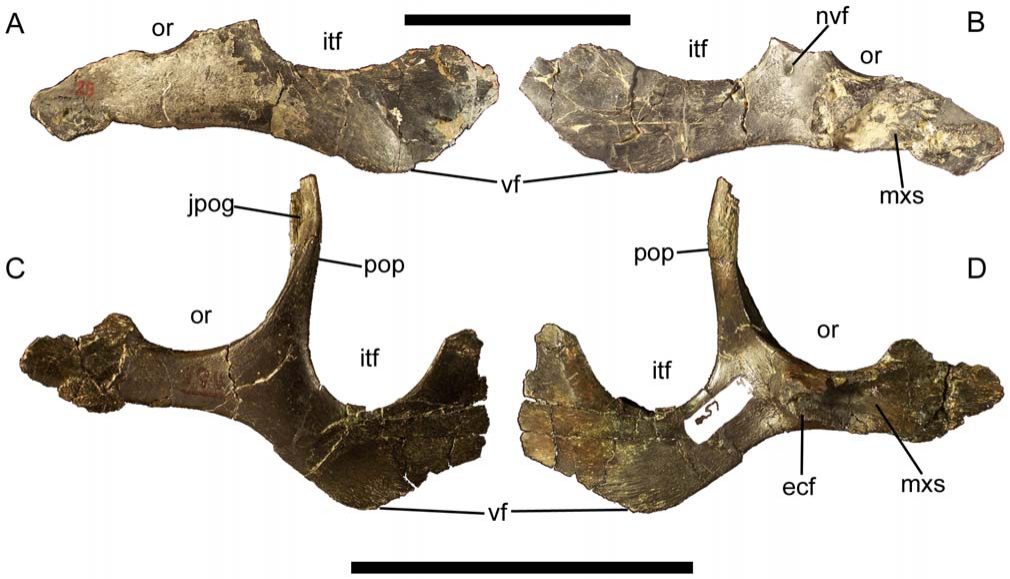
Jugals of *Eolambia*. Left jugal of CEUM 9758 (holotype) in (A) lateral and (B) medial views. Left jugal CEUM 52204 (WS8) in (C) lateral and (D) medial views. *Abbreviations*: *ecf*, ectopterygoid facet; *itf*, infratemporal fenestra; *jpog*, groove for jugal process of postorbital; *mxs*, slot for jugal process of maxilla; *nvf*, neurovascular foramen; *or*, orbit; *pop*, postorbital process; *vf*, ventral flange. Scale bars equal 10 cm.

The rostral end of the maxillary process of the jugal is dorsoventrally expanded (Fig. 11), as in *Ouranosaurus* [26], *Protohadros* [10], *Bactrosaurus* [38], *Gilmoreosaurus* [40], *Levnesovia* [36], *Tanius* [44], and *Tethyshadros* [37]. The lateral surface of the maxillary process is flat and featureless (Fig. 11A, C), whereas the medial surface bears a rostrocaudally elongate slot into which the jugal process of the maxilla would fit (Fig. 11B, D). Caudal to this slot is a circular facet for contact with the ectopterygoid (Fig. 11D). Caudal to the expanded rostral end, the dorsal margin of the maxillary process is gently concave to form the ventral margin of the orbit (Fig. 11). The postorbital process arises at the caudal end of this concavity and curves rostrodorsally (Fig. 11C, D). There is a large neurovascular foramen on the medial surface of the base of the postorbital process (Fig. 11B). The postorbital process exhibits a shallow groove on its rostrolateral surface, in which the jugal process of the postorbital would fit (Fig. 11C). Caudal to the base of the postorbital process of the jugal is another, more acute concave margin, which forms the entire ventral margin of the infratemporal fenestra (Fig. 11). The caudal end of the jugal is dorsoventrally expanded at its contact with the quadratojugal. The ventral margin of the jugal is angular, with a prominent flange ventral to the infratemporal fenestra (Fig. 11), as in *Ouranosaurus* [26], *Altirhinus* [32], *Jinzhousaurus* [45], *Equijubus* [33], *Xuwulong* [35], *Probactrosaurus gobiensis* [13], *Protohadros* [10], and *Bactrosaurus* [38].

### Postorbital

**Figure 12:**
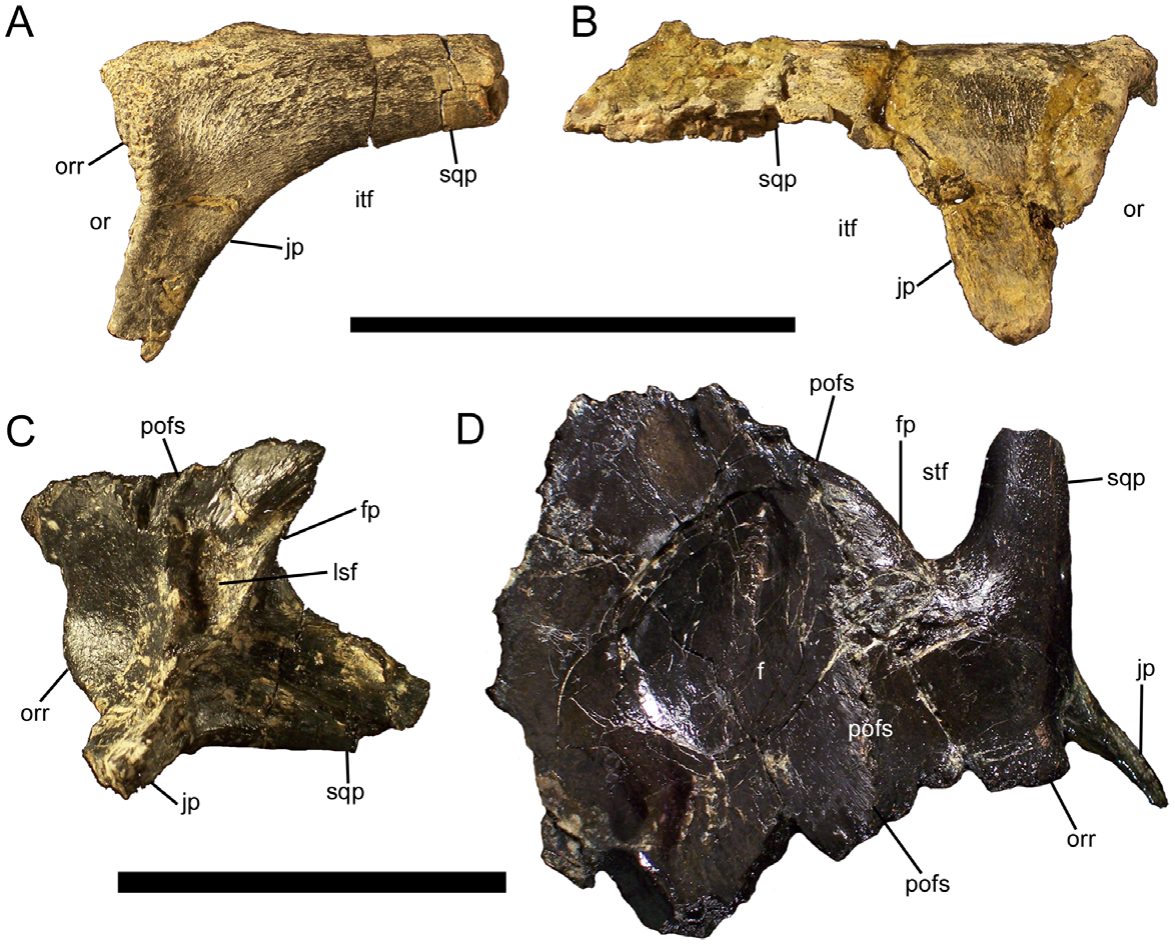
Postorbitals of *Eolambia*. (A) Left postorbital of CEUM 9758 (holotype) in lateral view. (B) Right postorbital of CEUM 5212 (paratype) in lateral view. (C) Right postorbital CEUM 35748 (Eo2) in ventromedial view. (D) Articulated left postorbital and frontal CEUM 52091 (WS8) in dorsal view. *Abbreviations*: *f*, frontal; *fp*, frontal process; *itf*, infratemporal fenestra; *jp*, jugal process; *lsf*, facet for contact with laterosphenoid; *or*, orbit; *orr*, orbital rim; *pofs*, postorbital-frontal suture; *sqp*, squamosal process; *stf*, supratemporal fenestra. Scale bar in A and B equals 10 cm; scale bar in C and D equals 5 cm.

The postorbital comprises the caudodorsal corner of the orbital rim. The orbital rim of the postorbital is highly rugose, with a series of bumps and grooves (Fig. 12A) similar to those of other basal iguanodonts, such as *Protohadros byrdi* (SMU 74582). In contrast, the lateral surface of the postorbital is quite smooth (Fig. 12A, B). Three processes arise from the body of the postorbital. The jugal process projects rostroventrally to meet the postorbital process of the jugal, with which it forms the caudal margin of the orbit and the rostral margin of the infratemporal fenestra (Fig. 12A, B). The straight squamosal process projects caudally to overlap the postorbital process of the squamosal (see below), with which it forms the dorsal margin of the infratemporal fenestra and the lateral margin of the supratemporal fenestra (Fig. 12). In all available postorbitals of *Eolambia*, the caudal end of the squamosal process is broken; it is thus unknown whether the caudal of the process was tapered, rounded, or bifurcated.

The frontal process of the postorbital projects medially and is not a prong like the jugal and squamosal processes, but instead abruptly widens rostrocaudally along the postorbital-frontal suture (Fig. 12C, D). The sutural surface of the frontal process terminates while in contact with the frontal (Fig. 12D), i.e., it does not extend rostrolaterally beyond the frontal, indicating that there was no contact between the prefrontal and the postorbital and that the frontal participated in the orbital rim. The postorbital-frontal suture is oriented rostrolateral to caudomedial and appears as a series of interlocking bumps in dorsal view (Fig. 12D). The frontal process of the postorbital forms the rostromedial margin of the supratemporal fenestra (Fig. 12D). On the medial surface of the postorbital ventral to the frontal process is a small facet, which is the contact surface for the laterosphenoid (see below) (Fig. 12C). The laterosphenoid facet is defined dorsally by the frontal process, rostrally and caudally by two fine ridges, and ventrally by the convergence of those two ridges (Fig. 12C).

### Quadrate

**Figure 13:**
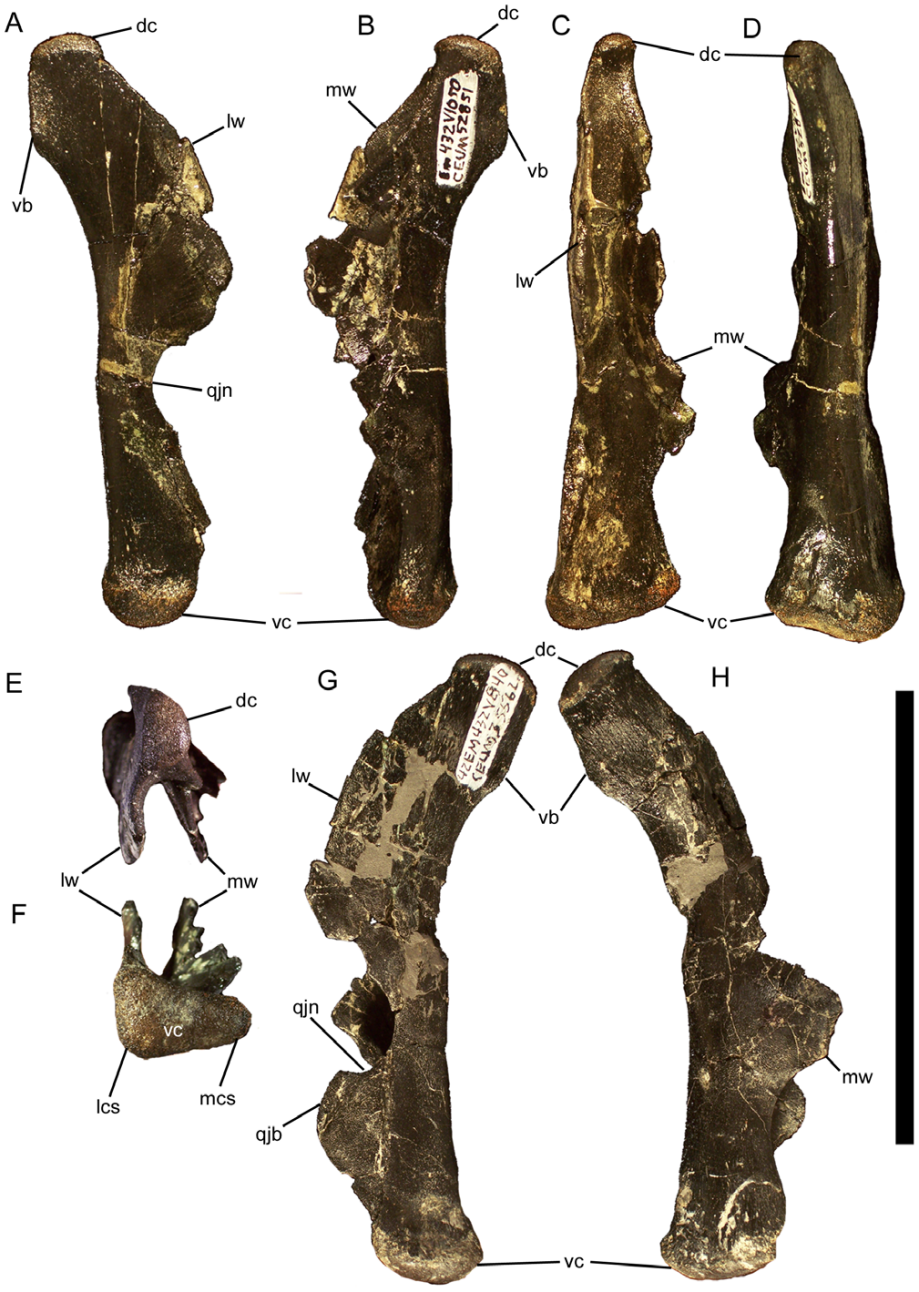
Quadrates of *Eolambia*. Right quadrate CEUM 52851 (Eo2) in (A) lateral, (B) medial, (C) rostral, (D) caudal, (E) dorsal, and (F) ventral views. Left quadrate CEUM 35562 (Eo2) in (G) lateral and (H) medial views. *Abbreviations*: *dc*, dorsal condyle; *lcs*, lateral condylar surface; *lw*, lateral wing; *mcs*, medial condylar surface; *mw*, medial wing; *qjb*, quadratojugal buttress; *qjn*, quadratojugal notch; *vb*, vertical buttress; *vc*, ventral condyle. Scale bar equals 10 cm.

The quadrate is straight for most of its length in lateral and medial views, curving caudally only near the dorsal condyle (Fig. 13A, B, G, H). The quadrate is also straight in rostral and caudal views (Fig. 13C, D). The shaft of the quadrate is divided into a lateral and a medial wing (Fig. 13). The medial wing projects rostromedially (Fig. 13C–F) and would contact the pterygoid. The medial wing is broken in all known quadrates of *Eolambia*, rendering its full size and morphology unknown. The lateral wing projects rostrally (Fig. 13C, E, F) and would contact the quadratojugal. The lateral wing bears a prominent semicircular quadratojugal notch in its rostral margin (Fig. 13A, G). There is a thickened buttress immediately ventral to the notch (Fig. 13G), the rostral face of which is flat. Dorsal to the notch is another, slightly less prominent buttress. These morphologies associated with the notch in the lateral wing of the quadrate suggest that the quadratojugal did not overlap or fill the notch, but rather was in contact with the buttresses dorsal and ventral to the notch. Therefore, a paraquadrate foramen likely was present, though it is impossible to be sure without a quadratojugal of *Eolambia*.

The dorsal condyle of the quadrate would rest in the glenoid fossa on the ventral surface of the squamosal (see below). The dorsal condyle is D-shaped in dorsal view, with the straight edge of the ‘D’ facing laterally (Fig. 13 E), as in *Mantellisaurus* (NHMUK R5764), *Jeyawati* [11], *Bactrosaurus* [38], *Gilmoreosaurus* [40] and *Telmatosaurus* (NHMUK R3386). There is a sharp vertical buttress on the caudal surface of the quadrate immediately ventral to the dorsal condyle (Fig. 13A, B, G, H). The ventral condyle of the quadrate would rest in the glenoid fossa on the dorsal surface of the surangular (see above). The ventral condyle is mediolaterally broad (Fig. 13C, D, F); the lateral condylar surface is rostrocaudally longer than the medial condylar surface (Fig. 13F).

### Squamosal

**Figure 14:**
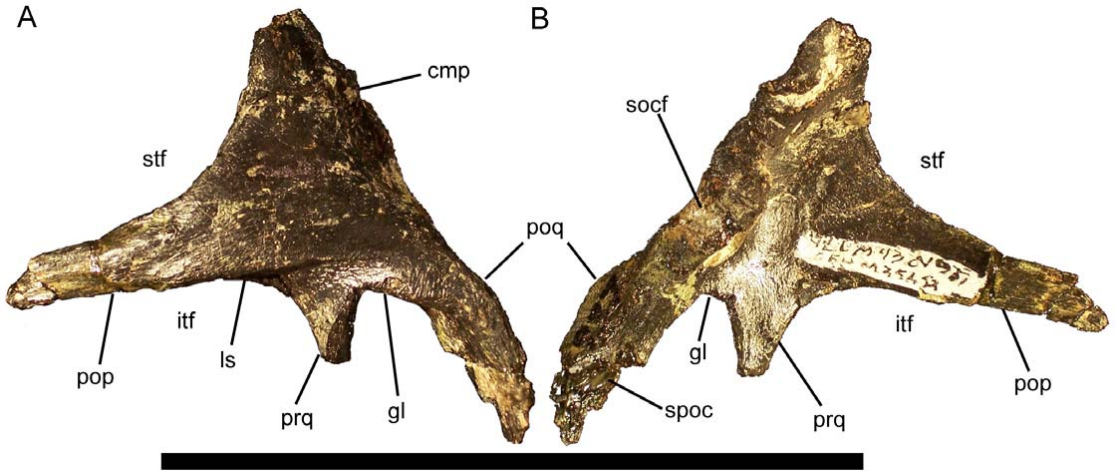
Squamosal of *Eolambia*. Left squamosal CEUM 35452 (Eo2) in (A) dorsolateral and (B) ventromedial views. *Abbreviations*: *cmp*, caudomedial process; *gl*, glenoid fossa; *itf*, infratemporal fenestra; *ls*, lateral shelf; *pop*, postorbital process; *poq*, postquadrate process; *prq*, prequadrate process; *socf*, supraoccipital facet; *spoc*, suture for paroccipital process; *stf*, supratemporal fenestra. Scale bar equals 10 cm.

The squamosal is a complex bone consisting of four processes. The postorbital process projects rostrally and tapers towards its rostral end (Fig. 14). The postorbital process of the squamosal and the squamosal process of the postorbital join to form the dorsal margin of the infratemporal fenestra and the lateral margin of the supratemporal fenestra. A broad lateral shelf originates on the lateral surface of the postorbital process and extends along the lateral surface of the squamosal to a point dorsal to the prequadrate process (Fig. 14A). This shelf defines the dorsal margin of a depression ventral to it that forms the origin site of *M. adductor mandibulae externus superficialis* [49]. Dorsal to the lateral shelf, the surface of the postorbital process is gently convex. The glenoid fossa, in which the dorsal condyle of the quadrate would fit, is a deep depression on the ventral aspect of the squamosal. The glenoid fossa is bounded rostrally by the prequadrate process, which projects ventrally, and caudally by the postquadrate process, which curves caudoventrally (Fig. 14). The medial surface of the postquadrate process bears a sutural surface that would contact the rostrolateral surface of the paroccipital process (Fig. 14B).

The caudomedial process of the squamosal curves rostromedially to contact the supraoccipital and the parietal, and to form the caudal margin of the supratemporal fenestra. Dorsomedial to the glenoid, the caudal margin of the caudomedial process is thickened to form a rugose concave facet that would contact the supraoccipital (Fig. 14B). The locations of the contact surfaces for the right and left squamosals on the parietal (see below) indicate that only a narrow sliver of the parietal separated the caudomedial processes of the right and left squamosals on the dorsal surface of the skull, as in *Jinzhousaurus* [45], *Jintasaurus* [50], *Probactrosaurus gobiensis* [13], *Bactrosaurus* [38], *Levnesovia* [36], and *Tanius* [44].

### Neurocranium

Three articulated skull roofs and braincases are present in the CEUM *Eolambia* material: the partial neurocranium of holotype CEUM 9758 and two nearly complete examples from the Eo2 bonebed (CEUM 35475 and 74552). The neurocranium of CEUM 9758 is broken into two damaged pieces, one of which includes the left and right frontals, presphenoid, left and right orbitosphenoids, and partial parietal, and another that includes the supraoccipital, left and right fused opisthotics-exoccipitals lacking the paroccipital processes, and part of the basisphenoid. CEUM 35475 includes the articulated basioccipital, fused parasphenoid-basisphenoid, left and right laterosphenoids, left and right prootics, left and right fused opisthotics-exoccipitals, and supraoccipital. CEUM 74552 includes the parietal and probably fragments of the frontals in addition to the same articulated elements as CEUM 35475. In addition to these three specimens, paratype CEUM 5212 includes an articulated complete parietal, supraoccipital, and partial left and right opisthotics-exoccipitals, as well as a separate fragment consisting of the partial articulated basisphenoid and basioccipital. Furthermore, disarticulated frontals, parietals, supraoccipitals, fused opisthotics-exoccipitals, prootics, laterosphenoids, fused parasphenoids-basisphenoids, and basioccipitals are known from the Eo2 and WS8 bonebeds. Thus, every braincase element except the presphenoid and orbitosphenoid is represented by multiple specimens. Together, these articulated and disarticulated examples allow a thorough description of the neurocranium of *Eolambia*.

**Figure 15:**
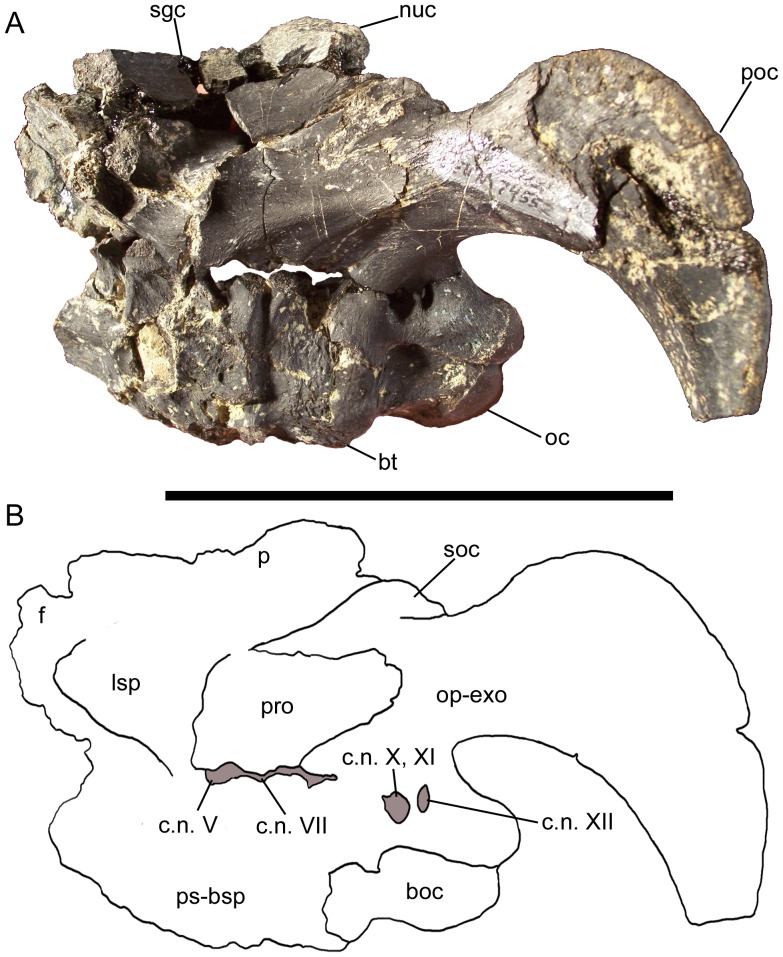
Braincase of *Eolambia*. Braincase CEUM 74552 (Eo2) in (A) left lateral view and (B) traced outline in left lateral view. *Abbreviations*: *boc*, basioccipital; *bt*, basal tuber; *c.n.*, foramina for cranial nerves V, VII, X, XI, and XII; *f*, frontal; *lsp*, laterosphenoid; *nuc*, nuchal crest; *oc*, occipital condyle; *op-exo*, opisthotic-exoccipital; *p*, parietal; *poc*, paroccipital process; *pro*, prootic; *ps-bsp*, parasphenoid-basisphenoid; *sgc*, sagittal crest; *soc*, supraoccipital. Scale bar equals 10 cm.

The known skull roof and braincase elements of *Eolambia* (frontal, parietal, supraoccipital, fused opisthotic-exoccipital, prootic, laterosphenoid, fused parasphenoid-basisphenoid, and basioccipital) are individually described in the following eight sections. Descriptions both refer to disarticulated examples of each element and to the articulated elements of CEUM 9758, 35475, and especially 74552 (Fig. 15) to illustrate spatial and sutural relationships among the components of the neurocranium. The presphenoid and orbitosphenoid are known only from badly damaged examples attached to the frontals of CEUM 9758, and thus are briefly mentioned in the ‘Frontal’ section.

### Frontal

**Figure 16:**
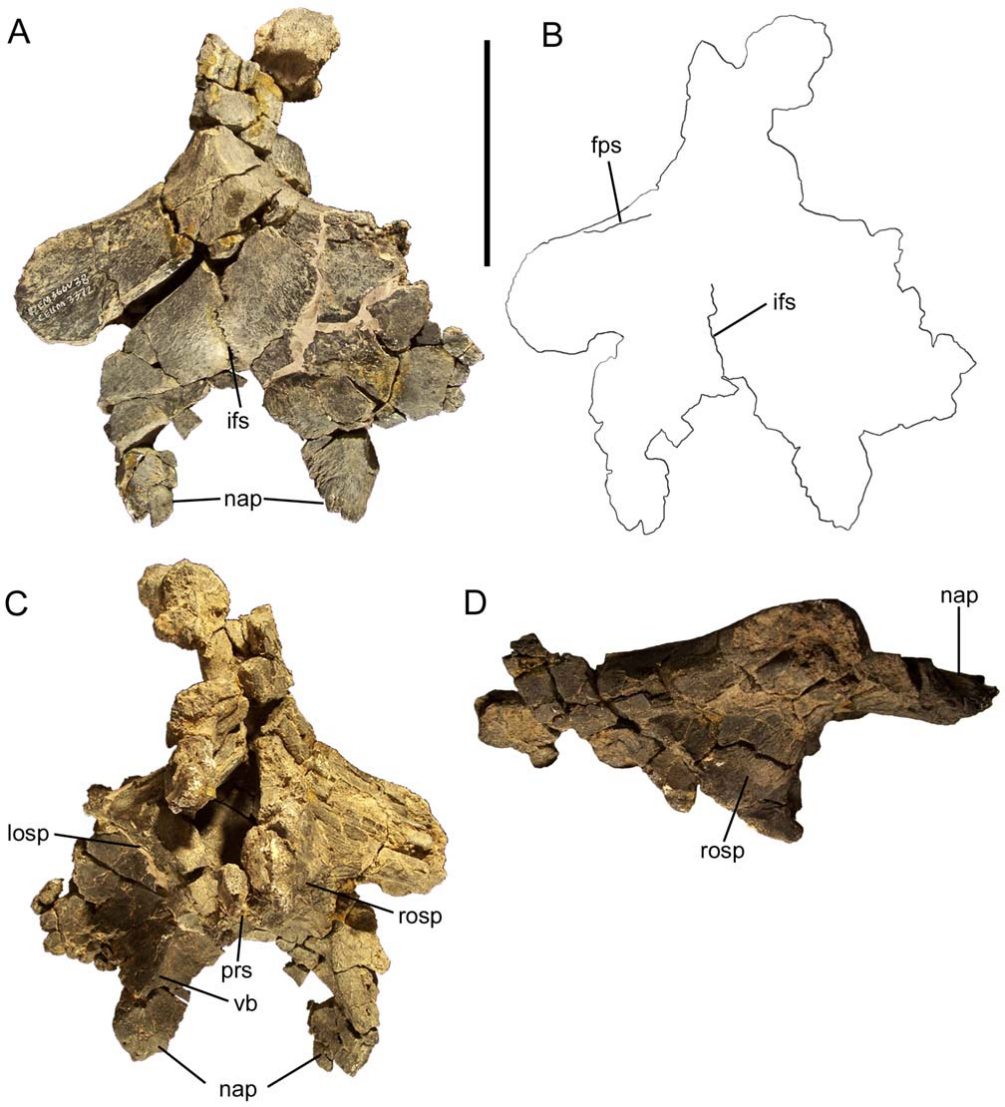
Partial braincase of *Eolambia*. Partial braincase of CEUM 9758 (holotype) in (A) dorsal, (B) tracing in dorsal, (C) ventral, and (D) right lateral views. *Abbreviations*: *fps*, frontal-parietal suture; *ifs*, interfrontal suture; *losp*, left orbitosphenoid; *nap*, nasal process of frontal; *prs*, presphenoid; *rosp*, right orbitosphenoid; *vb*, ventral buttress. Scale bar equals 10 cm.

**Figure 17:**
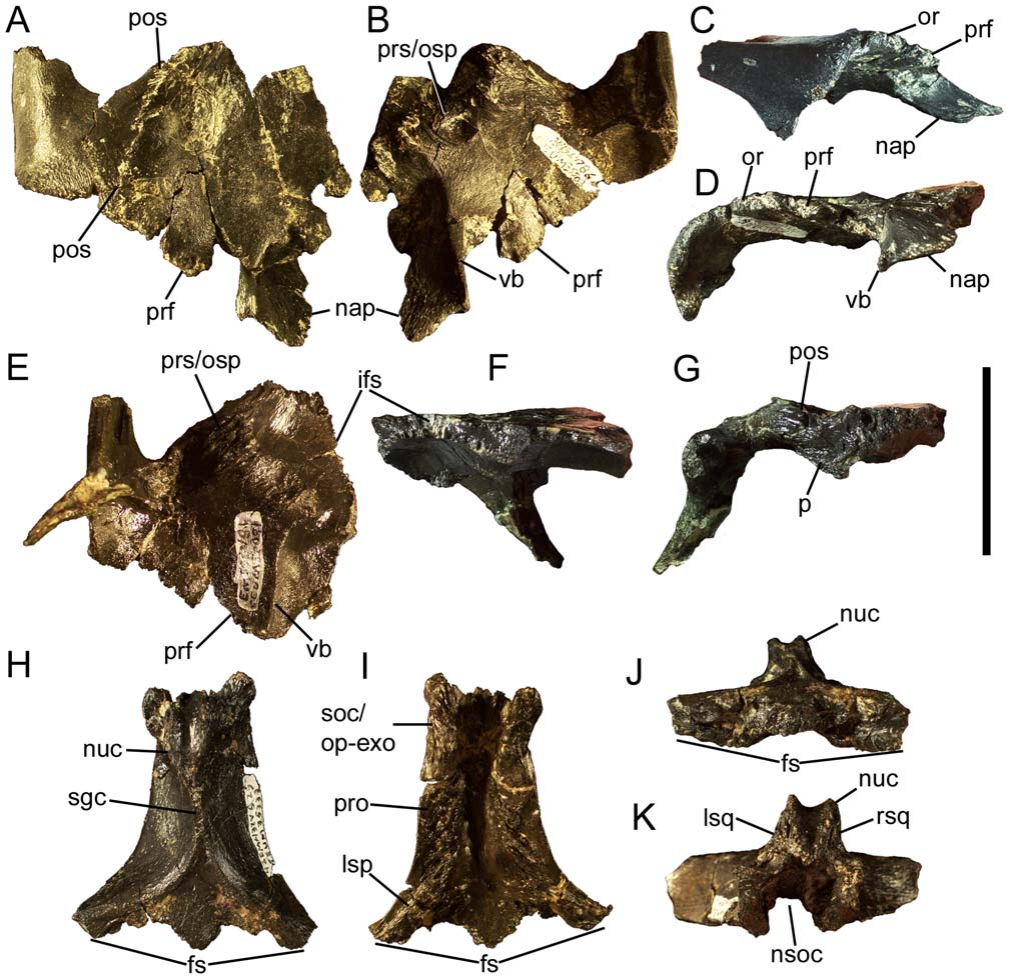
Frontals and parietal of *Eolambia*. Right frontal and postorbital CEUM 35502 (Eo2) in (A) dorsal, (B) ventral, (C) lateral, and (D) rostral views. Left frontal and postorbital CEUM 52091 (WS8) in (E) ventral, (F) medial, and (G) caudal views. Parietal CEUM 35339 (Eo2) in (H) dorsal, (I) ventral, (J) rostral, and (K) caudal views. *Abbreviations*: *fs*, frontal suture; *ifs*, interfrontal suture; *lsp*, suture for laterosphenoid; *lsq*, suture for left squamosal; *nap*, nasal process of frontal; *nsoc*, notch for supraoccipital; *nuc*, nuchal crest; *or*, orbital rim; *p*, parietal suture; *pos*, postorbital suture; *prf*, prefrontal suture; *pro*, suture for prootic; *prs/osp*, suture for presphenoid and orbitosphenoid; *rsq*, suture for right squamosal; *sgc*, sagittal crest; *soc/op-exo*, suture for supraoccipital and opisthotic-exoccipital; *vb*, ventral buttress. Scale bar equals 5 cm.

The dorsal surface of the frontal is flat, lacking any manner of crest or protuberance (Figs. 16A, B; 17A). The rostral margin of the frontal bears a rostroventrally-directed, dorsoventrally compressed, finger-like process that would have been overlain by the caudal end of the nasal (Figs. 16; 17A–D). The ventral surface of this nasal process exhibits a sharply defined, rostrocaudally-directed buttress, which makes the lateral margin of the nasal process dorsoventrally deeper than the medial margin (Figs. 16C; 17B, D, E). The buttress continues caudally onto the ventral surface of the frontal and curves caudomedially, defining the lateral and caudal margins of a roughly semicircular depression on the ventral surface of the frontal.

The ventral surface of the frontal also bears a rostromedial to caudolateral suture to which the presphenoid and orbitosphenoid would attach (Fig. 17B, E). The presphenoid and orbitosphenoids form the rostral and rostrolateral walls of the braincase; they are still attached to the frontals of CEUM 9758, but are too badly damaged to discern details of their anatomy (Fig. 16C, D). It appears that the rostral wall of the braincase slopes caudoventrally from its contact with the frontals and that the lateral surfaces of the orbitosphenoids are flat (Fig. 16D).

Lateral to the aforementioned nasal process, the rostrolateral margin of the frontal bears a sutural surface for the caudomedial margin of the prefrontal (Fig. 17A–E). However, this sutural surface does not extend caudally to meet the contact with the postorbital; instead, a short portion of the lateral margin of the frontal bears a rugose texture similar to that on the orbital rim of the postorbital, suggesting that the frontal participated in the dorsal margin of the orbit (Fig. 17C, D) (see also “Postorbital” section above). The caudolateral margin of the frontal contacts the postorbital along a rostrolateral to caudomedial suture, as previously described in the ‘Postorbital’ section (Fig. 17A).

The right or left frontal meets its counterpart along a midline suture (Figs. 16A, B; 17E, F). This suture reaches its dorsoventrally thickest point approximately half-way along its length and dorsally bowed along its caudal half (Fig. 17F). The caudal margin of the frontal bears a robust sutural contact for the parietal. This suture is dorsoventrally thickest at its lateral-most point, caudoventral to the contact between the frontal and the postorbital (Fig. 17G).

### Parietal

The rostral margin of the parietal is laterally expanded and bears a suture for the right and left frontals (Figs. 16B; 17H–J). This suture is not straight, but rather exhibits a midline tab that would have been locked between the caudal margins of the frontals (Fig. 17H, I). The dorsal surface of the parietal bears two rounded ridges that extend parallel to the frontal suture until curving caudally and converging to form the sagittal crest (Fig. 17H). The sagittal crest slopes caudodorsally towards the nuchal crest at the caudal end of the parietal (Figs. 15A; 17H, J, K). The nuchal crest is divided into left and right pinnacles by a midline furrow and bears sutures on its lateral surfaces to which the ends of the caudomedial processes of the right and left squamosals would have attached (Fig. 17K). Ventral to the nuchal crest is a deep notch to receive the parietal process of the supraoccipital (Fig. 17K) (see below).

The lateral surfaces of the parietal slope ventrolaterally away from the sagittal crest to form the dorsolateral walls of the braincase (Figs. 15; 17H). The ventral margins of the lateral surfaces of the parietal exhibit extensive sutures for the bones of the lateral wall of the braincase. The rostral-most suture would meet the dorsal margin of the laterosphenoid; this suture curves laterally to follow the morphology of the laterosphenoid, which also curves laterally (see below), and the lateral expansion of the parietal along the frontal suture (Fig. 17I). Caudal to the laterosphenoid suture, the sutural surface becomes rostrocaudally straight and would contact the dorsal surface of the prootic, and finally the dorsal surfaces of the supraoccipital and fused opisthotic-exoccipital complex at its caudal end (Fig. 17I).

### Supraoccipital

**Figure 18:**
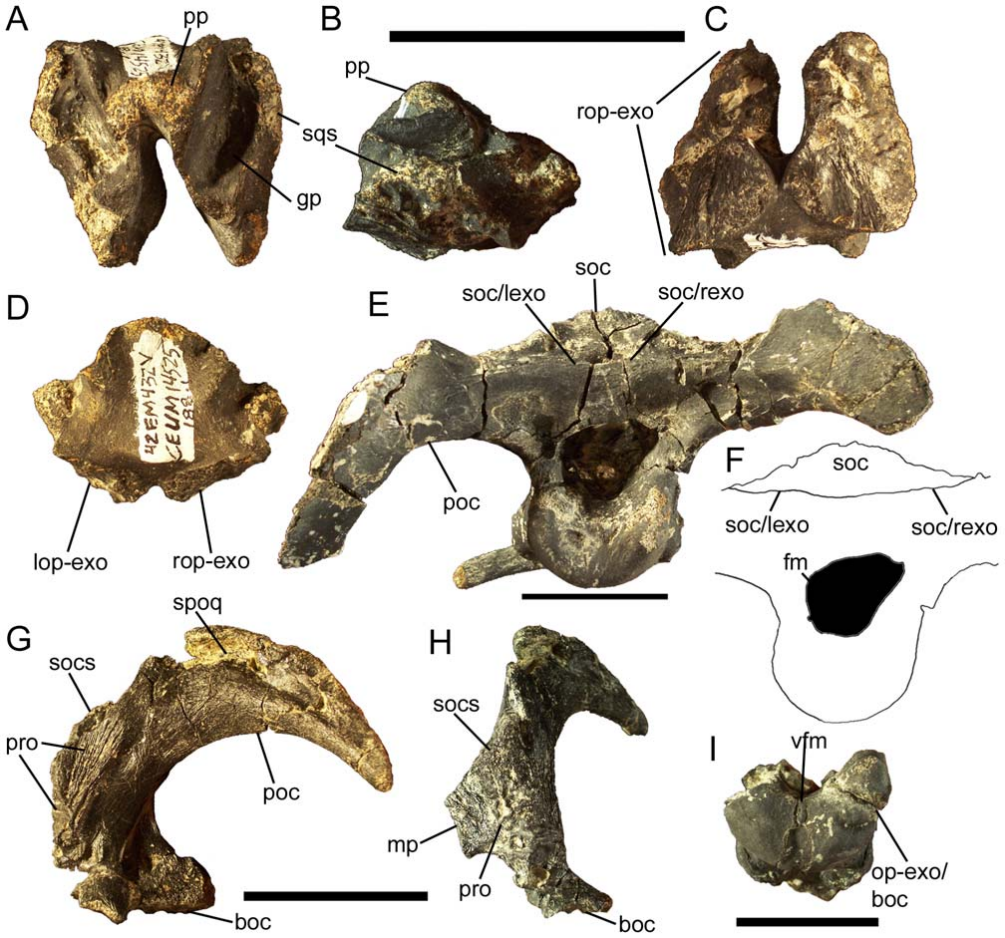
Supraoccipital and opisthotic-exoccipital of *Eolambia*. Supraoccipital CEUM 14525 (Eo2) in (A) dorsal, (B) right lateral, (C) ventral, and (D) caudal views. Braincase CEUM 35475 (Eo2) in (E) caudal view and (F) tracing in caudal view without paroccipital processes. Left opisthotic-exoccipital CEUM 35622 (Eo2) in (G) lateral and (H) rostral views. Ventral portion of braincase CEUM 74552 (Eo2) in (I) caudal view. *Abbreviations*: *boc*, suture for basioccipital; *fm*, foramen magnum; *gp*, groove for parietal; *lop-exo*, suture for left opisthotic-exoccipital; *mp*, medial process; *op-exo/boc*, contact between right opisthotic-exoccipital and basioccipital; *poc*, paroccipital process; *pp*, parietal process; *pro*, suture for prootic; *rop-exo*, suture for right opisthotic-exoccipital; *soc*, supraoccipital; *soc/lexo*, contact between supraoccipital and left exoccipital; *soc/rexo*, suture between supraoccipital and right exoccipital; *socs*, suture for supraoccipital; *spoq*, suture for postquadrate process of squamosal; *sqs*, squamosal suture; *vfm*, ventral margin of foramen magnum. Scale bars equal 5 cm.

The dorsal surface of the supraoccipital bears an intricate pattern of processes, grooves, and sutures, reflecting the complex relationships of the supraoccipital to other bones of the skull. The rounded parietal process arises from the caudodorsal margin of the supraoccipital to fit into the aforementioned notch on the ventral surface of the parietal (Fig. 18A, B). Lateral to the parietal process, on either side, are two deep grooves with rugose floors that would receive the ventral margins of the lateral walls of the parietal (Fig. 18A). Lateral to these groove are rugose sutures that would meet the supraoccipital facets on the caudomedial processes of the left and right squamosals (Fig. 18A, B).

The ventral surface of the supraoccipital bears extensive sutures for the left and right opisthotic-exoccipital complexes (Fig. 18C). The left and right sutures nearly touch at their caudal ends, indicating that the exoccipitals excluded the supraoccipital from the foramen magnum (Fig. 18C, D); this is confirmed by articulated braincases (Fig. 18E, F). The opisthotic-exoccipital sutures sweep dorsolaterally and converge with the squamosal sutures on the lateral surfaces of the supraoccipital (Fig. 18B). The caudal surface of the supraoccipital is flat and is nearly vertical ventral to the parietal process (Fig. 18D), as in *Dakotadon* (SDSM 8656), *Lurdusaurus* (MNHN GDF 1700), *Iguanodon bernissartensis* [29], *Mantellisaurus* (NHMUK R11521), *Ouranosaurus* [26], and “*Probactrosaurus*” *gobiensis* [42].

### Opisthotic-Exoccipital

In all known examples, the opisthotic and exoccipital are fully fused together with no apparent suture; the fused opisthotic-exoccipital complex is herein abbreviated as OE. The paroccipital process projects caudolaterally from the caudodorsal margin of the OE (Figs. 15A; 18E, G); the process is pendant and curves rostrally at its distal end in unbroken examples (e.g., CEUM 35475), as in *Ouranosaurus* [26], *Bactrosaurus* [38], *Lophorhothon* [53], and hadrosaurids [48]. The paroccipital process bears a rugose groove on its rostrolateral surface that would contact the medial margin of the postquadrate process of the squamosal (Fig. 18G). Rostroventral to the base of the paroccipital process is another sutural surface, in this case for the ventrolateral surface of the supraoccipital (Fig. 18G, H). Ventral to this supraoccipital suture is a dorsoventrally tall sutural surface for the caudal margin of the prootic (Fig. 18G, H); the prootic suture continues onto the lateral surface of the OE to articulate with a tab-like process on the caudodorsal margin of the prootic (Fig. 18G) (see below). Medial to the prootic suture is a medially-directed process that would meet the other OE to form the dorsal margin of the foramen magnum (Fig. 18H); this medial process is broken in the disarticulated examples of the OE, but can be observed underlying the supraoccipital on articulated braincases (Fig. 18E, F).

Caudal to the prootic suture, the lateral surface of the OE is pierced by two foramina; the more rostral of the two foramina is the exit for cranial nerves X and XI, while the other foramen is the exit for cranial nerve XII (Figs. 15B; 18G). Ventral to the cranial nerve exits, the OE expands caudally to form a robust, rostrocaudally long, and mediolaterally wide sutural surface for the basioccipital (Fig. 18G, H). Articulated braincases demonstrate that the basioccipital participated in the ventral margin of the foramen magnum, along with the left and right OEs (Fig. 18I).

### Prootic

**Figure 19:**
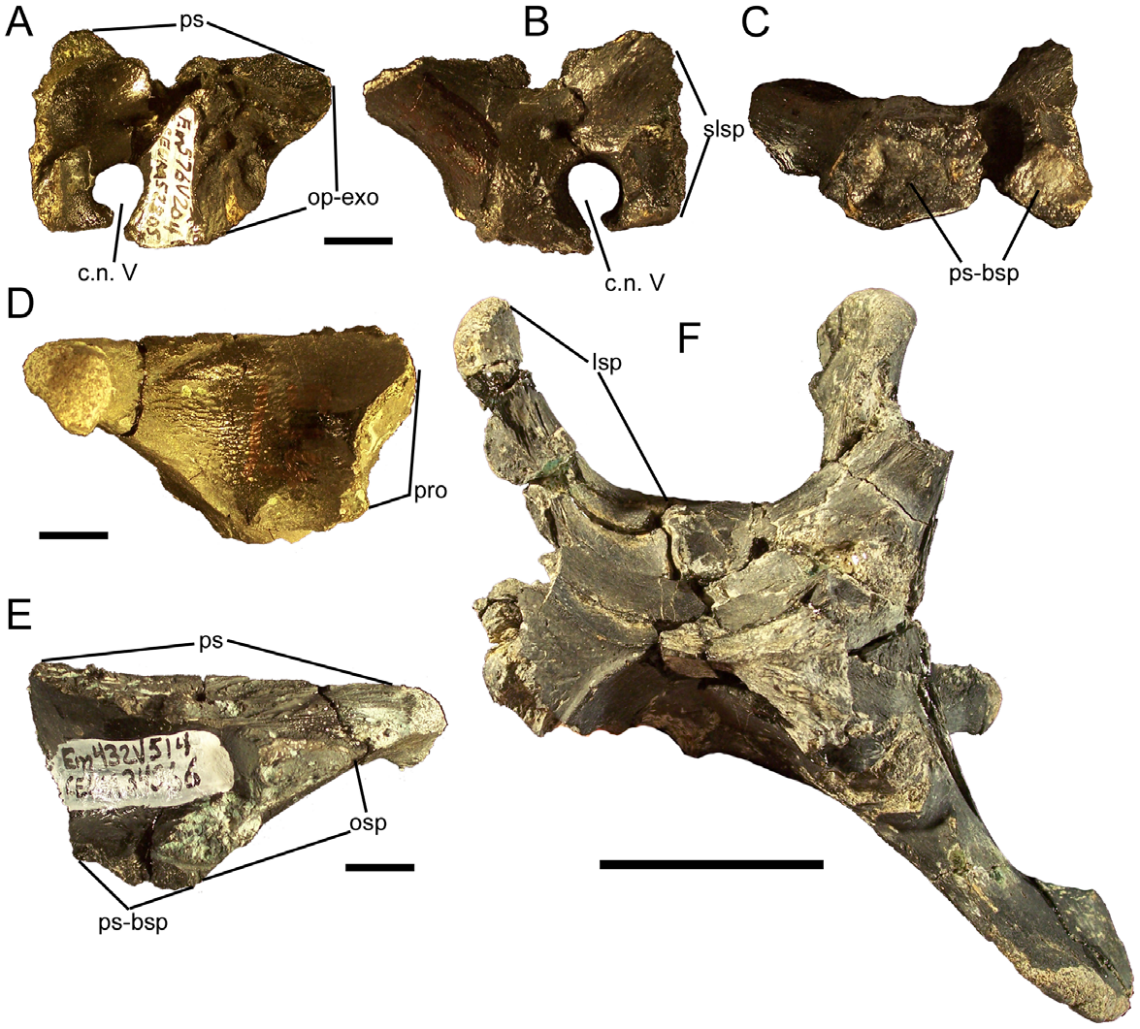
Prootic and laterosphenoid of *Eolambia*. Right prootic CEUM 52805 (WS8) in (A) medial, (B) lateral, and (C) ventral views. Left laterosphenoid CEUM 34366 (Eo2) in (D) lateral and (E) medial views. Braincase CEUM 74552 (Eo2) in dorsal view. *Abbreviations*: *c.n. V*, exit for cranial nerve V; *lsp*, laterosphenoid; *op-exo*, suture for opisthotic-exoccipital; *osp*, suture for orbitosphenoid; *pro*, suture for prootic; *ps*, parietal suture; *ps-bsp*, suture for parasphenoid-basisphenoid; *slsp*, suture for laterosphenoid. Scale bars in A–E equal 1 cm; scale bar in F equals 5 cm.

The prootic constitutes a major portion of the lateral wall of the braincase. The entire caudal margin of the prootic forms a suture for the opisthotic-exoccipital complex; in addition, a flange on the caudodorsal margin of the prootic would overlap the lateral surface of the opisthotic-exoccipital (Figs. 15B; 19A). The dorsal margin is entirely occupied by a sutural surface for the parietal (Fig. 19A). The lateral surface of the prootic is pierced by two foramina. The smaller of the two is a neurovascular foramen, but the larger foramen is the exit for cranial nerve V (Figs. 15B; 19A, B). The rostral margin of the prootic forms a sutural surface for the caudal surface of the laterosphenoid (Fig. 19B). The exit for cranial nerve V divides the ventral margin of the prootic into two processes, both of which bear sutural surfaces for the fused parasphenoid-basisphenoid complex on their ventral surfaces (Fig. 19C).

### Laterosphenoid

The caudal margin of the laterosphenoid bears a sutural surface for the rostral margin of the prootic (Fig. 19D). The ventral margin bears a suture for the fused parasphenoid-basisphenoid complex (Fig. 19E). The rostroventral margin exhibits a suture for the orbitosphenoid (Fig. 19E). The dorsal margin of the laterosphenoid bears a sutural surface for the parietal (Fig. 19E); this suture curves laterally to follow the lateral curvature of the rostral end of the parietal (see above). Indeed, the entire laterosphenoid curves laterally along its length, such that the rounded, rugose rostral end will rest in the laterosphenoid facet on the medial surface of the postorbital (Fig. 19D, F).

### Parasphenoid-Basisphenoid

**Figure 20:**
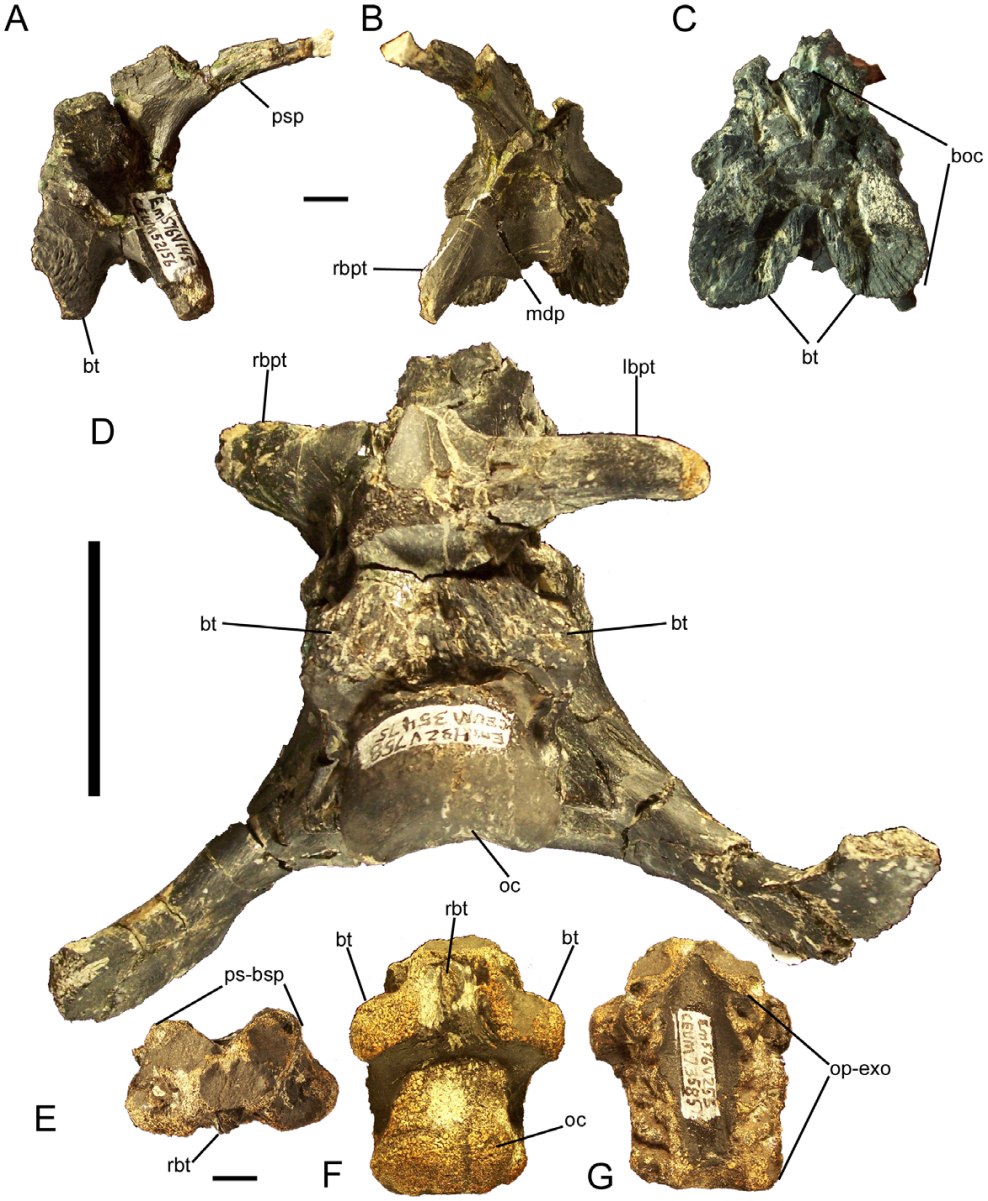
Parasphenoid-basisphenoid and basioccipital of *Eolambia*. Parasphenoid-basisphenoid CEUM 52156 (WS8) in (A) right lateral, (B) rostral, and (C) caudal views. Braincase CEUM 35475 (Eo2) in (D) ventral view. Basioccipital CEUM 73585 (WS8) in (E) rostral, (F) ventral, and (G) dorsal views. *Abbreviations*: *boc*, suture for basioccipital; *bt*, basal tuber; *lbpt*, left basipterygoid process; *mdp*, median prong; *oc*, occipital condyle; *op-exo*, suture for opisthotic-exoccipital; *ps-bsp*, suture for parasphenoid-basisphenoid; *psp*, parasphenoid process; *rbpt*, right basipterygoid process; *rbt*, ridge between basal tubera. Scale bars in A–C and E–G equal 1 cm; scale bar in D equals 5 cm.

In all known examples, the parasphenoid and basisphenoid are fully fused to each other and lack a visible suture; the fused parasphenoid-basisphenoid complex is herein abbreviated as PB. The parasphenoid process curves rostrodorsally from the rostral margin of the PB (Fig. 20A). Caudal to the base of the parasphenoid process would presumably be the sutural surfaces for the presphenoid, orbitosphenoids, laterosphenoids, and prootics, as well as the ventral margin of the cranial nerve V exit and the exit for cranial nerve VII (Fig. 15B); however, the disarticulated and articulated examples of the PB are too badly damaged along their dorsal margins to discern those sutures and foramina.

Ventral to the base of the parasphenoid process are the left and right basipterygoid processes; these project ventrolaterally and are slightly curved caudally (Fig. 20B, D). Between the basipterygoid processes is a midline prong that projects ventrally, as in *Camptosaurus dispar* (YPM 1856A), *Ouranosaurus* [26], *Bactrosaurus* [38], and *Levnesovia* [36]; only one disarticulated PB, CEUM 52156 from the WS8 bonebed, preserves part of this midline prong (Fig. 20B). Caudal to the basipterygoid processes and the midline prong is an extensive sutural surface for the basioccipital (Fig. 20C). The ventral margin of this sutural surface is divided into two heavily rugose flanges, which would overlap corresponding protuberances on the ventral surface of the basioccipital (see below) and form the rostral halves of the basal tubera (Figs. 15; 20A–D).

### Basioccipital

Along with the parasphenoid-basisphenoid complex, the basioccipital forms the floor of the braincase. The rostral surface of the basioccipital bears an extensive sutural surface for the caudal surface of the parasphenoid-basisphenoid (Fig. 20E). Two prominent protuberances on the ventral surface of the basioccipital represent the caudal halves of the basal tubera (Fig. 20F); these bumps would be overlapped by the aforementioned rugose flanges on the caudal margin of the parasphenoid-basisphenoid. Between the basal tubera is a rostrocaudally-oriented, sharply defined ridge (Fig. 20E, F), as in *Camptosaurus dispar* (YPM 1880), *Uteodon aphanoecetes* (CM 15780), *Cumnoria prestwichii* (OXFUM J.3303), *Dakotadon* (SDSM 8656), and *Jintasaurus* [50]. The occipital condyle is caudoventrally-directed (Figs. 15A; 20D, F). The dorsal surface of the basioccipital exhibits two rostrocaudally elongate sutures for the left and right opisthotic-exoccipital complexes (Fig. 20G).

### Dentition

**Figure 21:**
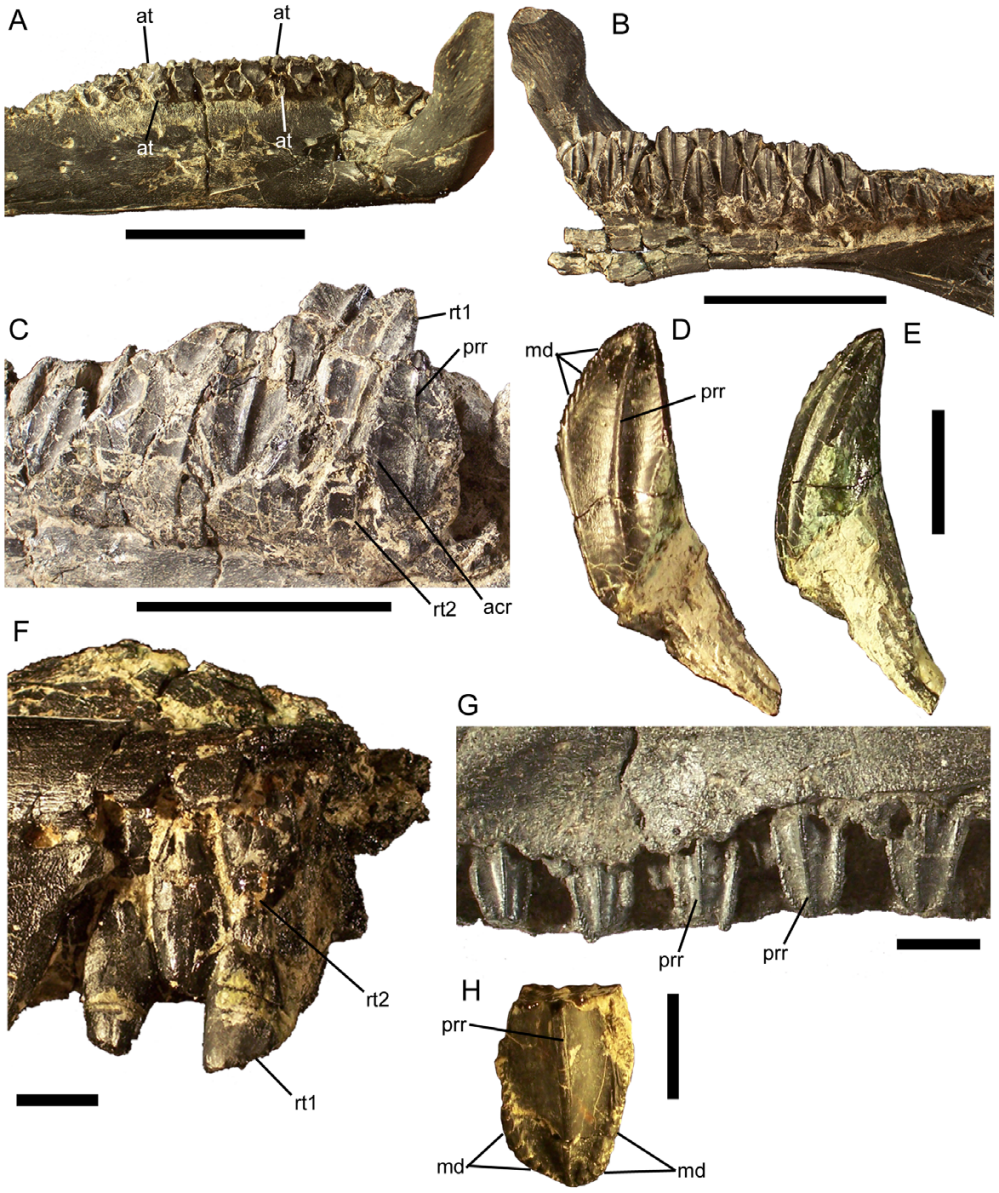
Dentition of *Eolambia*. Teeth in left dentary CEUM 74632 (Eo2) in (A) occlusal and (B) lingual views. Teeth in right dentary of CEUM 9758 (holotype) in (C) lingual view. Isolated dentary tooth CEUM 34261 (Eo2) in (D) lingual and (E) distal views. Teeth in right maxilla CEUM 35492 (Eo2) in (F) lingual view. Teeth in right maxilla CEUM 34356 (Eo2) in (G) labial view. Isolated maxillary tooth from the Eo2 bonebed in (H) labial view. *Abbreviations*: *acr*, accessory ridge; *at*, active tooth; *md*, marginal denticle; *prr*, primary ridge; *rt1*, first replacement tooth; *rt2*, second replacement tooth. Scale bars in A–C equal 5 cm; scale bars in D–H equal 1 cm.

The occlusal surface of the dentary is formed by the worn surfaces of active dentary teeth, with two active teeth in each alveolus (Fig. 21A); each active tooth bears a single concave wear facet. As in all iguanodonts for which the dentition is known, the dentary teeth of *Eolambia* are arranged in a series of interlocking rows without spaces between the crowns (Fig. 21B). There are two replacement teeth in each alveolus (Fig. 21C), as in *Probactrosaurus gobiensis* [13], *Jeyawati* [11], and *Bactrosaurus* [38]. Thus, a typical alveolus of *Eolambia* contains four teeth: two active teeth and two replacement teeth. The dentary tooth crowns are diamond-shaped in lingual view (Fig. 21B–D). The crowns of dentary teeth bear marginal denticles on the mesial and distal margins (Fig. 21B–D); the denticles are small mammillated papillae very similar to those of *Probactrosaurus gobiensis* (fig. 16B in [13]) (Fig. 21D). Each crown bears a distally-offset primary ridge, with either a faint mesial accessory ridge (e.g., adult holotype CEUM 9758, Fig. 21C) or no additional ridges (e.g., juvenile specimens from the Eo2 bonebed CEUM 74632 and CEUM 34261, Fig. 21B, D). The roots of the dentary teeth curve ventrolabially, making the labial surface of each tooth gently concave (Fig. 21E).

The maxillary teeth are arranged in a manner similar to that of the dentary teeth (Fig. 21F). In contrast to the dentary dentition, the maxillary dentition includes only one active tooth per alveolus (Fig. 10B); however, each maxillary alveolus holds two replacement teeth (Fig. 21F). Thus, it appears that each maxillary alveolus contained three teeth: one active tooth and two replacement teeth. As with the dentary teeth, each active maxillary tooth exhibits a single concave wear facet. The maxillary tooth crowns are lozenge-shaped in labial view (Fig. 21G, H). The marginal denticles of the maxillary tooth crowns are very similar in morphology to those of the dentary teeth (Fig. 21H). Each maxillary tooth crown bears a single distally-offset primary ridge (Fig. 21G, H). No other ridges are present, as in “*Probactrosaurus*” *mazongshanensis* [42], *Probactrosaurus gobiensis* [13], *Jeyawati* [11], *Protohadros* [10], *Shuangmiaosaurus* [34], *Bactrosaurus* [38], *Gilmoreosaurus* [40], *Levnesovia* [36], *Tethyshadros* [37], *Telmatosaurus* [51], *Claosaurus* [52], *Lophorhothon* [53], and hadrosaurids [48].

### Cervical vertebrae and ribs

With only two exceptions (see below), all of the vertebrae of *Eolambia* are disarticulated. Therefore, the approximate positions of the cervical, dorsal, sacral, and caudal vertebrae described in the following sections are based upon comparisons with basal iguanodonts for which complete or partial articulated vertebral columns are known, including *Camptosaurus dispar* (USNM 5473), *Uteodon aphanoecetes* (CM 11337), *Iguanodon bernissartensis* [29], and *Mantellisaurus atherfieldensis* [30].

**Figure 22:**
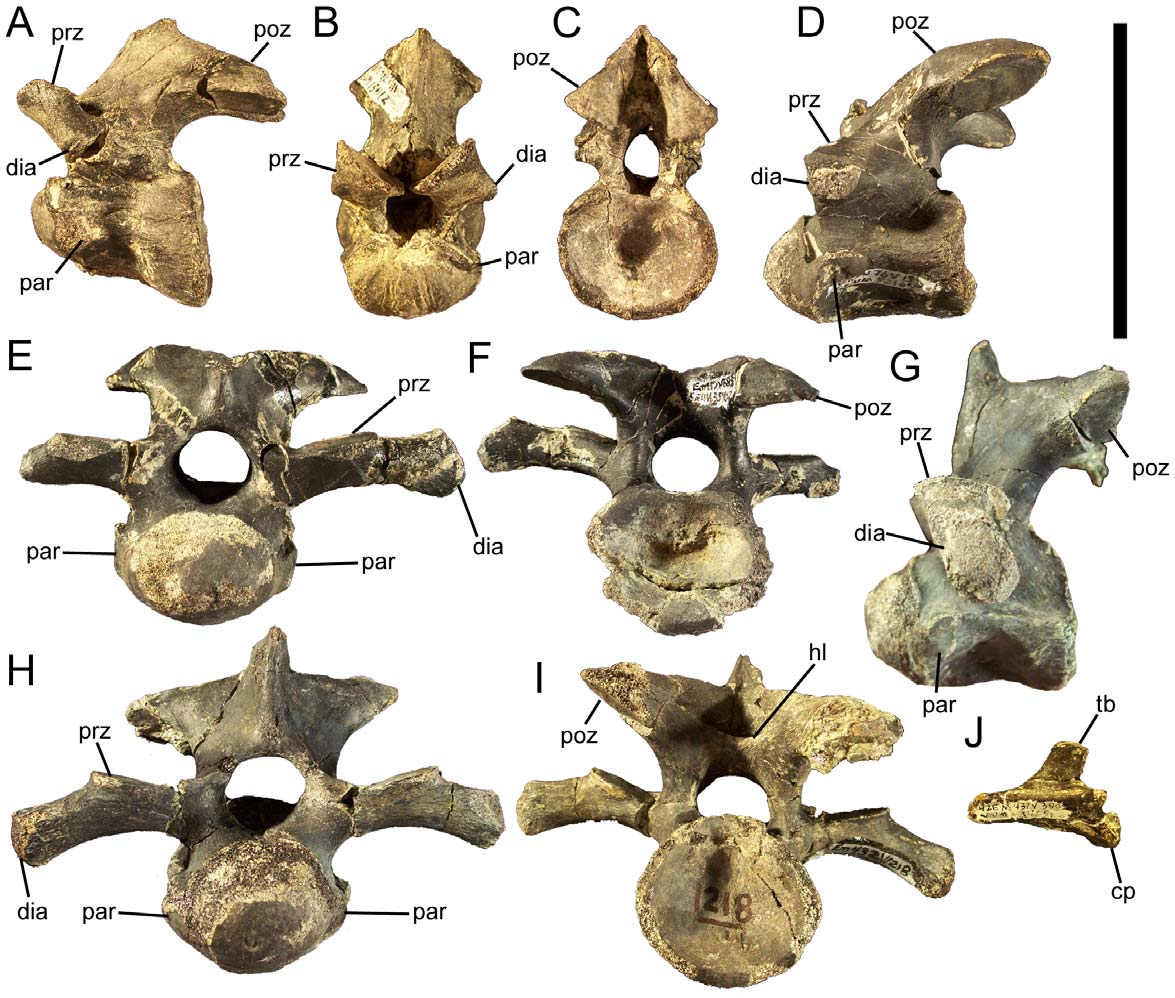
Cervical vertebrae of *Eolambia*. C3 CEUM 13412 (Eo2) in (A) left lateral, (B) cranial, and (C) caudal views. Middle cervical CEUM 52164 (WS8) in (D) left lateral view. Middle cervical CEUM 35402 (Eo2) in (E) cranial and (F) caudal views. Caudal cervical CEUM 74421 (Eo2) in (G) left lateral, (H) cranial, and (I) caudal views. Right cervical rib CEUM 34251 (Eo2) in (J) lateral view. *Abbreviations*: *cp*, capitulum; *dia*, diapophysis; *hl*, horizontal lamina; *par*, parapophysis; *poz*, postzygapophysis; *prz*, prezygapophysis; *tb*, tuberculum. Scale bar equals 10 cm.

The atlas and axis of *Eolambia* are unknown. The cranial-most identifiable cervical vertebra is CEUM 13412, a C3 from the *Eolambia* #2 bonebed. The cranial and caudal articulation surfaces are offset from each other, with the caudal surface situated farther ventrally in lateral view (Fig. 22A). The centrum is strongly opisthocoelous, with a well developed convex cranial surface and concave caudal surface (Fig. 22B, C); all preserved cervical vertebrae of *Eolambia* exhibit this strongly opisthocoelous morphology (see below). Numerous basal iguanodonts, including *Hippodraco* [7], *Lurdusaurus* [54], *Lanzhousaurus* [47], *Iguanodon bernissartensis* [29], *Mantellisaurus* [30], *Delapparentia* [55], *Ouranosaurus* [26], *Jinzhousaurus* [56], *Equijubus* [33], “*Probactrosaurus*” *mazongshanensis* [42], *Probactrosaurus gobiensis* [13], *Jeyawati* [11], *Bactrosaurus* [38], *Gilmoreosaurus* [40], *Tanius* [44], *Claosaurus* [52], and *Lophorhothon* [53] possess this morphology, as do hadrosaurids [48]. The parapophysis is a slight protuberance capped by a shallow facet on the lateral surface of the centrum near the cranial end; the diapophysis is a short, laterally-directed prong at the base of the pedestal atop which the prezygapophysis is situated (Fig. 22A, B). The flat articular surfaces of the prezygapophyses are directed dorsomedially (Fig. 22B). The neural arch slopes caudodorsally from between the prezygapophyses to the low, tab-like neural spine (Fig. 22A, B). The postzygapophyses are elongate processes that arise from the neural arch caudoventral to the neural spine and extend caudally, with a deep cleft in between; the articular surfaces face ventrolaterally (Fig. 22A, C).

None of the other cervical vertebrae exhibit the offset between the cranial and caudal articulation surfaces observed in C3; however, all cervicals exhibit prominently opisthocoelous centra, with strongly convex, hemispherical cranial surfaces and deeply concave caudal surfaces. The centra of middle cervical vertebrae bear a close resemblance to the centrum of C3, in being strongly opisthocoelous and in the location and morphology of the parapophyses (Fig. 22D–F). However, the neural arches of middle cervicals are quite different. The diapophyses are located at the ends of elongate, laterally directed transverse processes, near the bases of which are the prezygapophyses (Fig. 22D, E). The neural spine is still a short tab, but the postzygapophyses are much more widely separated than on the C3 and project caudolaterally (Fig. 22D, F). The centra of caudal cervical vertebrae are similar to those of more cranial cervicals in the degree of opisthocoely and placement of the parapophyses (Fig. 22G–I). The caudal cervical vertebrae also resemble middle cervicals in the morphology of the transverse processes, on which the diapophyses and prezygapophyses are located, and in the wide separation of the postzygapophyses (Fig. 22G–I). However, caudal cervicals differ in the possession of a deep depression bounded ventrally by a horizontal lamina between the postzygapophyses (Fig. 22I).Furthermore, the cranial margin of the neural spine is much steeper in caudal cervical vertebrae than in more cranial cervicals and the neural spine is more developed, forming a prominent spike with a triangular cross section (Fig. 22G, H).

The cervical ribs are roughly Y-shaped, with divergent tubercula and capitula. The tuberculum is subrectangular, transversely compressed, and dorsally directed (Fig. 22J). The capitulum is more robust and rugose and projects cranioventrally (Fig. 22J). Although the rib shafts are incomplete in all known cervical ribs, the preserved portions are straight and project caudally.

### Dorsal vertebrae and ribs

**Figure 23:**
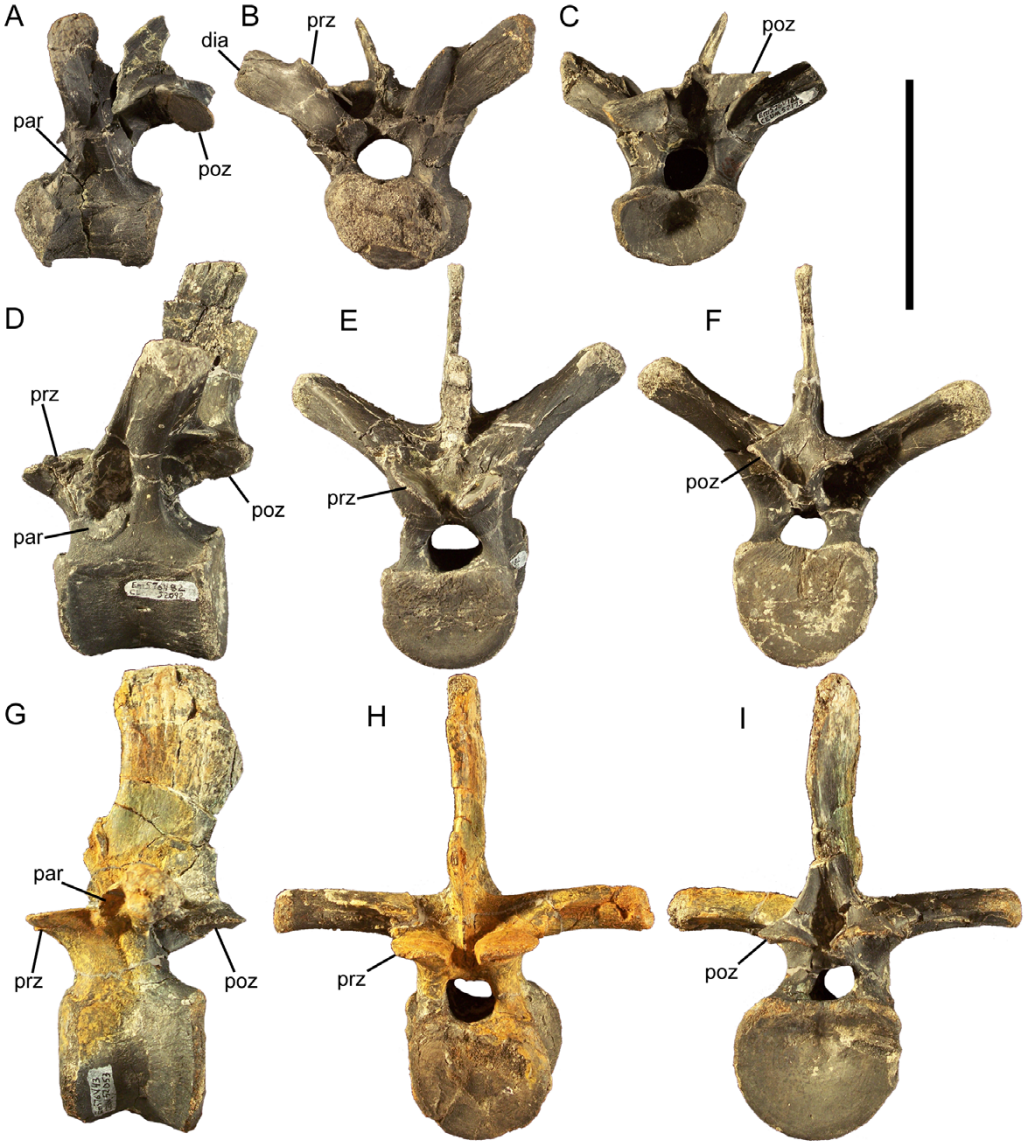
Dorsal vertebrae of *Eolambia*. Cranial dorsal CEUM 52173 (WS8) in (A) left lateral, (B) cranial, and (C) caudal views. Middle dorsal CEUM 52092 (WS8) in (D) left lateral, (E) cranial, and (F) caudal views. Caudal dorsal CEUM 52053 (WS8) in (G) left lateral, (H) cranial, and (I) caudal views. *Abbreviations*: *dia*, diapophysis; *par*, parapophysis; *poz*, postzygapophysis; *prz*, prezygapophysis. Scale bar equals 10 cm.

The cranial dorsal vertebrae are not very dissimilar from the caudal cervical vertebrae, with deeply opisthocoelous centra; dorsomedially-directed prezygapophyses located at the bases of the transverse processes; diapophyses located on the distal ends of the transverse processes; and elongate, caudolaterally-projecting, ventrolaterally-facing postzygapophyses (Fig. 23A–C). However, there are two notable differences. The parapophyses of cranial dorsal vertebrae are not on the lateral surface of the centrum, but rather on the base of the neural arch (Fig. 23A). Also, the neural spines of cranial dorsal vertebrae are more prominent, forming caudodorsally-curved prongs cranial to the bases of the postzygapophyses (Fig. 23A).

The middle dorsal vertebrae are different in a number of respects from the cranial dorsals. The centra are nearly amphiplatyan (Fig. 23D–F). The prezygapophyses are still dorsomedially directed, but are not located on the transverse processes; they are instead cranial to the bases of the processes (Fig. 23D, E). The parapophysis has migrated dorsally up the neural arch and is partially enclosed cranially by a lamina arising from the cranial edge of the transverse process and caudally by the base of the transverse processes itself (Fig. 23D). The postzygapophyses still face ventrolaterally, but are no longer located on the ends of elongate processes; they are instead simple pedestals that arise from the caudal margin of the base of the neural spine (Fig. 23D, F). Finally, neural spine is considerably taller than the centrum, rectangular, and caudally inclined Fig. 23D).

The caudal dorsal vertebrae are rather similar to the middle dorsals, retaining amphiplatyan centra and tab-like prezygapophyses and postzygapophyses (Fig. 23G–I). However, the articular surfaces of the prezygapophyses face dorsally rather than dorsomedially, and those of the postzygapophyses face ventrally rather than ventrolaterally (Fig. 23G–I). The parapophysis is now located actually on the transverse process, immediately distal to its base (Fig. 23G). The neural spine is vertical, with a concave cranial margin (Fig. 23G).

**Figure 24:**
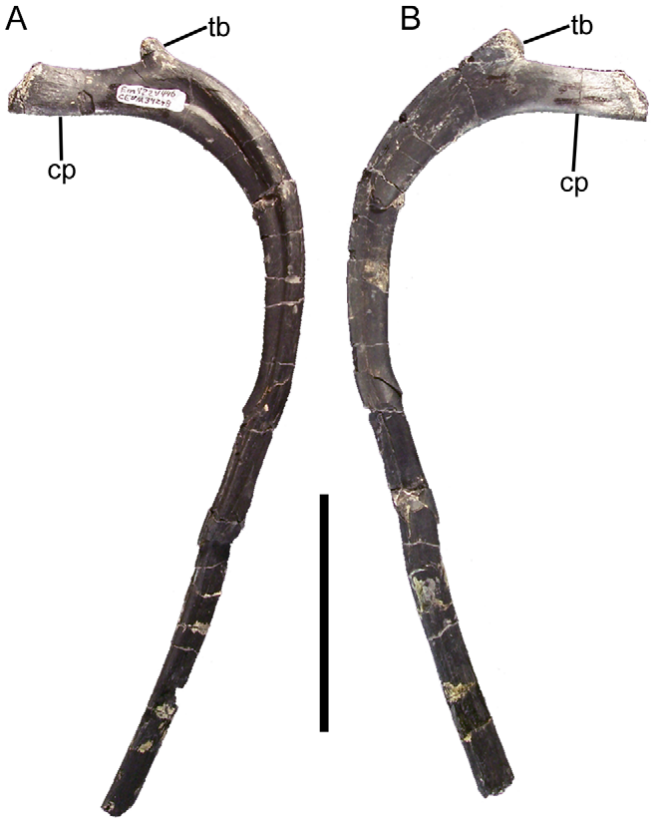
Dorsal rib of *Eolambia*. Left dorsal rib CEUM 34298 (Eo2) in (A) cranial and (B) caudal views. *Abbreviations*: *cp*, capitulum; *tb*, tuberculum. Scale bar equals 10 cm.

The dorsal ribs are divided into two processes, the capitulum and tuberculum, at their proximal ends. The capitulum is subrectangular and craniocaudally compressed with a rugose proximal end (Fig. 24). The tuberculum is a blunt protuberance dorsal to the base of the capitulum. There is a shallow groove on the cranial surface of the rib shaft, extending from ventral to the tuberculum to two-thirds down the shaft (Fig. 24A).

### Sacral vertebrae

**Figure 25:**
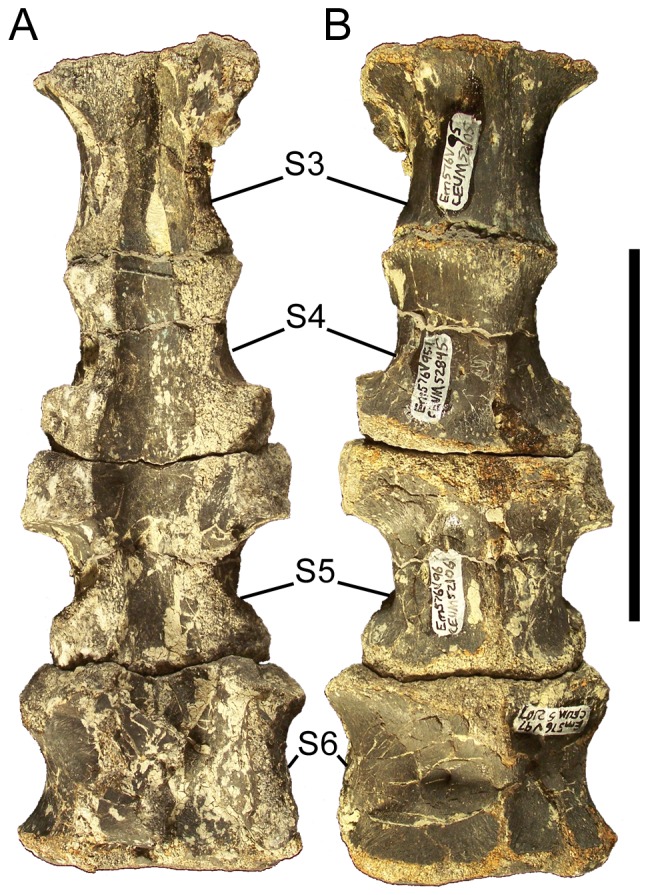
Sacral vertebrae of *Eolambia*. Articulated S3–S6 (CEUM 52105, 52845, 52106, and 52107; WS8) in (A) dorsal and (B) ventral views. Scale bar equals 10 cm.

In addition to numerous disarticulated sacral centra, there is a series of four articulated sacral centra from the Willow Springs 8 bonebed. Based upon comparison with the more complete articulated sacra of *Camptosaurus dispar* [57] and *Mantellisaurus atherfieldensis* [30], these articulated sacral vertebrae are identified as S3–S6. This sacral series well illustrates the changes in centrum shape that occur in the sacrum (Fig. 25). Sacral 3 is hourglass-shaped, with a cranial end that is much broader mediolaterally than the caudal end. Sacral 4 is stouter for its length than S3 and has a caudal end that is broader than the cranial. Sacral 5 is still more robust than S4 and is akin to S3 in having a broader cranial end than caudal end. Sacral 6 is the most blocky and robust of the series and differs from S3–S5 in having cranial and caudal ends that are approximately equal in width.

### Caudal vertebrae and chevrons

**Figure 26:**
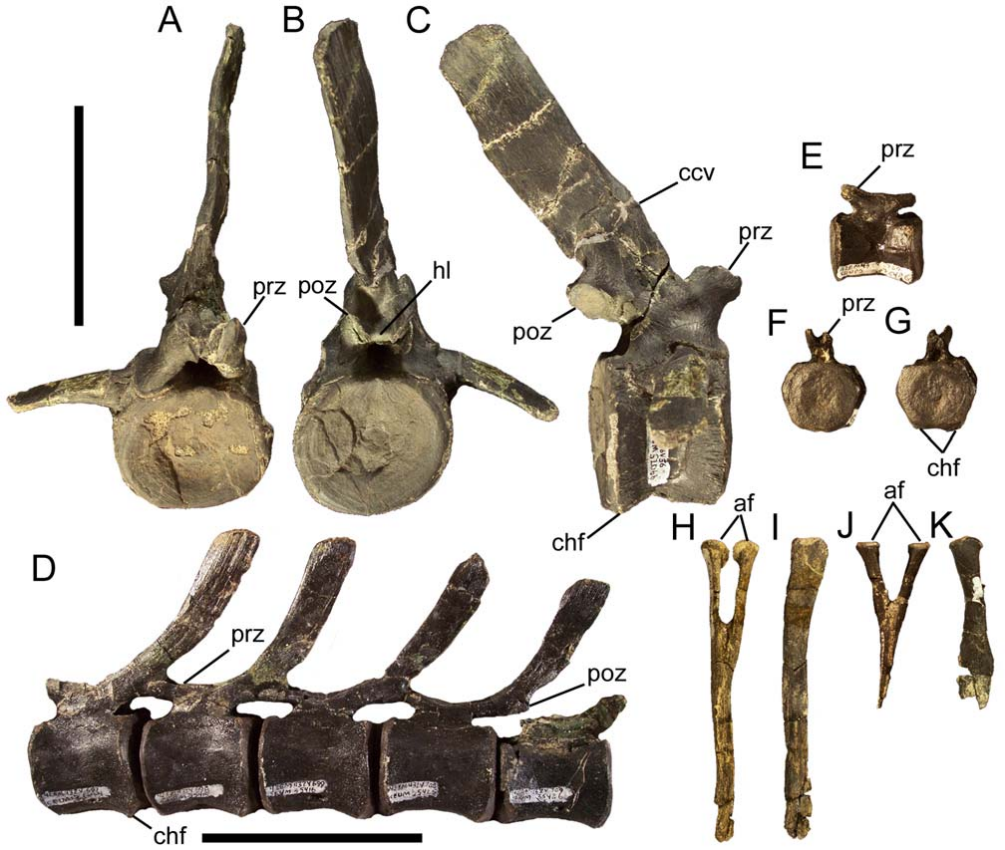
Caudal vertebrae and chevrons of *Eolambia*. Proximal caudal vertebra CEUM 52066 (WS8) in (A) cranial, (B) caudal, and (C) right lateral views. Articulated middle caudal vertebrae (CEUM 35414, 35415, 35416, 35425, and 35426; Eo2) in (D) left lateral view. Distal caudal vertebra CEUM 35486 (Eo2) in (E) left lateral, (F) cranial, and (G) caudal views. Proximal chevron CEUM 14457 (Eo2) in (H) cranial and (I) left lateral views. Distal chevron CEUM 34322 (Eo2) in (J) cranial and (K) left lateral views. *Abbreviations*: *af*, articulation facet; *ccv*, cranial convexity; *chf*, chevron facet; *hl*, horizontal lamina between postzygapophyses; *poz*, postzygapophysis; *prz*, prezygapophysis. Scale bars equal 10 cm.

The centra of proximal caudal vertebrae have almost circular and slightly concave cranial and caudal articulation surfaces (Fig. 26A, B). The caudoventral margin of the centrum bears a facet for the articulation of a proximal chevron (Fig. 26C). The dorsoventrally compressed transverse processes curve ventrolaterally from the dorsolateral margin of the centrum (Fig. 26A, B). The prezygapophyses arise as small pedestals from the cranial base of the neural spine; their flat articular surfaces are directed dorsomedially (Fig. 26A, C). The tab-like postzygapophyses arise from the caudal base of the neural spine and overhang the neural canal; their flat articular surfaces are directed ventrolaterally (Fig. 26B, C). Between the left and right postzygapophyses is a deep depression bounded ventrally by a horizontal lamina (Fig. 26B). The neural spine is caudally inclined and unexpanded at its distal end (Fig. 26C). Its cranial margin is nearly straight, save for a gentle convexity near its base (Fig. 26C).

The centra of middle caudal vertebrae are not dissimilar from those of the proximal caudals, except that the transverse processes are absent (Fig. 26D). There are two distinct chevron facets on the caudoventral surface of the centrum on either side of the midline. The prezygapophyses are situated on elongate stalks that project cranially; their flat articulation surfaces are directed dorsomedially. The postzygapophyses are little more than facets on the caudoventral margin of the neural spine; their flat articulation surfaces are directed ventrolaterally. The neural spines of middle caudal vertebrae are rather different from those of the proximal caudals, with strongly concave cranial margins and convex caudal margins; the neural spines are craniocaudally widest at their distal ends (Fig. 26D). The degrees of concavity of the cranial margin and convexity of the caudal margin increase distally down the caudal series.

The centra of distal caudal vertebrae are more elongate for their height than those of the proximal and middle caudals (Fig. 26E). The cranial and caudal articulation surfaces are very slightly concave and hexagonal (Fig. 26F, G). Two facets for articulation with a chevron are present on the caudoventral surface of the centrum as in the proximal and middle caudals, but these facets are more widely spaced in the distal caudals (Fig. 26G). The prezygapophyses project craniodorsally and their articular surfaces are directed dorsomedially (Fig. 26E, F). The morphologies of the postzygapophyses and neural spine are similar to those of the middle caudals, except that the neural spine is shorter compared to the height of the centrum.

The proximal chevrons bear two articulation facets atop the two processes that form the haemal canal; these facets are nearly in contact with each other (Fig. 26H). Distal to the haemal canal, the chevron is mediolaterally compressed, straight, and has parallel cranial and caudal margins (Fig. 26H, I). In the more distal chevrons, the articulation facets are more widely separated and the distal end of the chevron is craniocaudally expanded (Fig. 26J, K).

### Sternal

**Figure 27:**
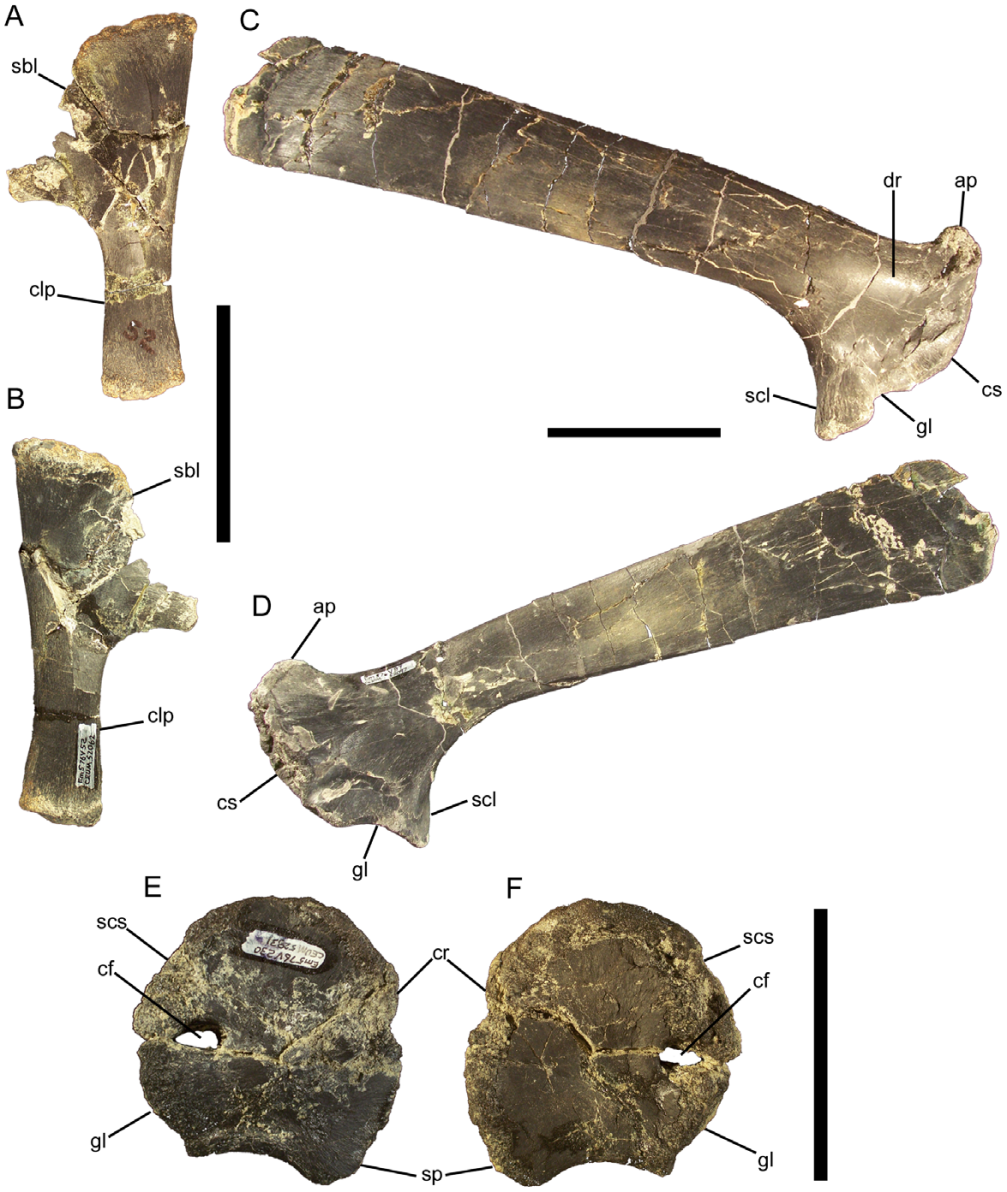
Pectoral girdle of *Eolambia*. Right sternal CEUM 52062 (WS8) in (A) dorsal and (B) ventral views. Right scapula CEUM 52097 (WS8) in (C) lateral and (D) medial views. Right coracoid CEUM 52831 (WS8) in (E) lateral and (F) medial views. *Abbreviations*: *ap*, acromion process; *cf*, coracoid foramen; *clp*, caudolateral process; *cr*, coracoid ridge; *cs*, coracoid suture; *dr*, deltoid ridge; *gl*, glenoid; *sbl*, sternal blade; *scl*, scapular labrum; *scs*, scapula suture; *sp*, sternal process. Scale bars equal 10 cm.

The sternal is a hatchet-shaped element with a broad sternal blade and an elongate, rectangular caudolateral process (Fig. 27A, B). The dorsal surface of the sternal is gently concave, whereas the ventral surface is slightly convex. The lateral margin of the sternal blade is straight and the medial margin is somewhat convex (Fig. 27A, B), as in *Fukuisaurus* [27], *Iguanodon bernissartensis* [29], *Ouranosaurus* [26], and *Jinzhousaurus* [56]. Although the sternal blade of the only known sternal, CEUM 52062, is broken along part of its medial edge, its shape can be inferred from the intact portions. The lateral and medial margins of the caudolateral process are roughly parallel in dorsal and ventral views; the caudal end of the caudolateral process is only slightly expanded mediolaterally (Fig. 27A, B). There is no caudomedial process arising from the sternal blade.

### Scapula

The cranial margin of the scapula is mediolaterally thickened along the articular surface for the coracoid. The scapular portion of the humeral glenoid is a depression on the cranioventral margin of the cranial end of the scapula (Fig. 27C, D). Caudal to the glenoid, the triangular scapular labrum projects ventrally. The dorsal margin of the cranial end of the scapular bears a prominent dorsally-projecting acromion process (Fig. 27C, D). The cranial margin of the acromion process is gently convex in lateral and medial views, whereas the caudal margin is shallowly concave. On the lateral surface of the scapula ventral to the acromion process is the deltoid ridge, a low rostrocaudally elongate eminence that diminishes in prominence caudally, eventually merging with the lateral surface of the scapular blade (Fig. 27C). The dorsal and ventral margins of the scapular blade are straight and diverge only slightly at the caudal end of the blade in lateral and medial views, such that they meet the caudal margin at nearly right angles (Fig. 27C, D), as in *Iguanodon bernissartensis* [29], *Ouranosaurus* [26], *Altirhinus* [32], *Probactrosaurus gobiensis* [13], and *Tanius* [44].

### Coracoid

The lateral surface of the coracoid is slightly convex, whereas the medial surface is concave. The caudoventral margin of the coracoid bears the coracoid portion of the humeral glenoid (Fig. 27E, F). The caudodorsal margin of the coracoid is thickened and rugose to form the sutural surface with the scapula. Near the caudal margin of the coracoid is the large elliptical coracoid foramen (Fig. 27E, F). The rostral margin of the coracoid bears the coracoid ridge, a rounded prominence that projects rostrally (Fig. 27E, F). The hook-like sternal process arises from the cranioventral margin of the coracoid and projects ventrally (Fig. 27E, F).

### Humerus

**Figure 28:**
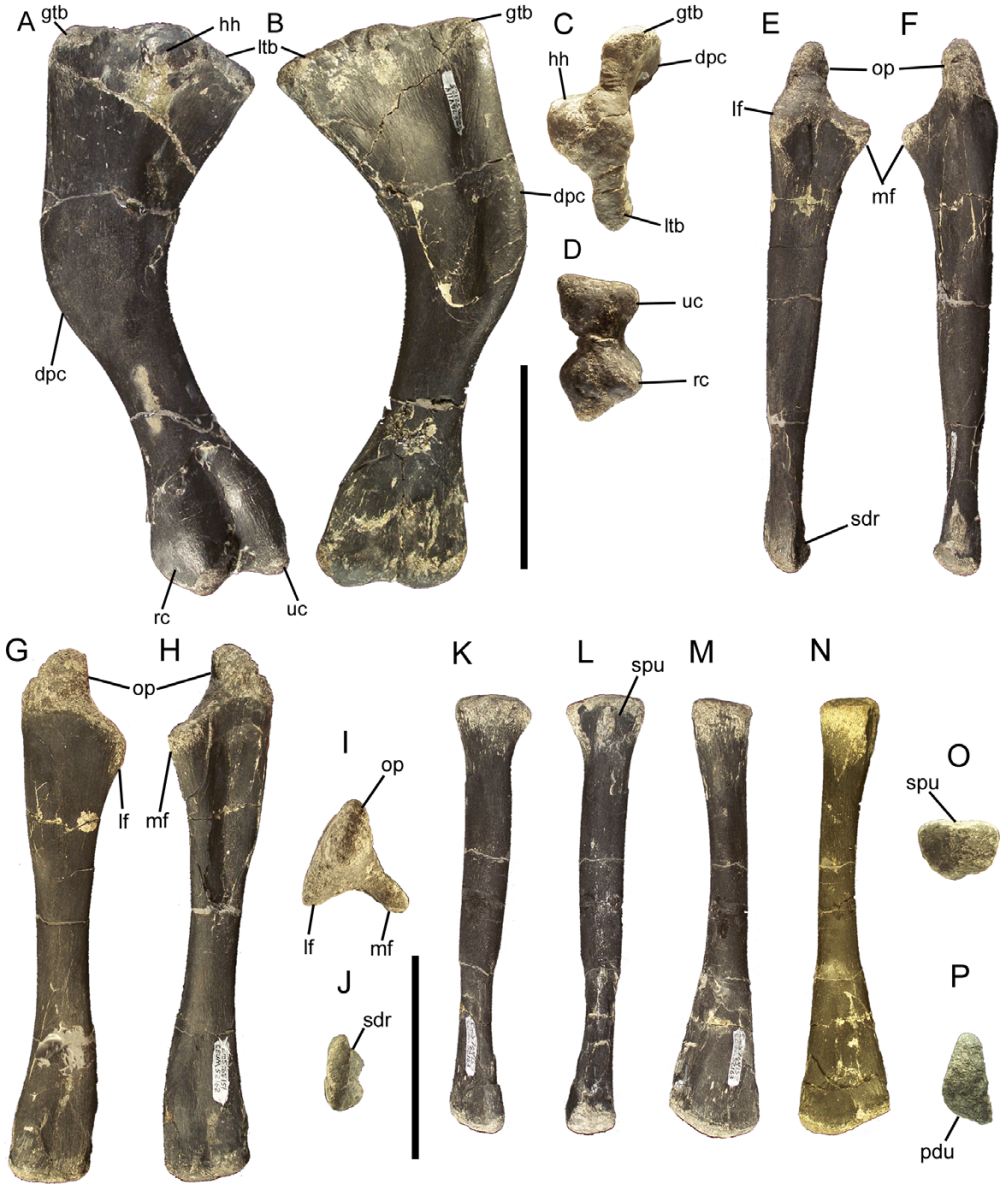
Forelimb elements of *Eolambia*. Left humerus CEUM 52125 (WS8) in (A) caudal, (B) cranial, (C) proximal, and (D) distal views. Right ulna CEUM 52162 (WS8) in (E) cranial, (F) caudal, (G) lateral, (H) medial, (I) proximal, and (J) distal views. Right radius CEUM 52163 (WS8) in (K) cranial, (L) caudal, (M) lateral, (N) medial, (O) proximal, and (P) distal views. *Abbreviations*: *dpc*, deltopectoral crest; *gtb*, greater tuberosity; *hh*, humeral head; *lf*, lateral flange; *ltb*, lateral tuberosity; *mf*, medial flange; *op*, olecranon process; *pdu*, platform that would contact distal end of ulna; *rc*, radial condyle; *sdr*, slot for distal end of radius; *spu*, surface that would contact proximal end of ulna; *uc*, ulnar condyle. Scale bars equal 10 cm.

The humerus is bowed medially in caudal and cranial views (Fig. 28A, B). The deltopectoral crest arises ventral to the greater tuberosity and extends along the lateral margin of the humerus before gently curving ventromedially and merging with the humeral shaft (Fig. 28A, B). The deltopectoral crest is thickened, while the cranial surface of the proximal end of the humerus is concave medial to the crest (Fig. 28B). The caudal surface of the proximal end of the humerus exhibits a prominent round humeral head (Fig. 28A, C). The greater tuberosity is a rugose ledge lateral to the humeral head; this ledge curves craniolaterally and merges with the deltopectoral crest (Fig. 28A–C). The lesser tuberosity is a ledge medial to the humeral head that projects medially (Fig. 28A–C). The distal end of the humerus is transversely expanded compared to the humeral shaft and is divided into two rounded condyles, the lateral radial condyle and the medial ulnar condyle (Fig. 28A). The radial and ulnar condyles are separated by cranial and caudal intercondylar grooves (Fig. 28D). The radial condyle is larger than the ulnar condyle and exhibits a prominent knob on its lateral surface (Fig. 28D).

### Ulna

The shaft of the ulna is straight in all views (Fig. 28E–H). The proximal end of the ulna is expanded craniocaudally and mediolaterally, and is divided into three prongs. The prominent olecranon process arises from the proximocaudal margin of the ulna and curves cranially (Fig. 28E–I). Distal to the olecranon process are two roughly triangular flanges that form a cradle for the proximal end of the radius; the lateral flange projects craniolaterally and the medial flange craniomedially (Fig. 28E–I). Distal to the lateral and medial flanges, the shaft of the ulna narrows mediolaterally (Fig. 28E, F) and expands craniocaudally (Fig. 28G, H) towards the distal end. The distal surface of the ulna is convex to form an articulation surface for the carpal bones. There is a shallow slot on the craniomedial surface of the distal end to receive the distal end of the radius (Fig. 28E, J).

### Radius

The shaft of the radius is straight in cranial and caudal views (Fig. 28K, L), but slightly bowed cranially in lateral and medial views (Fig. 28M, N). The proximal end of the radius is mediolaterally expanded (Fig. 28K, L) and almost round in proximal view (Fig. 28O). The caudal surface of the proximal end is flatter than the cranial surface, forming a surface that would rest against the cranial surface of the proximal end of the ulna (Fig. 28L, O). The shaft of the radius narrows mediolaterally (Fig. 28K, L) and expands craniocaudally (Fig. 28M, N) towards the distal end. The distal end of the radius is subtriangular in distal view, with a straight medial margin and convex lateral margin (Fig. 28P). The caudolateral margin of the distal forms a distinct platform that would articulate with the aforementioned slot at the distal end of the ulna (Fig. 28P). The distal surface of the radius is convex to articulate with the proximal surfaces of the carpal bones.

### Metacarpals and manual phalanges

An articulated manus is not available in the known material of *Eolambia*. Thus, the articulated manūs of other iguanodonts, such as *Iguanodon bernissartensis* (IRSNB 1534) [29], *Mantellisaurus atherfieldensis* (IRSNB 1551) [30], and *Ouranosaurus nigeriensis* [26], have been used to interpret the disarticulated manual material of *Eolambia*.

**Figure 29:**
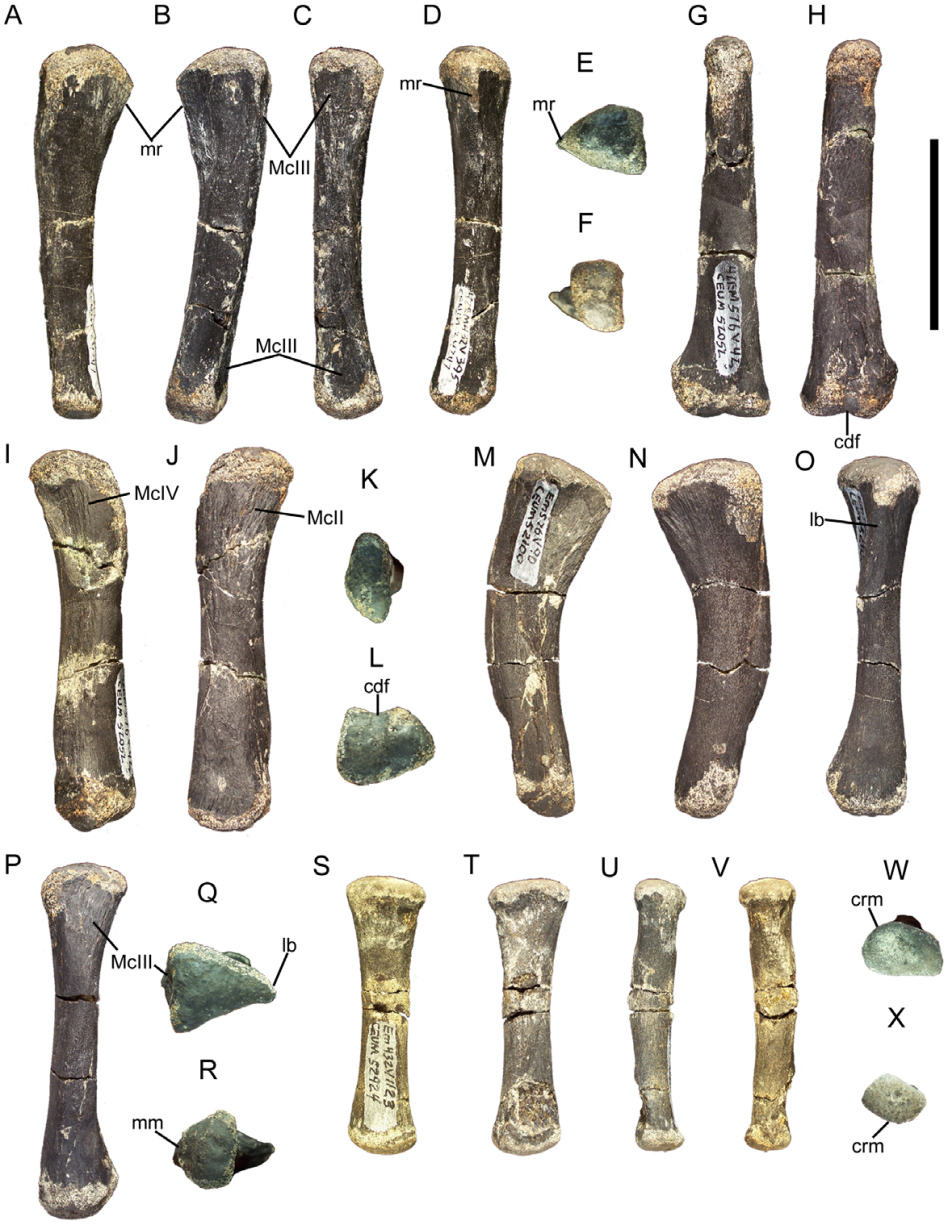
Metacarpals of *Eolambia*. Right metacarpal II CEUM 34247 (Eo2) in (A) cranial, (B) caudal, (C) lateral, (D) medial, (E) proximal, and (F) distal views. Right metacarpal III CEUM 52052 (WS8) in (G) cranial, (H) caudal, (I) lateral, (J) medial, (K) proximal, and (L) distal views. Left metacarpal IV CEUM 52100 (WS8) in (M) cranial, (N) caudal, (O) lateral, (P) medial, (Q) proximal, and (R) distal views. Right metacarpal V CEUM 52924 (Eo2) in (S) cranial, (T) caudal, (U) lateral, (V) medial, (W) proximal, and (X) distal views. *Abbreviations*: *cdf*, caudodistal furrow; *crm*, cranial margin; *lb*, lateral buttress; *McII*, surface for metacarpal II; *McIII*, surface for metacarpal III; *McIV*, surface for metacarpal IV; *mm*, medial margin; *mr*, medial ridge. Scale bar equals 5 cm.

Metacarpal II gently curves medially in cranial and caudal views (Fig. 29A, B). The proximal end is expanded mediolaterally, whereas the distal end is not markedly expanded relative to the shaft (Fig. 29A, B). The lateral surface of metacarpal II is flat, forming a surface against which the medial surface of metacarpal III would abut (Fig. 29B, C). On the medial surface of metacarpal II, a sharp ridge extends from the proximal articular surface to approximately one-fourth of the way down the shaft (Fig. 29A, B, D). This ridge gives the proximal articular surface of metacarpal II a teardrop-shape in proximal view (Fig. 29E). The distal articular surface is oblong in distal view and is mediolaterally wider along its caudal margin than its cranial margin (Fig. 29F). Both the proximal and distal articular surfaces of metacarpal II are strongly convex.

Metacarpal III is quite different in overall shape from metacarpal II; the shaft is straight, the proximal end tapers towards the proximal articular surface, and the distal end is mediolaterally expanded (Fig. 29G, H). The proximal end does curve cranially in lateral and medial views (Fig. 29I, J). The lateral surface of metacarpal III exhibits a striated facet near its proximal end for articulation with the proximal end of metacarpal IV (Fig. 29I). The medial surface bears a corresponding facet for articulation with the proximal end of metacarpal II (Fig. 29J). The proximal articular surface of metacarpal III is mediolaterally narrow in proximal view, with a gently convex medial margin and straight lateral margin (Fig. 29K). The distal articular surface is cleft by a shallow furrow on its caudodistal aspect, dividing the distal articular surface into medial and lateral portions (Fig. 29H, L). The medial portion is craniocaudally longer, whereas the lateral portion is mediolaterally wider (Fig. 29L).

Metacarpal IV is strongly curved laterally in cranial and caudal views (Fig. 29M, N). Metacarpal IV is mediolaterally widest at its proximal end; the shaft narrows slightly as it curves laterally, and then becomes markedly narrower near the distal end. The distal end is considerably expanded craniocaudally compared to the proximal end in lateral and medial views (Fig. 29O, P). The lateral surface bears a robust buttress that extends from the proximal articular surface to approximately one-fourth of the way down the length of the bone (Fig. 29O). This buttress makes the proximal articular surface triangular in proximal view (Fig. 29Q). The medial surface near the proximal articular surface forms a triangular facet for articulation with the proximal end of metacarpal III (Fig. 29P, Q). The distal articular surface is subcircular, with a prominently convex medial margin and straight lateral margin (Fig. 29R).

Metacarpal V is straight for its entire length with proximal and distal ends that are somewhat expanded mediolaterally and craniocaudally (Fig. 29S–V). The proximal articular surface is roughly elliptical in proximal view, with a convex cranial margin and a straight caudal margin (Fig. 29W). The distal articular surface is subrectangular in distal view (Fig. 29X).

**Figure 30:**
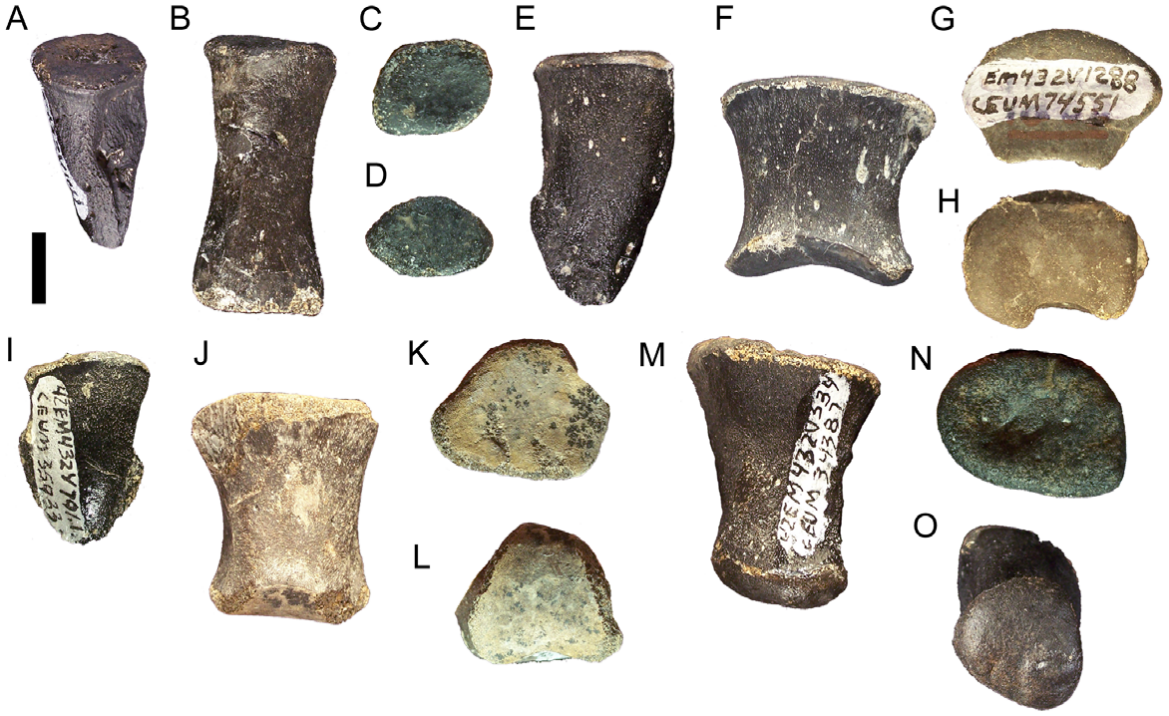
Manual elements of *Eolambia*. Left or right ungual of digit I CEUM 52962 (Eo2) in (A) plantar view. Left phalanx 1 of digit II CEUM 35585 (Eo2) in (B) dorsal, (C) proximal, and (D) distal views. Right ungual of digit II CEUM 36515 (Eo2) in (E) dorsal view. Left phalanx 1 of digit III CEUM 74551 (Eo2) in (F) dorsal, (G) proximal, and (H) distal views. Right ungual of digit III CEUM 35933 (Eo2) in (I) dorsal view. Right phalanx 1 of digit IV CEUM 35738 (Eo2) in (J) dorsal, (K) proximal, and (L) distal views. Right phalanx 1 of digit V CEUM 34387 (Eo2) in (M) dorsal, (N) proximal, and (O) distal views. In all images of elements in proximal or distal view, dorsal is towards the top of the figure and plantar towards the bottom. Scale bar equals 1 cm.

The ungual of digit I is a distinctive conical element as in many other basal iguanodonts, including *Uteodon aphanoecetes* [39], *Lurdusaurus* [54], *Barilium* [58], *Iguanodon bernissartensis* [29], *Mantellisaurus* [30], *Ouranosaurus* [26], *Altirhinus* [32], *Jinzhousaurus* [56], and *Probactrosaurus gobiensis* [13]. The proximal articular surface is circular with a deep depression at its center. The plantar surface bears a deep longitudinal groove that narrows towards the distal tip of the ungual (Fig. 30A).

Phalanx 1 of digit II is hourglass-shaped, with mediolaterally expanded proximal and distal ends (Fig. 30B). The proximal articular surface is rhomboidal and very shallowly concave, and the distal articular surface is elliptical and slightly convex (Fig. 30C, D). The ungual of digit II is hoof-like, though it does taper towards its distal tip and curves laterally along its length (Fig. 30E). The proximal articular surface is elliptical with a deep depression at its center.

Phalanx 1 of digit III is considerably stouter for its length than the corresponding element of digit II, with very broad articular surfaces (Fig. 30F). The proximal articular surface is roughly elliptical, but with a concave ventral margin, and is shallowly concave (Fig. 30G). The distal articular surface is subrectangular with a hook-like flange projecting from its plantar margin and is somewhat convex (Fig. 30H). The ungual of digit III is similar to that of digit II, but does not curve along its length (Fig. 30I). Its proximal articular surface is subcircular with a deep depression at the center. The ungual is hoof-like, with rugose flanges on its lateral and medial margins, as in *Iguanodon bernissartensis* [29], *Mantellisaurus* [30], *Ouranosaurus* [26], *Altirhinus* [32], *Jinzhousaurus* [56], *Probactrosaurus gobiensis* [13], *Protohadros* [10], *Nanyangosaurus* [43], *Tethyshadros* [37], *Lophorhothon* [53], and hadrosaurids [48].

Phalanx 1 of digit IV is similar in shape to the corresponding element of digit III (Fig. 30J). Both articular surfaces are subtriangular (Fig. 30K, L); the proximal articular surface is slightly concave and the distal articular surface is strongly convex.

Phalanx 1 of digit V is rather dissimilar in shape from the first phalanges of the other digits. Its proximal end is considerably than its distal end (Fig. 30M). The proximal articular surface is elliptical and deeply concave, whereas the distal articular surface is subtriangular and prominently convex (Fig. 30N, O).

### Ilium

**Figure 31:**
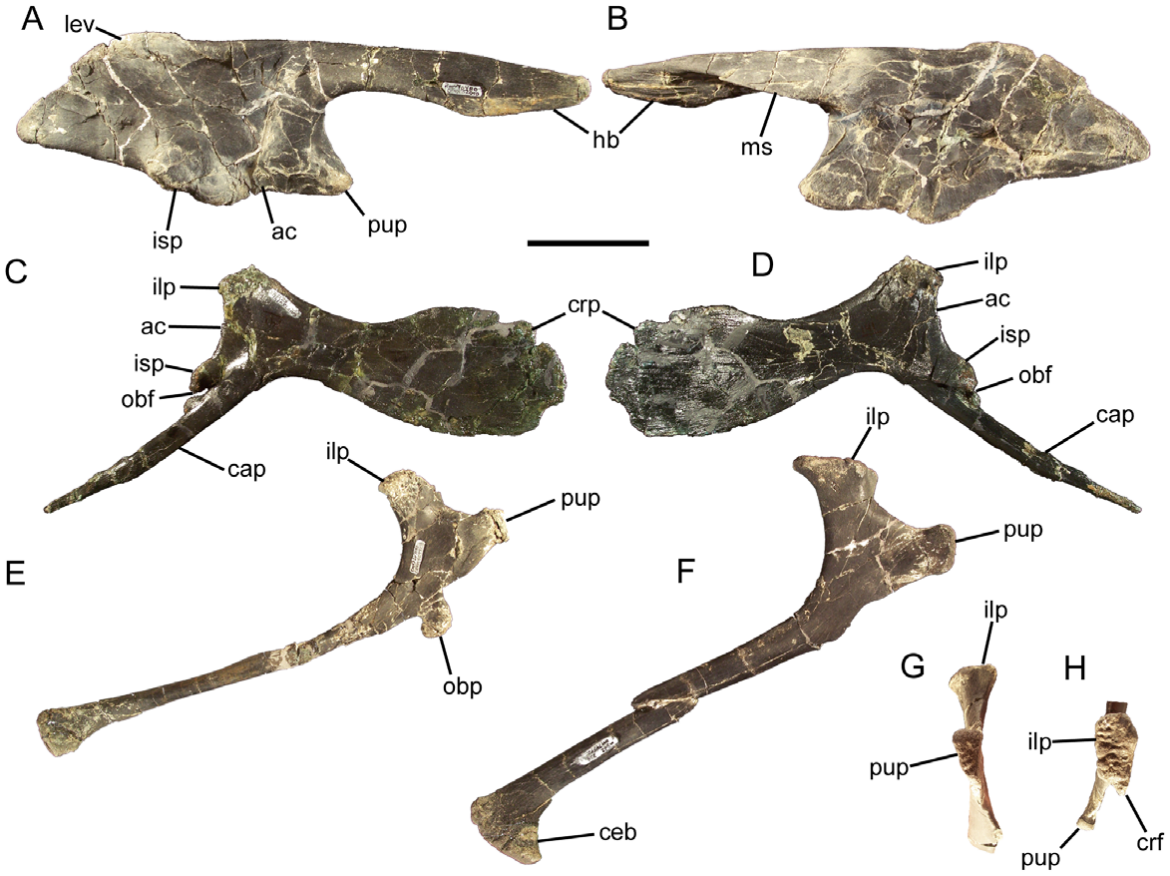
Pelvic girdle of *Eolambia*. Right ilium CEUM 52090 (WS8) in (A) lateral and (B) medial views. Right pubis CEUM 52152 (WS8) in (C) lateral and (D) medial views. Right ischium CEUM 52941 (Eo2) in (E) lateral view. Right ischium CEUM 74572 (Eo2) in (F) lateral, (G) cranial, and (H) proximal views. *Abbreviations*: *ac*, acetabulum; *cap*, caudal pubic process; *ceb*, cranially expanded boot; *crf*, cranial flange; *crp*, cranial pubic process; *hb*, horizontal boot; *ilp*, iliac peduncle; *isp*, ischial peduncle; *lev*, laterally everted rim; *ms*, medial shelf; *obp*, obturator process; *obf*, obturator foramen; *pup*, pubic peduncle. Scale bar equals 10 cm.

The preacetabular process of the ilium projects cranioventrally and terminates in a horizontal boot that is offset from and forms an obtuse angle with the ventral margin of the process (Fig. 31A, B), as in *Iguanacolossus* [7], *Cedrorestes* [6], *Planicoxa* [4], *Osmakasaurus depressus* [5], [39], *Barilium* [58], *Iguanodon bernissartensis* [29], *Mantellisaurus* [30], *Delapparentia* [55], *Ouranosaurus* [26], *Xuwulong* [35], and *Probactrosaurus gobiensis* [13]. A prominent shelf originates on the medial surface of the preacetabular process dorsal to the horizontal boot; this shelf expands caudally until it comprises the entire medial surface of the preacetabular process where the process merges with the body of the ilium (Fig. 31B). The cranial margin of the body of the ilium curves cranioventrally from the base of the preacetabular process, ending in the cranial end of the pubic peduncle (Fig. 31A). The pubic peduncle is mediolaterally thickened and extends caudally along the ventral margin of the ilium until it reaches the acetabulum. The acetabulum is quite shallow, forming a broad embayment in lateral view (Fig. 31A). Caudal to the acetabulum, the transversely thickened ischial peduncle extends caudodorsally along the ventral margin of the ilium (Fig. 31A).

The dorsal margin of the ilium is straight dorsal to the pubic peduncle and acetabulum (Fig. 31A). The dorsal margin thickens caudally to form a laterally everted rim dorsal to the ischial peduncle (Fig. 31A), as in *Iguanodon bernissartensis* [29], *Mantellisaurus* [30], *Delapparentia* [55], *Ouranosaurus* [26], *Altirhinus* [32], *Jinzhousaurus* [56], *Xuwulong* [35], and *Probactrosaurus gobiensis* [13]. Caudal to this laterally everted rim, the dorsal margin of the postacetabular process abruptly slopes caudoventrally to form a well demarcated platform for the origin of *M. iliocaudalis*. The dorsal and ventral margins of the postacetabular process converge caudally such that the process tapers to a point (Fig. 31A). The medial surface of ilium CEUM 52090 (WS8) is badly cracked, obscuring details such as the facets for the sacral ribs (Fig. 31B). However, CEUM 14601, a smaller right ilium from the Eo2 bonebed, exhibits at least three sacral rib facets on its medial surface. There is no brevis shelf on the medial surface of the postacetabular process (Fig. 31B).

### Pubis

The cranial pubic process is expanded, with dorsal and ventral margins that diverge towards its cranial end (Fig. 31C, D), as in *Lurdusaurus* [54], *Barilium* [58], *Lanzhousaurus* [47], *Iguanodon bernissartensis* [29], *Mantellisaurus* [30], *Delapparentia* [55], *Ouranosaurus* [26], *Altirhinus* [32], *Xuwulong* [35], “*Probactrosaurus*” *mazongshanensis* [42], *Probactrosaurus gobiensis* [13], *Bactrosaurus* [38], *Gilmoreosaurus* [40], *Levnesovia* [36], *Tethyshadros* [37], *Huehuecanauhtlus* [59], and hadrosaurids [48]. The ventral margin of the cranial pubic process is gently convex, whereas the dorsal margin is strongly concave. The iliac peduncle is a subtriangular projection caudodorsal to the base of the cranial pubic process. The caudal margin of the iliac peduncle curves caudoventrally to form the cranial margin of the acetabulum (Fig. 31C, D). Caudoventral to the base of the cranial pubic process is the base of the caudal pubic process. The caudal pubic process curves caudoventrally and tapers to a point (Fig. 31C, D). It is very probable that the caudal pubic process was shorter than the ischium. The ischial peduncle of the pubis is a ventrally-curving flange on the caudal margin of the caudal pubic process near its base; the caudal surface of this peduncle is flat, forming the contact surface for the pubic peduncle of the ischium (Fig. 31C, D). The ischial peduncle and a small knob ventral to it define the obturator foramen.

### Ischium

The proximal end of the ischium is divided into two subrectangular processes, the cranially-projecting pubic peduncle and the dorsally-projecting iliac peduncle (Fig. 31E, F). The sutural surfaces of both peduncles are highly rugose (Fig. 31G, H). The sutural surface of the pubic peduncle is roughly triangular in cranial view, becoming wider dorsally (Fig. 31G). The sutural surface of the iliac peduncle is roughly oval with a flange projecting cranially from its craniomedial margin (Fig. 31H). The shaft of the ischium is straight (Fig. 31E, F). The hook-like obturator process projects cranioventrally from the cranial margin of the shaft near the proximal end of the ischium (Fig. 31E). The shaft of the ischium terminates in a mediolaterally compressed and cranially expanded boot (Fig. 31F).

### Femur

**Figure 32:**
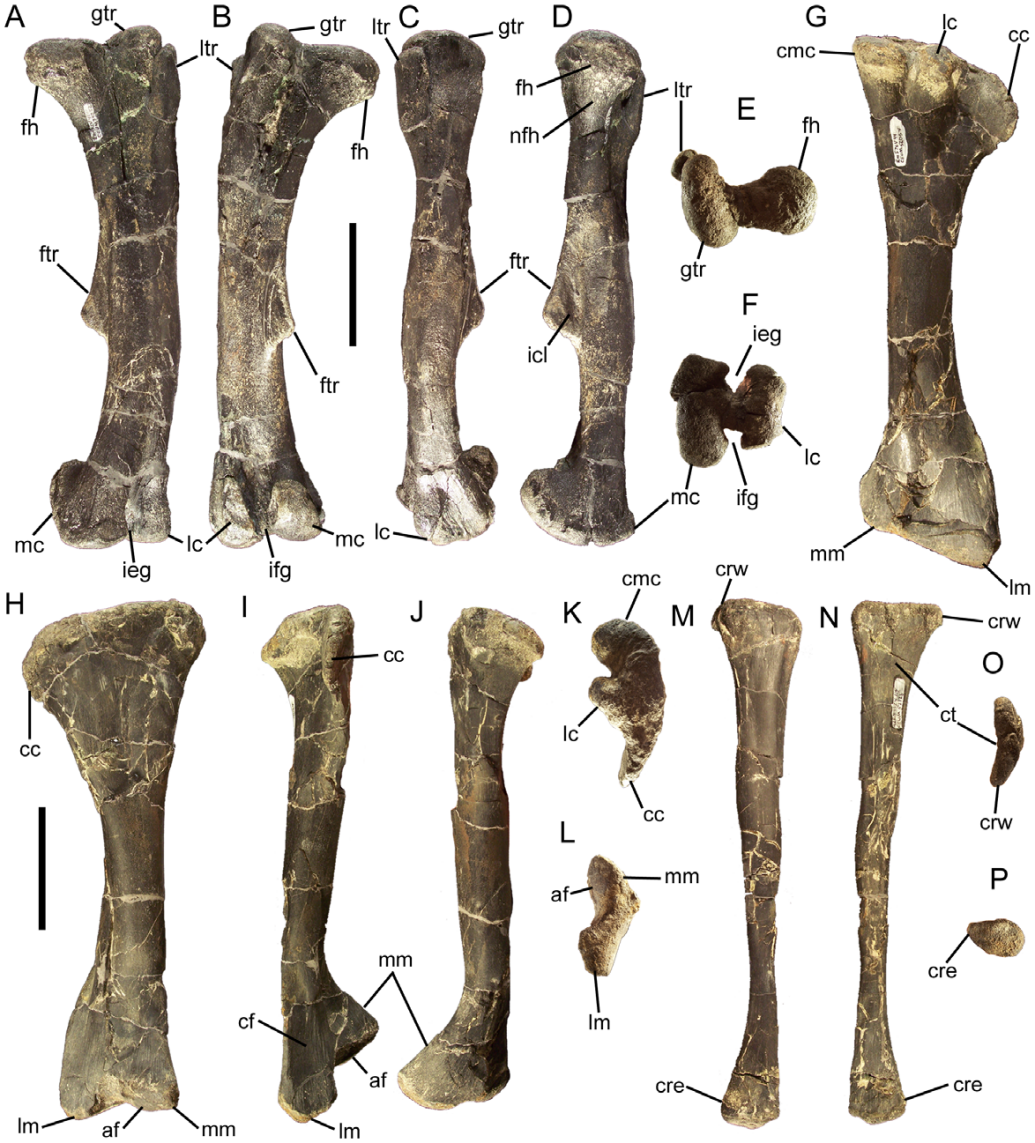
Hindlimb elements of *Eolambia*. Left femur CEUM 34252 (Eo2) in (A) cranial, (B) caudal, (C) lateral, (D) medial, (E) proximal, and (F) distal views. Right tibia CEUM 52054 (WS8) in (G) lateral, (H) medial, (I) cranial, (J) caudal, (K) proximal, and (L) distal views. Left fibula CEUM 52126 (WS8) in (M) lateral and (N) medial views. Left fibula CEUM 35464 (Eo2) in (O) proximal and (P) distal views. *Abbreviations*: *af*, facet for articulation of astragalus; *cc*, cnemial crest; *cf*, concavity for articulation of distal end of fibula; *cmc*, caudomedial condyle; *cre*, cranial expansion of distal end of fibula; *crw*, cranial wing on proximal end of fibula; *ct*, concave surface for articulation with lateral and caudomedial condyles of tibia; *fh*, femoral head; *ftr*, fourth trochanter; *gtr*, greater trochanter; *icl*, insertion pit of *M. caudifemoralis longus*; *ieg*, intercondylar extensor groove; *ifg*, intercondylar flexor groove; *lc*, lateral condyle; *lm*, lateral malleolus; *ltr*, lesser trochanter; *mc*, medial condyle; *mm*, medial malleolus; *nfh*, neck of femoral head. Scale bars equal 10 cm.

The femur is bowed laterally in cranial and caudal views (Fig. 32A). The distal femoral shaft does not curve caudally, but rather is straight in lateral and medial views (Fig. 32C). The rounded head of the femur is medially directed and supported ventrally by a narrow neck (Fig. 32A, B, D, E). Lateral to the femoral head, the proximal surface of the femur becomes craniocaudally narrow, forming a saddle-like region between the femoral head and the greater trochanter (Fig. 32E). This saddle-like region slopes proximolaterally to form the greater trochanter. The greater trochanter is a craniocaudally elongate and mediolaterally compressed rounded ridge with a gently convex lateral margin (Fig. 32A–C, E). The lesser trochanter is a mediolaterally compressed flange craniolateral to the greater trochanter (Fig. 32A, C, D, E). The cranial and caudal edges of the lesser trochanter are straight, whereas the lateral surface of the lesser trochanter is slightly convex. In lateral and medial views, the base of the lesser trochanter is cranially offset from the femoral shaft distal to it (Fig. 32C, D). Only a shallow cleft separates the greater and lesser trochanters; for most of its length, the lesser trochanter is closely appressed to the craniolateral surface of the proximal end of the femur (Fig. 32A, C). The fourth trochanter is located approximately halfway down the shaft of the femur (Fig. 32A–D). The fourth trochanter is not pendant as in more basal ornithopods, but rather is proximodistally broad and forms a scalene triangle, as in *Planicoxa* (DMNH 40917), *Hypselospinus* (NHMUK R1629 [41]), *Iguanodon bernissartensis* [29], *Mantellisaurus* [30], *Ouranosaurus* [26], *Jinzhousaurus* [56], “*Probactrosaurus*” *mazongshanensis* [42], *Probactrosaurus gobiensis* [13], and *Nanyangosaurus* [43]. The medial surface of the fourth trochanter bears a distinct elliptical pit, with its long axis oriented proximodistally (Fig. 32D); this pit marks the insertion of *M. caudifemoralis longus*.

The distal end of the femur is expanded craniocaudally and mediolaterally and divided into two condyles by the cranial intercondylar extensor groove and caudal intercondylar flexor groove (Fig. 32A–D, F). The medial condyle is considerably bigger than the lateral condyle, with an inflated, mediolaterally thick caudal extension; the caudal extension of the lateral condyle is smaller and mediolaterally compressed (Fig. 32B–D, F). The intercondylar extensor groove is deep, U-shaped, and partly enclosed by a medial expansion of the lateral condyle and a lateral expansion of the medial condyle (Fig. 32F). The intercondylar flexor groove is also deep and is partly enclosed by a lateral bulge of the medial condyle (Fig. 32F).

### Tibia

The tibia is expanded craniocaudally at its proximal end and mediolaterally at its distal end (Fig. 32G–J). The shaft of the tibia is straight in all views (Fig. 32G–J), but does twist craniolaterally towards the proximal end of the bone, giving rise to the cnemial crest. The proximal surface of the tibia is highly rugose, indicating the presence of a cartilaginous cap between it and the distal end of the femur (Fig. 32K). The cnemial crest curves craniolaterally and is mediolaterally compressed and blade-like, with a rounded cranial margin (Fig. 32G–I, K). Caudal to the cnemial crest there are two bulbous condyles with which the proximal end of the fibula would articulate (see below) (Fig. 32G, K). The lateral condyle projects caudolaterally; the caudomedial condyle also projects caudolaterally and is larger than the lateral condyle (Fig. 32G, K). The lateral and caudomedial condyles are separated by a deep, narrow cleft (Fig. 32K).

The distal end of the tibia is distinctly asymmetrical, with a pronounced step between the lateral and medial malleoli (Fig. 32G–I). The lateral malleolus projects farther distally than the medial malleolus and is gently convex along its distal margin to fit into concave facets on the proximal surfaces of the astragalus and calcaneum (see below) (Fig. 32G–I). The craniolateral surface of the lateral malleolus bears a striated shallow concavity that forms the articulation surface for the distal end of the fibula (Fig. 32I). The medial malleolus is triangular with a convex distal margin (Fig. 32G–J). The medial malleolus curves craniomedially and bears a facet on its craniodistal surface for articulation with the astragalus (Fig. 32H, I, L).

### Fibula

The proximal and distal ends of the fibula are expanded craniocaudally and the shaft is straight (Fig. 32M, N). The cranial margin of the proximal end exhibits a cranially-directed rounded wing in lateral and medial views (Fig. 32M, N). The lateral surface of the proximal end is convex, whereas the medial surface is concave to articulate with the lateral and caudomedial condyles on the proximal end of the tibia (Fig. 32M–O). The distal end of the fibula has a convex and rugose distal surface to fit into the concave fibula facet on the proximal surface of the calcaneum (see below) (Fig. 32M, N, P). The distal end of the fibula bears a mediolaterally narrow cranial expansion (Fig. 32M, N, P).

### Tarsus

**Figure 33:**
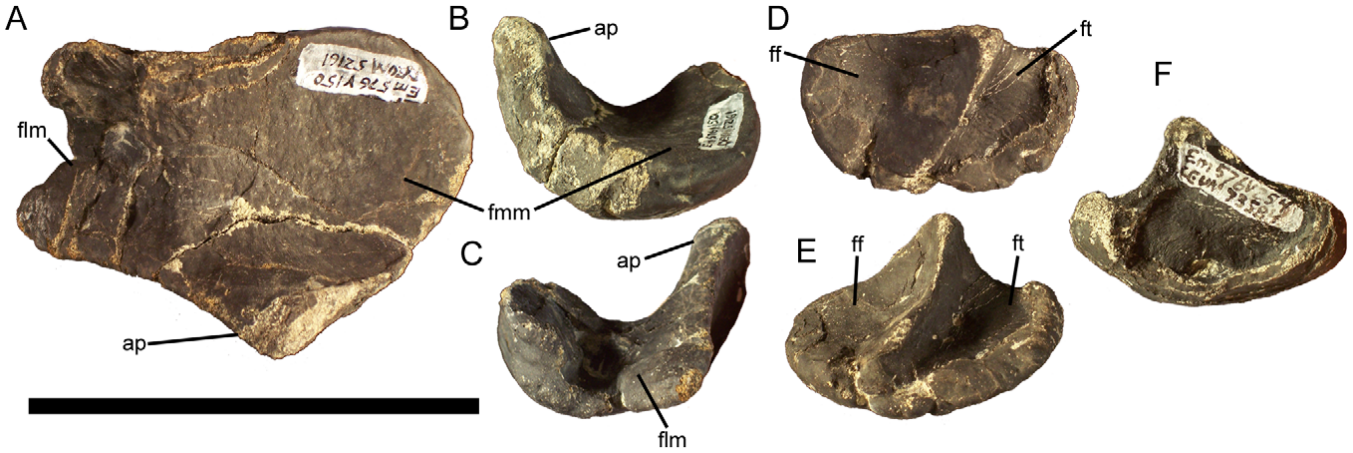
Tarsal elements of *Eolambia*. Right astragalus CEUM 52161 (WS8) in (A) proximal, (B) medial, and (C) lateral views. Right calcaneum CEUM 73584 (WS8) in (D) proximal, (E) medial, and (F) lateral views. *Abbreviations*: *ap*, ascending process; *ff*, facet for distal end of fibula; *flm*, facet for lateral malleolus of tibia; *fmm*, facet for medial malleolus of tibia; *ft*, facet for lateral malleolus of tibia. Scale bar equals 10 cm.

The proximal surface of the astragalus bears two facets, a medial and a lateral, for articulation with the distal end of the tibia. The medial facet is broader craniocaudally and mediolaterally than the lateral facet and would receive the enlarged medial malleolus of the tibia (Fig. 33A, B). The lateral facet is mediolaterally narrow and would receive the smaller lateral malleolus of the tibia (Fig. 33A, C). The lateral facet is distally offset relative to the medial facet, reflecting the morphology of the distal end of the tibia, on which the lateral malleolus is mediolaterally narrower and projects farther distally than the medial malleolus. The medial and lateral facets are separated by a low rounded craniocaudally-directed eminence on the proximal surface of the astragalus (Fig. 33A). The triangular ascending process projects proximally from the cranial margin of the proximal surface of the astragalus (Fig. 33A–C).

The proximal surface of the calcaneum is also divided into two distinct facets, a cranial and a caudal, for articulation with the distal ends of the tibia and fibula. The cranial facet is the larger of the two and would receive the distal end of the fibula (Fig. 33D, E). The caudal facet of the calcaneum, in conjunction with the lateral facet of the astragalus, would receive the lateral malleolus of the tibia. The cranial and caudal facets are separated by a sharp ridge that extends mediolaterally over the proximal surface of the calcaneum (Fig. 33D, E). This ridge increases in height from medial to lateral, reaching its apex at the lateral margin of the calcaneum. The lateral surface of the calcaneum is occupied almost entirely by a broad shallow depression surrounded by a raised lip (Fig. 33F).

### Metatarsals and pedal phalanges

As with the manus, there is not an articulated pes known for *Eolambia*. Other basal iguanodonts, such as *Camptosaurus dispar* (YPM 1877), *Hippodraco scutodens* (UMNH VP 20208) [7], *Iguanodon bernissartensis* (IRSNB 1534) [29], *Mantellisaurus atherfieldensis* (IRSNB 1551) [30], *Ouranosaurus nigeriensis* [26], and *Probactrosaurus gobiensis* [13], were used to identify the pedal elements of *Eolambia*.

**Figure 34:**
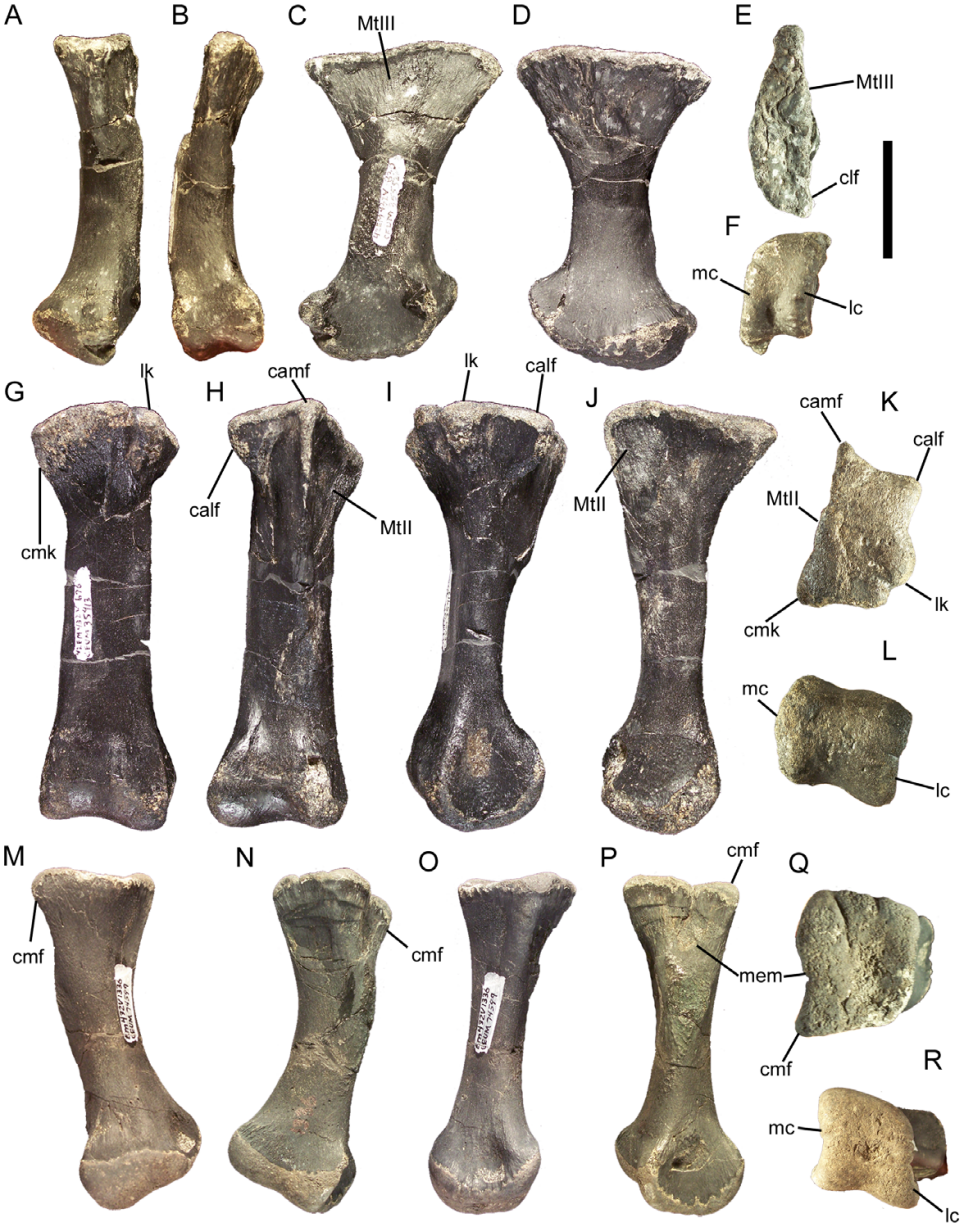
Metatarsals of *Eolambia*. Left metatarsal II CEUM 35552 (Eo2) in (A) cranial, (B) caudal, (C) lateral, (D) medial, (E) proximal, and (F) distal views. Left metatarsal III CEUM 35413 (Eo2) in (G) cranial, (H) caudal, (I) lateral, (J) medial, (K) proximal, and (L) distal views. Left metatarsal IV CEUM 74599 (Eo2) in (M) cranial, (N) caudal, (O) lateral, (P) medial, (Q) proximal, and (R) distal views. *Abbreviations*: *calf*, caudolateral flange that would overlap metatarsal IV; *camf*, caudomedial flange on contact with metatarsal II; *clf*, craniolateral flange that would overlap craniomedial knob on metatarsal III; *cmf*, craniomedial flange that would overlap metatarsal III; *cmk*, craniomedial knob on metatarsal III; *lc*, lateral condyle; *lk*, lateral knob that would fit into medial embayment on metatarsal IV; *mc*, medial condyle; *mem*, medial embayment that would receive lateral knob on metatarsal III; *MtII*, surface for metatarsal II; *MtIII*, surface for metatarsal III. Scale bar equals 5 cm.

Metatarsal II curves medially in cranial and caudal views (Fig. 34A, B). The proximal end is mediolaterally compressed, whereas the distal end is mediolaterally expanded. Both the proximal and distal ends are craniocaudally expanded in lateral and medial views (Fig. 34C, D). The lateral surface of metatarsal II is flat and striated near its proximal end, forming a surface for the articulation of metatarsal III (Fig. 34C). The medial margin of the proximal articular surface is convex in proximal view, whereas the lateral margin is straight (Fig. 34E). The cranial margin of the proximal articular surface bears a craniolaterally-directed flange that would partially overlap the craniomedial surface of the proximal end of metatarsal III (Fig. 34E). The distal articular surface is divided into two condyles by a broad furrow (Fig. 34B, F). The medial condyle extends farther caudally than the lateral.

In contrast to metatarsal II, the shaft of metatarsal III is straight (Fig. 34G, H). Both the proximal and distal ends are expanded mediolaterally in cranial and caudal views and craniocaudally in lateral and medial views (Fig. 34G–J). The proximal end of the metatarsal III is irregularly shaped, bearing a series of flanges and knobs that articulate with metatarsals II and IV (Fig. 34G–K). The medial surface of the proximal end exhibits a broad flat facet for articulation with the flat lateral surface of the proximal end of metatarsal II (Fig. 34H, J, K). The craniomedial margin of the proximal articular surface bears an enlarged knob that would rest against the aforementioned craniolateral flange on the proximal end of metatarsal II (Fig. 34G, K). The articulation surface for metatarsal II is expanded farther caudally by a tapering flange on the caudomedial margin of the proximal articular surface (Fig. 34H, K). The lateral surface of the proximal end of metatarsal III bears another large knob that would fit into the embayment on the medial surface of the proximal end of metatarsal IV (Fig. 34G, I, K) (see below). Caudal to this knob is a large triangular flange that would partially overlap the caudomedial surface of the proximal end of metatarsal IV (Fig. 34H, I, K). As with metatarsal II, the distal articular surface of metatarsal III is divided into two condyles by a wide shallow furrow (Fig. 34G, H, L). The medial margin of the medial condyle is convex, whereas the lateral margin of the lateral condyle is concave in distal view (Fig. 34L). The lateral condyle projects farther caudally than the medial condyle.

Metatarsal IV is strongly curved laterally in cranial and caudal views (Fig. 34M, N). Both the proximal and distal ends are expanded mediolaterally in cranial and caudal views and craniocaudally in lateral and medial views (Fig. 34M–P). The proximal end of metatarsal IV is irregularly shaped and complements the morphology of the proximal end of metatarsal III (Fig. 34M–Q). The craniomedial surface of the proximal end bears a medially-directed flange that would partially overlap the craniolateral surface of the proximal end of metatarsal III (Fig. 34M, N, P, Q). Caudal to this flange is a broad embayment that would receive the aforementioned knob on the lateral surface of the proximal end of metatarsal III (Fig. 34P, Q). The distal articular surface of metatarsal IV is also divided into two condyles by a furrow, though this feature is less distinct than those of metatarsals II and III (Fig. 34M, N, R).

**Figure 35:**
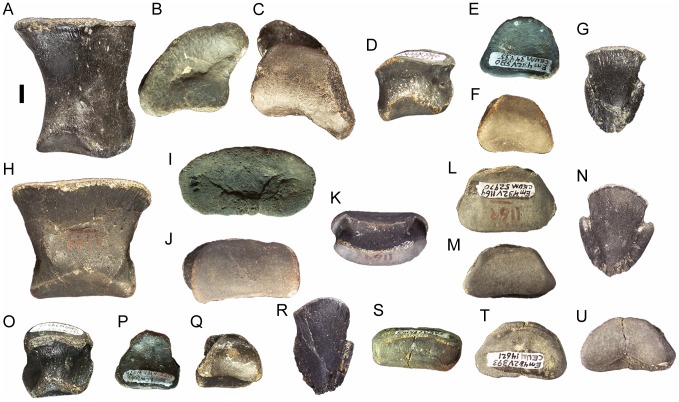
Pedal elements of *Eolambia*. Left phalanx 1 of digit II CEUM 35474 (Eo2) in (A) dorsal, (B) proximal, and (C) distal views. Left phalanx 2 of digit II CEUM 34435 (Eo2) in (D) dorsal, (E) proximal, and (F) distal views. Left ungual of digit II CEUM 35741 (Eo2) in (G) dorsal view. Right phalanx 1 of digit III CEUM 53000 (Eo2) in (H) dorsal, (I) proximal, and (J) distal views. Left or right phalanx 2 of digit III CEUM 52970 (Eo2) in (K) dorsal, (L) proximal, and (M) distal views. Left or right ungual of digit III CEUM 74547 (Eo2) in (N) dorsal view. Right phalanx 1 of digit IV CEUM 34352 (Eo2) in (O) dorsal, (P) proximal, and (Q) distal views. Left ungual of digit IV CEUM 14415 (Eo2) in (R) dorsal view. Distal phalanx CEUM 14621 (Eo2) of pedal digit III or IV in (S) dorsal, (T) proximal, and (U) distal views. In all images of elements in proximal or distal view, dorsal is towards the top of the figure and plantar towards the bottom. Scale bar equals 1 cm.

Phalanx 1 of digit II is a stout bone with very broad proximal and distal articular surfaces (Fig. 35A). The proximal articular surface bears three flanges, a dorsomedial, a plantomedial, and a plantolateral (Fig. 35B). The distal articular surface exhibits a prominent plantolateral flange (Fig. 35C). Phalanx 2 of digit II is proximodistally much shorter for its mediolateral width than phalanx 1 (Fig. 35D). The distal articular surface extends onto the dorsal surface of the phalanx. The proximal and distal articular surfaces are both subtriangular (Fig. 35E, F). The ungual of digit II is much like the manual unguals and the unguals of pedal digits III and IV (see below) in being broad, flat, and hoof-like, as in *Nanyangosaurus* [43], *Bactrosaurus* [38], *Levnesovia* [36], *Tanius* [44], *Tethyshadros* [37], *Lophorhothon* [53], and hadrosaurids [48]. The rugose lateral margin is more abruptly offset from the proximal end than the medial margin (Fig. 35G).

Phalanx 1 of digit III is like that of digit II in its robustness, but differs in being approximately symmetrical (Fig. 35H). The proximal and distal articular surfaces are elliptical (Fig. 35I, J). As is the case in pedal digit II, phalanx 2 of digit III is proximodistally quite short compared to phalanx 1 (Fig. 35K). Both the proximal and distal articulation surfaces are mediolaterally narrower along their dorsal margins than along their plantar margins (Fig. 35L, M). The ungual of digit III differs from those of digits II and IV in being symmetrical (Fig. 35N).

Phalanx 1 of digit IV is robust like the first phalanges of digits II and III, but with a more attenuated dorsal surface, giving the lateral and medial surfaces a puckered appearance (Fig. 35O). Both the proximal and distal articulation surfaces are mediolaterally narrower along their dorsal margins than along their plantar margins (Fig. 35P, Q). The ungual of digit IV resembles that of digit II in being asymmetrical, with a more prominent lateral rugose surface (Fig. 35R). However, the ungual of digit IV is more tapered than that of digit II.

The distal phalanges of the manual and pedal digits are difficult to identify to a specific digit. They resemble the distal phalanges of other basal iguanodonts in being proximodistally short compared to the more proximal phalanges and having semicircular articulation surfaces (Fig. 35S–U).

## Discussion

*Eolambia caroljonesa* is by far the most abundant dinosaur in the Mussentuchit Member of the Cedar Mountain Formation, known from at least eight isolated, associated individuals and two bonebeds (MNI = 12 in the Eo2 bonebed, and MNI = 4 in the WS8 bonebed). Other dinosaur specimens from the Mussentuchit Member include the holotype partial skeleton of the nodosaurid *Animantarx ramaljonesi* [60], an associated theropod skeleton [61], and teeth of tyrannosauroids, dromaeosaurids, troodontids, hesperornithiform birds, sauropods, basal ornithopods, pachycephalosaurs, and ceratopsians [61].

Two of the six characters that comprise the unique combination of features diagnostic of *Eolambia caroljonesa* can be observed only in juvenile specimens (marked with an asterisk in ‘Diagnosis’). Thus, it is essential to explore whether these characters might change through ontogeny. Most other basal hadrosauroids, e.g., *Equijubus*, *Jinzhousaurus*, *Protohadros*, and *Jeyawati*, are known from a single specimen, and thus do not reveal a great deal regarding ontogenetic changes. However, *Bactrosaurus johnsoni* is known from multiple adult and juvenile specimens, allowing comparison with *Eolambia*. The expansion of the coronoid process (along only rostral margin versus along rostral and caudal margins; character 22 in [62]) does not vary with ontogeny in *Bactrosaurus*, as shown by juvenile (AMNH 6380 and 6581) and adult (AMNH 6553) dentaries. However, it must be noted that the codings of *Eolambia* and *Bactrosaurus* differ for this character [62]; *Eolambia* is coded as 22^0^ (coronoid process expanded along only rostral margin), whereas *Bactrosaurus* is coded as 22^1^ (coronoid process expanded along rostral and caudal margins). Although the derived condition is present in juvenile and adult *Bactrosaurus*, it is possible that juvenile *Eolambia* retained the primitive state and that an adult *Eolambia* would have a coronoid process expanded along both margins. Unfortunately, the only known adult dentary of *Eolambia*, that of holotype CEUM 9758, is incomplete in the relevant region of the coronoid process. The potential ontogenetic variation of the other feature (119^2^, straight shaft of ischium) is also difficult to assess, because the known juvenile ischium of *Bactrosaurus* (AMNH 6577 [63]) is missing its shaft, as is the only known adult ischium of *Eolambia*, that of holotype CEUM 9758.

**Figure 36:**
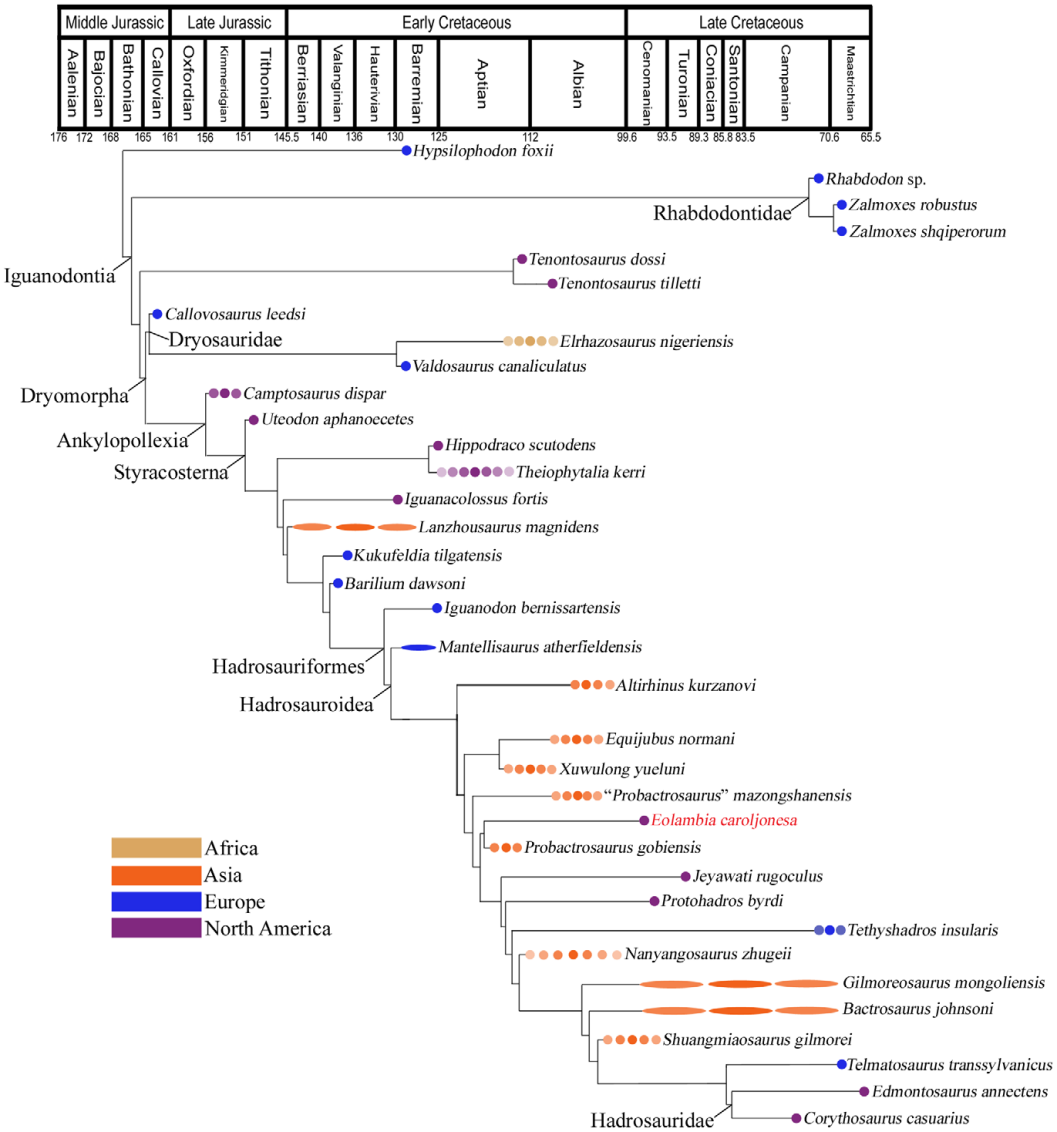
Phylogenetic relationships of *Eolambia*. Time-calibrated maximum agreement subtree of 16,270 most parsimonious trees obtained by the analysis of McDonald [62]. Timescale after Walker and Geissman [16]. Uncertainty in taxon ages indicated by lighter circles or ellipses in the case of especially long ranges. Modified from McDonald [62].

The abundant material of *Eolambia* described herein supplements the descriptions of Kirkland [8] and Head [12] and allows the phylogenetic position of *Eolambia* to be investigated with greater confidence. Norman [13], [14] was the first to include *Eolambia* in a comprehensive phylogenetic analysis of basal iguanodonts. In the analysis of Norman from 2004 [14], *Eolambia* is coded as unknown for characters related to the morphology of the ventral condyle of the quadrate (character 18), the shape of the sternal (43), the shape of the cranial pubic process (55 and 56; “prepubic process” in Norman [14]), and various attributes of the femur (62–64). In the most recent global phylogenetic analysis of basal iguanodonts, that of McDonald [62], *Eolambia* can be coded for similar characters (64^0^, 96^1^, 115^1^, 116^1^, 121^1^, 123^1^, 127^3^) related to these structures based upon specimens described herein. The analysis of Norman [14], found *Eolambia* to be the sister taxon of *Altirhinus*, a basal hadrosauroid from the late Early Cretaceous of Mongolia [32]. The phylogenetic analyses of Sues and Averianov [36] and Prieto-Márquez [64], which focused on Hadrosauridae but included numerous non-hadrosaurid hadrosauroids, placed *Eolambia* as the sister taxon of *Fukuisaurus* and *Protohadros*, respectively. However, McDonald [62] recovered *Eolambia* as a more derived hadrosauroid, sister taxon to *Probactrosaurus gobiensis* from the Early Cretaceous of China [13] (Fig. 36). This relationship is supported by two ambiguous synapomorphies (62^1^: quadrate straight for much of its length but curved caudally near its dorsal end; 100^2^: straight caudal margin of scapula, dorsal and ventral margins are parallel approaching caudal margin of scapula and meet caudal margin at nearly right angles).

The close relationship between *Eolambia* and *Probactrosaurus gobiensis* found by the analysis of McDonald [62] has interesting paleobiogeographic implications. Based upon the age and composition of the fauna from the Mussentuchit Member, Cifelli et al. [65] proposed an influx of new dinosaurian groups from Asia in the Cenomanian, of which *Eolambia* was a component. If *Eolambia* is indeed most closely related to an Asian taxon, such as *Fukuisaurus* or *Probactrosaurus* (Fig. 36), then this lends considerable support to this hypothesis. However, the global phylogenetic and biogeographic analyses of Prieto-Márquez [64], [66], in which *Eolambia* is the sister taxon of *Protohadros* from Texas and more derived than *Probactrosaurus*, indicated that the common ancestor of (*Eolambia*+*Protohadros*) and more derived hadrosauroids was distributed across Asia and North America.

It is possible that *Eolambia* supplanted *Tenontosaurus* as the most abundant large herbivore in the Western Interior of North America. However, although it is well known from Lower Cretaceous units throughout the Western Interior [67]–[69], *Tenontosaurus* is as yet poorly represented in the Cedar Mountain Formation (Ruby Ranch Member [15]). Furthermore, *Eolambia* is known only from the Mussentuchit Member in eastern Utah. Further iguanodontian remains from the Mussentuchit Member, underlying Ruby Ranch Member, and other Aptian–Cenomanian units in the Western Interior will be necessary to fully explore this scenario.

## Materials and Methods

All specimens of *Eolambia* described in this paper are reposited at the CEUM, and all were examined, photographed, and measured at that institution.

## Supporting Information

Table S1 **Table of measurements.**
(DOC)
