# Supplementary material for: Osteology of the Basal Hadrosauroid Eolambia caroljonesa (Dinosauria: Ornithopoda) from the Cedar Mountain Formation of Utah
Source: PLoS One. 2012 Oct 15;7(10):e45712. doi: 10.1371/journal.pone.0045712 (PMC3471925; doi:10.1371/journal.pone.0045712)
Supplement: Table S1 — Table of measurements. (DOC) [file pone.0045712.s001.doc]

**S1.** Measurements of select elements of *Eolambia caroljonesa*. Measurements are given in centimeters unless otherwise noted.

| **Specimens** | **Measurements** |
| --- | --- |
| **CEUM 13355, predentary (Eo2)** |  |
| Total rostrocaudal length from rostral face of the median marginal denticle to the caudal ends of the lateral processes. | 4.4 |
| Width across caudal ends of the lateral processes. | 6.5 |
| **CEUM 9758, right dentary (holotype)** |  |
| Total rostrocaudal length measured along ventral margin. | ~39.0 |
| Rostrocaudal length of symphysis. | 3.9 |
| Length of diastema, measured from caudal-most point of predentary groove to rostral margin of the first alveolus. | 6.9 |
| Rostrocaudal length of tooth row. | ~27.0 |
| Dorsoventral depth at midpoint. | 7.6 |
| **CEUM 34357, left dentary (Eo2)** |  |
| Total rostrocaudal length measured along ventral margin. | 20.7 |
| Rostrocaudal length of symphysis. | 3.0 |
| Length of diastema, measured from caudal-most point of predentary groove to rostral margin of the first alveolus. | 4.5 |
| Rostrocaudal length of tooth row. | 15.0 |
| Dorsoventral depth at midpoint. | 4.4 |
| Dorsoventral height of coronoid process, measured from lateral shelf to apex. | 6.1 |
| Greatest rostrocaudal width of coronoid process. | 2.2 |
| **CEUM 35525, left surangular (Eo2)** |  |
| Total rostrocaudal length measured along ventral margin. | 8.7 |
| Rostrocaudal length of angular groove. | 6.4 |
| Rostrocaudal length of glenoid. | 3.7 |
| **CEUM 36073, left surangular (Eo2)** |  |
| Mediolateral width of glenoid. | 2.6 |
| Rostrocaudal length of glenoid. | 5.0 |
| **CEUM 9758, left maxilla (holotype)** |  |
| Preserved rostrocaudal length along ventral margin. | ~30.0 |
| Rostrocaudal length of ectopterygoid shelf. | 8.8 |
| Rostrocaudal length of jugal process. | 6.0 |
| **CEUM 35492, right maxilla (Eo2)** |  |
| Total rostrocaudal length along ventral margin. | 19.1 |
| Rostrocaudal length of base of ascending process. | 8.4 |
| Rostrocaudal length of ectopterygoid shelf. | 5.8 |
| Rostrocaudal length of jugal process. | 3.4 |
| **CEUM 34356, right maxilla (Eo2)** |  |
| Rostrocaudal length of base of ascending process. | 9.6 |
| Maximum dorsoventral height of ascending process. | 1.7 |
| Rostrocaudal length of ectopterygoid shelf. | 5.1 |
| Rostrocaudal length of jugal process. | 3.0 |
| **CEUM 52204, left jugal (WS8)** |  |
| Total rostrocaudal length along ventral margin. | 14.5 |
| Dorsoventral height of postorbital process along its caudal margin. | 5.8 |
| Rostrocaudal length of maxilla slot. | 5.7 |
| **CEUM 52851, right quadrate (Eo2)** |  |
| Dorsoventral height along caudal margin. | 12.5 |
| Maximum mediolateral width of ventral condyle. | 2.9 |
| Maximum rostrocaudal length of ventral condyle. | 2.0 |
| Maximum mediolateral width of dorsal condyle. | 1.5 |
| Maximum rostrocaudal length of dorsal condyle. | 9.0 millimeters |
| Maximum dorsoventral height of quadratojugal notch in lateral wing. | 2.3 |
| **CEUM 9758, left frontal (holotype)** |  |
| Maximum mediolateral width. | 9.6 |
| Maximum rostrocaudal length. | 11.1 |
| **CEUM 35339, parietal (Eo2)** |  |
| Rostrocaudal length down midline. | 6.7 |
| Minimum mediolateral width. | 2.5 |
| Maximum dorsoventral depth. | 2.5 |
| Rostrocaudal length of sagittal crest. | 3.9 |
| **CEUM 35475, braincase (Eo2)** |  |
| Width across basal tubera. | 4.4 |
| Maximum mediolateral width of occipital condyle. | 3.5 |
| Maximum dorsoventral depth of occipital condyle. | 2.0 |
| Maximum mediolateral width of foramen magnum. | 2.6 |
| Maximum dorsoventral height of foramen magnum. | 2.4 |
| Preserved length of left paroccipital process along dorsal margin. | 9.3 |
| **CEUM 34261, dentary tooth (Eo2)** |  |
| Maximum mesiodistal width of crown. | 11.0 millimeters |
| Maximum apicobasal height of crown. | 25.0 millimeters |
| **CEUM 52062, right sternal (WS8)** |  |
| Total craniocaudal length. | 15.2 |
| Craniocaudal length of caudolateral process. | 6.8 |
| Maximum mediolateral width. | 6.6 |
| **CEUM 52097, right scapula (WS8)** |  |
| Total craniocaudal length. | 44.0 |
| Craniocaudal length of glenoid. | 5.6 |
| Maximum dorsoventral depth of cranial end, measured from apex of scapular labrum to apex of acromion process. | 12.9 |
| Dorsoventral depth of caudal end. | 7.3 |
| **CEUM 52831, right coracoid (WS8)** |  |
| Total craniocaudal length. | 9.0 |
| **CEUM 52125, left humerus (WS8)** |  |
| Total proximodistal length. | 27.0 |
| Diameter at midpoint. | 4.8 |
| Mediolateral width of proximal end. | 9.9 |
| Mediolateral width of distal end. | 6.7 |
| Proximodistal length of deltopectoral crest. | 11.6 |
| **CEUM 52162, right ulna (WS8)** |  |
| Diameter at midpoint. | 2.4 |
| Proximodistal length. | 25.0 |
| **CEUM 52163, right radius (WS8)** |  |
| Diameter at midpoint. | 1.8 |
| Proximodistal length. | 20.5 |
| **CEUM 52052, right metacarpal III (WS8)** |  |
| Diameter at midpoint. | 1.5 |
| Proximodistal length. | 9.6 |
| **CEUM 52100, left metacarpal IV (WS8)** |  |
| Proximodistal length. | 9.1 |
| **CEUM 52090, right ilium (WS8)** |  |
| Total craniocaudal length along dorsal margin. | 43.0 |
| Craniocaudal length of preacetabular process along dorsal margin. | ~18.0 |
| **CEUM 52152, right pubis (WS8)** |  |
| Maximum dorsoventral depth of cranial pubic process. | 10.5 |
| Craniocaudal length of cranial pubic process. | 25.5 |
| Proximodistal length of caudal pubic process. | 19.5 |
| **CEUM 74572, right ischium (Eo2)** |  |
| Maximum craniocaudal length of pubic peduncle. | 3.5 |
| Maximum mediolateral width of pubic peduncle. | 1.8 |
| Maximum craniocaudal length of iliac peduncle. | 7.2 |
| Maximum mediolateral width of iliac peduncle. | 3.6 |
| **CEUM 8786, left femur (near holotype)** |  |
| Proximodistal length. | ~81.0 |
| Diameter of femoral shaft immediately distal to fourth trochanter. | 13.2 |
| **CEUM 34252, left femur (Eo2)** |  |
| Proximodistal length. | 40.0 |
| Diameter of femoral shaft immediately distal to fourth trochanter. | 5.0 |
| **CEUM 35457, left femur (Eo2)** |  |
| Proximodistal length. | 37.5 |
| Diameter of femoral shaft immediately distal to fourth trochanter. | 5.9 |
| **CEUM 52054, right tibia (WS8)** |  |
| Proximodistal length. | 40.5 |
| Minimum diameter of shaft of tibia. | 4.7 |
| **CEUM 9758, left fibula (holotype)** |  |
| Proximodistal length. | ~64.0 |
| **CEUM 52126, left fibula (WS8)** |  |
| Proximodistal length. | 39.7 |
| **CEUM 52161, right astragalus (WS8)** |  |
| Total mediolateral width. | 8.4 |
| Total proximodistal height from apex of ascending process to distal articular surface. | 4.6 |
| Proximodistal height of ascending process from apex of process to floor of proximal articular surface. | 2.8 |
| **CEUM 73584, right calcaneum (WS8)** |  |
| Maximim mediolateral width. | 5.3 |
| Maximum proximodistal height. | 3.6 |
| **CEUM 35413, left metatarsal III (Eo2)** |  |
| Proximodistal length. | 15.5 |
| Breadth at midpoint of shaft. | 3.3 |
